# Phosphoproteome Profiling of SH-SY5y Neuroblastoma Cells Treated with Anesthetics: Sevoflurane and Isoflurane Affect the Phosphorylation of Proteins Involved in Cytoskeletal Regulation

**DOI:** 10.1371/journal.pone.0162214

**Published:** 2016-09-09

**Authors:** Joomin Lee, Eunsook Ahn, Wyun Kon Park, Seyeon Park

**Affiliations:** 1 Department of Food and Nutrition, Chosun University, Gwangju 61452, Korea; 2 Department of Applied Chemistry, Dongduk Women’s University, Seoul 02748, Korea; 3 Department of Anesthesia and Pain, College of Medicine, Department of Anesthesia and Pain, Yonsei University, Seoul 03722, Korea; University of Toledo, UNITED STATES

## Abstract

Inhalation anesthetics are used to decrease the spinal cord transmission of painful stimuli. However, the molecular or biochemical processes within cells that regulate anesthetic-induced responses at the cellular level are largely unknown. Here, we report the phosphoproteome profile of SH-SY5y human neuroblastoma cells treated with sevoflurane, a clinically used anesthetic. Phosphoproteins were isolated from cell lysates and analyzed using two-dimensional gel electrophoresis. The phosphorylation of putative anesthetic-responsive marker proteins was validated using western blot analysis in cells treated with both sevoflurane and isoflurane. A total of 25 phosphoproteins were identified as differentially phosphorylated proteins. These included key regulators that signal cytoskeletal remodeling steps in pathways related to vesicle trafficking, axonal growth, and cell migration. These proteins included the Rho GTPase, Ras-GAP SH3 binding protein, Rho GTPase activating protein, actin-related protein, and actin. Sevoflurane and isoflurane also resulted in the dissolution of F-actin fibers in SH-SY5y cells. Our results show that anesthetics affect the phosphorylation of proteins involved in cytoskeletal remodeling pathways.

## Introduction

Halogenated ether agents such as enflurane, desflurane, sevoflurane, and isoflurane are used as inhalation agents for clinical anesthesia. These anesthetics are inhaled and pass through the alveolocapillary membrane, diffusing into the blood, and finally reaching the central nervous system. The mechanism of general anesthesia has been presented and reviewed elsewhere [1, 2].

It has been suggested that ion channel proteins provide a site of action for inhalation agents to prolong the inhibitory channel activity of γ-aminobutyric acid type A (GABA_A_) and glycine receptors, and suppress the excitatory synaptic channel activity of nicotinic acetylcholine, serotonin, and glutamate receptors [3]. Anesthetics have been demonstrated to bind to different ion channels including serotonin receptors, nicotinic acetylcholine receptors, GABA_A_ receptors, glycine receptors, and glutamate receptors activated by N-methyl-D-aspartate (NMDA) or alphaamino-3-hydroxy-methyl-4-isoxazolepropionic acid (AMPA) [4, 5]. Inhalation agents have been shown to impair memory and learning and cause immobility at low concentrations by inhibiting nicotinic acetylcholine receptors, and to prevent movements by depressing the spinal cord function in decerebrate rats and goats [6–8]. In addition, inhalation agents have been observed to deactivate post-synaptic AMPA and NMDA receptors and activate glycine receptors, regardless of their action on GABA_A_ receptors in spinal motor neurons [9]. These reports demonstrate that anesthetics physiologically affect multiple sites leading to amnesia and immobility. Brain-level imaging using positron emission tomography and functional magnetic resonance imaging confirmed that inhalation agents decrease glucose metabolic activity, conforming to a reduction in synaptic activity [10, 11]. Therefore, the mechanism of action of inhalation agents is believed to comprise “complex effects by multiple mechanisms” [3].

Although the broad mechanism of general anesthesia has been reviewed, the exact mechanism of anesthetic action, especially at the molecular level, remains unknown. At the cellular level, the majority of external stimuli are detected by ion channel receptors distributed in the membrane of peripheral afferent fibers in neuronal pathways. Therefore, recognition by an ion channel receptor might play a crucial role in transducing an external signal, such as the one of an anesthetic located inside the cell. However, the intracellular pathways of nociception in anesthesia are still under investigation. Notably, the molecular events that are induced by anesthetics and lead to compensatory responses remain unresolved. To investigate the molecular mechanism of anesthesia, we hypothesized that, during exposure to clinical anesthesia, neural cells could functionally act as proximal sensors and mediators of subsequent events in the physiological mechanism of anesthetic action. Moreover, we hypothesized that anesthetic-perceptive phosphorylation may be related to ion gating as in the mechanism of anesthesia induction upon administration of potent halogenated anesthetics. This is because phosphorylation is involved in the regulation and transmission of information triggered by almost every type of external stimuli. The activation of membrane receptors could be coupled to changes in the phosphorylation pattern of representative determinants, leading to gating related to the mechanism of anesthetic action, thereby inhibiting excitatory channel activity and prolonging the activation of inhibitory channels such as GABA_A_ and glycine receptors [12]. Phosphorylation is an important posttranslational protein modification that is essential for signal transduction mediated by a large number of proteins, leading to the regulation of cell cycle and metabolism, cell differentiation, and development [13, 14]. Kinases/phosphatases encompass approximately 2% of the human genome, and one every three proteins is phosphorylated at a particular stage in its life [13]. The primary role of phosphorylation is to act as a switch to turn "on" or "off" the activity of a protein or a cellular pathway, in an acute and reversible manner [15, 16]. Analyzing differential gene expression is insufficient for the investigation of immediate early responses integrating multiple physiological inputs into highly complex and dynamic phosphorylation events that are not captured at the transcriptional level [12]. Therefore, identifying a subset of specific phosphoproteins is an essential prerequisite to understand the regulatory role of anesthetics, especially concerning short-term activational on/off changes [12]. We aimed to obtain a phosphoproteome profile that is altered upon treatment with an inhalation anesthetic-sevoflurane in the present study-guided by the hypothesis that receptor activation induced by anesthetics could lead to changes in the phosphorylation pattern of representative determinants coupled with ion gating as in the mechanism of anesthesia induction. We used the neuroblastoma cell line SH-SY5y because neural cells are exposed to external anesthetic stimuli and mediate compensatory signals in physiological mechanisms of anesthetic action. Because changes in protein phosphorylation are immediate processes driven by various types of stimuli, the influence of time should be considered. Therefore, we investigated time-dependent changes in the cellular protein phosphorylation profile upon treatment with sevoflurane.

## Materials and Methods

### Cell culture and treatment

The human neuroblastoma SH-SY5y cell line was purchased from the Korean Cell Line Bank and cultured in a humidified incubator with 5% carbon dioxide at 37°C. Dulbecco’s modified Eagle’s medium (DMEM) was supplemented with 10% fetal bovine serum (FBS) and antibiotics (Gibco-BRL, Rockville, MD). For experiments, volatile isoflurane (Baxter, Deerfield, IL; density 1.50 g/mL, 184.5 g/mol, 100%) and sevoflurane (Abbott, Chicago, IL; density 1.52 g/mL, 200.1 g/mol, 100%) were used. For volatile anesthetic exposures, serum-containing medium was removed and cells were treated with 1 mM anesthetics in serum free media [17]. During exposures, cells were placed in a tight gas chamber (Ace Corp, Seoul, Korea) within the incubator.

For phsophoproteome profiling, SH-SY5y cells were incubated with 1 mM sevoflurane for 2 min, 5 min, 15 min, or 30 min; harvested and lysed. A 1 mM concentration of sevoflurane and isoflurane corresponds to approximately twice the minimum alveolar concentration for rats, and therefore represents a physiologically relevant concentration [18]. We confirmed the concentration of anesthetics in media using gas-liquid chromatography, and found that the aqueous concentration (0.93 and 0.61 mM for sevoflurane and isoflurane, respectively) was maintained during the experiment.

Inhibitors of P21-activated kinases (PAKs), IPA3 and FRAX597 were purchased from Selleckchem (Houston, TX). The specific PAK1 inhibitor IPA3 and an inhibitor of group 1 PAKs including PAK1, 2, and 3, FRAX597 were used in concentrations of 5 μM of 5nM, respectively. The corresponding concentrations were determined to be approximately twice the IC_50_ values reported by suppliers, which were 2.5 μM for IPA3 and 8–19 nM for FRAX597, respectively. Inhibitors were added for the last 30 min of the 12- to 15-h preincubation period, and cells were subsequently treated with sevoflurane or isoflurane for the indicated times.

### Total protein preparation

After cells were treated with anesthetics for the indicated times and washed with ice-cold PBS, pellets were homogenized using a motor-driven homogenizer (PowerGen125, Fisher Scientific, Waltham, MA) in a sample lysis solution containing 7 M urea, 2 M thiourea with 4% (w/v) 3-[(3-cholamidopropyl)dimethyammonio]-1-propanesulfonate (CHAPS), 1% (w/v) dithiothreitol (DTT), 2% (v/v) pharmalyte, 1X phosphatase inhibitor cocktail (Sigma-Aldrich, St Luis, MO), and 1X protease inhibitor cocktail (Sigma-Aldrich). Proteins were extracted for 1 h at room temperature with vortexing, and the extract was centrifuged at 15,000 × *g* for 1 h at 15°C. The insoluble material was discarded, and the soluble fraction was prepared for phosphoprotein isolation. The protein concentration of the final extract was measured using a BCA kit according to the manufacturer’s manual (BioRad, Hercules, CA).

### Preparation of phosphoprotein-enriched extracts

To isolate phosphoproteins from the total protein extracts, the PhosPro^TM^ Phosphoprotein enrichment kit (Genomine, Pohang, Korea) was used as described [12]. In brief, the total protein solution was diluted in dilution solution (Genomine) to a final concentration of 0.67 mg/mL. To generate a phosphoprotein-specific complex, 240 μL of solution A (Genomine) was added and rapidly and vigorously mixed by vortexing for a few seconds before 15 min of incubation with inversion or gentle vortexing [12]. After the subsequent addition of 360 μL of solution B (Genomine) and brief mixing, the sample solution was incubated for 15 min with gentle vortexing to settle the aggregated material. After approximately 4 mL of clear supernatant was discarded, the remaining aggregated suspension was transferred to a new tube and centrifuged at 15,000 × *g* for 5 min to allow precipitation of the phosphoprotein complex. The supernatant was discarded, and the aggregate in the hard pellet was dissolved in 0.7 mL of dissolving solution (Genomine). Subsequently, 750 μL of delipidation solution (methanol:chloroform, 600:150) was added while vortexing, and the solution was then centrifuged at 15,000 × *g* for 10 min. The middle phase protein disk was recovered avoiding the lower or upper phase solution. The protein disk was subsequently washed twice with approximately 1 mL of methanol. The protein pellet was completely air-dried or dried in an oven and dissolved in a solution for isoelectric focusing (IEF).

### Isoelectric focusing and two-dimensional gel electrophoresis

IEF and gel electrophoresis were performed as previously reported [12]. Proteins (200 μg) were dissolved in a final volume of 80 μL of sample buffer, composed of 7 M urea, 2 M thiourea, 4% (w/v) CHAPS, 100 mM DTT, and 0.5% carrier ampholyte (pH 4–7, Bio-Rad), and loaded on a 17 cm-long gel with a pH 4–7 gradient. An immobilized pH gradient immobiline polyacrylamide gel (IPG) strip was rehydrated and positioned with the gel side down on the strip tray. The voltage was sequentially increased from 150 to 3,500 V over 3 h to allow entry of the sample, followed by maintenance at 3,500 V, with focusing completed after 96 kV-h. Strips were then incubated for 10 min in equilibration buffer (50 mM Tris-Cl, pH 6.8 containing 6 M urea, 2% SDS, and 30% glycerol), firstly with 1% DTT and secondly with 2.5% iodoacetamide. Equilibrated strips were loaded onto SDS-PAGE gels (20 × 24 cm, 10–16%), and SDS-PAGE was performed using a Hoefer DALT 2D system (Amersham Biosciences, Buckinghamshire, UK) at 20°C and 1,700 V-h.

### 2DE gel image analysis

Following 2D electrophoresis (2DE), gels were fixed with a solution containing 40% (v/v) ethanol and 10% (v/v) acetic acid for 1 h, and stirred in a rehydration solution (5% (v/v) ethanol and 5% (v/v) acetic acid in distilled water) three times for 30 min. Proteins were visualized using a ProQ Diamond phosphoprotein gel stain (Invitrogen, Carlsbad, CA) for 1 h, and washed with ProQ Diamond phosphoprotein destaining solution (Invitrogen) for 30 min. Images were scanned using an image photographing equipment, exposed to a Cy3 emission filter for 20 sec, and analyzed by PDQuest 2D analysis software (BioRad). Subsequently, gels were stained with Coomassie Brilliant Blue G-250 (Invitrogen, Carlsbad, CA) and images were scanned. A Coomassie Brilliant Blue-stained image was used as a normalization control. Analysis of the spots was performed as previously described [12]. Standard spots stained with ProQ Diamond that matched between two samples were identified, and their intensities determined using an Image Master 2D system. A matching spot was identified as being quantitatively different only when it displayed the same degree of down- or upregulation in duplicate experiments; moreover, a matching spot between gels showed the same intensities of Coomassie Brilliant Blue staining in duplicate experiments. For all matched spots, the intensity value obtained from ProQ Diamond was normalized to each Coomassie Brilliant blue intensity value. For each spot, the intensity value obtained in the sevoflurane treatment group was divided by that obtained in the control gel. Spots showing expression levels that were five-fold lower or greater than those of the control were considered statistically significant differentially expressed protein species. The log values of these ratios (LR; the means and median values clustered around the 0 value) were calculated. An LR value close to 0 was expected if errors associated with the analysis were random and normally distributed. For in-gel enzymatic digestion, spots were excised from the destained gel and rehydrated in 10 ng/μL trypsin. Subsequently, the rehydrated spot was incubated on ice for 45 min and treated with 50 mM ammonium bicarbonate (10 μL) at 37°C for 12 h.

### Matrix-assisted laser-desorption ionization time-of-flight tandem mass spectroscopy (MALDI-TOF-MS/MS)

MALDI-TOF-MS/MS analysis was performed as previously described [12]. Following the desalting/concentration step on an mZipTipC18 column (Millipore, Billerica, MA) using acetonitrile as an eluent, digested samples were subjected to MALDI-TOF-MS/MS analysis. Peptide mixtures were loaded on the MALDI system using the dried-droplet technique and α-cyano-4-hydroxycinnamic acid as the matrix, and were analyzed using a 4700 Reflector spec #1 mass spectrometer (Applied Biosystems, Framingham, MA). Solubility of the matrix was tested using dimethylcyclodextrin (distributed from the Microbial Carbohydrate Resource Bank at Konkuk University, Korea). The Data Explorer software package (Applied Biosystems) was applied to match spots to the ProFound database.

### Immunoprecipitation

For immunoprecipitation, cells were treated with sevoflurane or isoflurane for the indicated times, and the total cell protein pool was extracted using lysis buffer (0.05 M Tris–HCl, pH 7.4, 0.15 M NaCl, 1% Noniodet P-40 (NP-40), 0.5 M PMSF, 50 μg/mL aprotinin, 10 μg/mL leupeptin, 50 μg/mL pepstatin, 0.4 mM sodium orthovanadate, 10 mM sodium fluoride, and 10 mM sodium pyrophosphate). After lysates were sonicated for 20 s and centrifuged at 15000 × *g* for 10 min, supernatants were used to determine protein concentration. Cell lysates (150 μg) were incubated with 2 μL of primary antibody (anti-HSP 90β antibody: Cell Signaling, Beverly, MA; anti-nucleolin antibody: Santa Cruz Biotechnology; anti-matrin 3 antibody: Abcam; anti-ras-GAP SH3 binding protein antibody: Abcam) overnight at 4°C. Next, 15 μL of Protein A/G plus (Gendepot, Barker, TX) was added, and the complex was incubated for 4 h at 4°C. The pellet was washed three times with lysis buffer, and immunoprecipitated complexes were released with 2× sample buffer for western blotting analysis using a phosphospecific antibody.

### Western blotting analysis

To confirm the 2D results obtained on the phosphorylation levels of HSP 90β, nucleolin, matrin 3, and the Ras-GAP SH3 binding protein, immunoprecipitated proteins were separated by SDS-PAGE. After separation, proteins were transferred onto a nitrocellulose membrane (Schleicher and Schuell, Keene, NH); the membrane was blocked by overnight incubation at 4°C with 5% (w/v) non-fat dry milk in Tris-buffered saline (TBS)–0.1% Tween-20 (TBST). The membrane was then incubated with an anti-phosphoserine/threonine/tyrosine antibody (Abcam) for 3 h. Following washing with TBST, the membrane was incubated with an anti-rabbit immunoglobulin coupled to peroxidase (Abcam). After a 60-min incubation at room temperature, the membrane was washed three times with TBST, and blots were developed using an enhanced chemiluminescence solution (ECL1; Amersham Biosciences,). Normalization was performed using a polyclonal GAPDH antibody (Santa Cruz Biotechnology). Blots were semi-quantified using a Gel Doc 2000 densitometer (BioRad).

### Fluorescent dye staining

Cells were cultured on coverslips for F-actin and G-actin staining with phalloidin-Alexa568 and DNAse I-Alexa488 (Molecular Probes, Eugene, OR), respectively. After cells were treated with 1 mM sevoflurane or isoflurane for 5, 15, and 30 min, the coverslips were washed with PBS, lightly fixed (4% paraformaldehyde for 10 min), and washed again. Cells were permeabilized with 0.05% Triton X-100 in phosphate-buffered saline (PBS) for 3 min at room temperature. After washing with PBS, cells were treated with 5 U/mL DNAse I-Alexa488 and phalloidin-Alexa568, and then mounted. Culture coverslips were placed on glass slides and imaged under a fluorescence microscope (Nikon Eclipse Ti-U, Tokyo, Japan) with a green fluorescent protein (GFP) filter set. Images were obtained and fluorescence intensity was analyzed using the NIS-Element BR 4.30.01 software (Nikon). Results were expressed as F-actin fluorescence relative to that of G-actin and statistical analysis was performed.

### Quantitative analysis

For the quantification of band intensity, blots were scanned using a Gel Doc 2000 densitometer (Bio-Rad, Hercules, CA). Statistical significance was determined using one-way ANOVA or the student *t*-test (SigmaPlot, LaJolla, CA), with data obtained from three or four independent experiments. Error bars represent the standard mean deviations.

## Results

### Phosphoprotein isolation and two-dimensional gel separation

**Fig 1:**
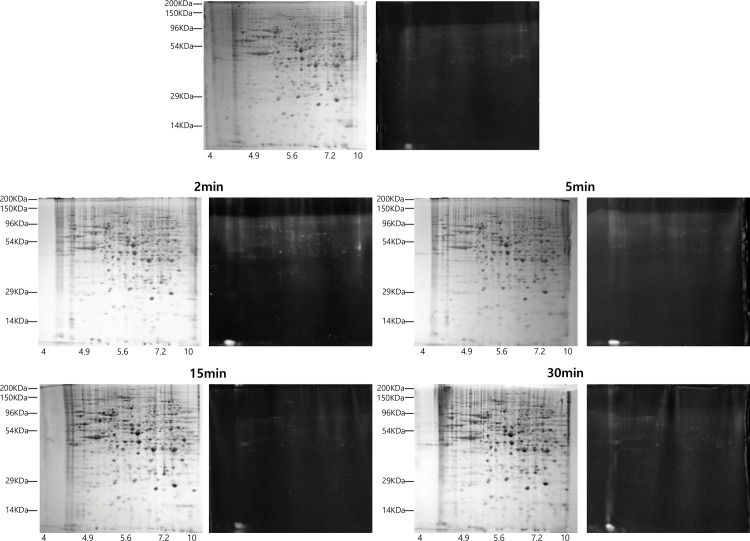
SH-SY5y cell phosphoprotein patterns following treatment with sevoflurane monitored by 2D gel electrophoresis. SH-SY5y cells were incubated with sevoflurane for 2, 5, 15, or 30 min; harvested and lysed. Intracellular enriched phosphoproteins were analyzed by 2D gel electrophoresis. Right panel: Coomassie Brilliant Blue staining; left panel: ProQ Diamond. Y axes represent the apparent molecular mass (kDa), and X axes represent pH values. Acquired images showed reproducibility of experiments. Data shown are representative of three separate experiments.

To investigate changes in phosphorylation associated with sevoflurane at the cellular level, the phosphoproteome in SH-SY5y cells after treatment with sevoflurane for 2, 5, 15, and 30 min was resolved by 2D gel electrophoresis. The resulting enriched phosphorylated proteins were stained with ProQ Diamond, and more than 20 phosphoprotein spots were obtained, as shown in Fig 1. Twenty-five protein spots showing differential expression levels between control and sevoflurane treatment were apparent. Table 1 lists these proteins, which were identified by MALDI-TOF MS/MS and fingerprinting. The proteome profiles of the study were presented in S1 File and S2 File.

**Table 1:** 

SSP	MW	PI	Intensity Fold Ratio (Sevoflurane-treatment/Control)				Log Ratio (Sevoflurane-treatment/Control)				Definition
			2min	5min	15min	30min	2min	5min	15min	30min
**310**	43.910	4.340	0.001	0.627	1.855	2.833	-3.184	-0.203	0.268	0.452	heterogeneous nuclear ribonucleoprotein C (C1/C2), isoform CRA_c, partial
**704**	103.280	4.340	1.000	1629.000	584.110	598.730	0.000	3.212	2.766	2.777	HSP90beta
**804**	124.970	4.380	1873.930	971.670	645.740	685.860	3.273	2.988	2.810	2.836	nucleolin, isoform CRA_c [Homo sapiens]
**1308**	43.790	4.380	6.833	4.323	7.034	3.216	0.835	0.801	0.847	0.507	heterogeneous nuclear ribonucleoprotein C (C1/C2), isoform CRA_c, partial
**1309**	46.140	4.620	1439.910	131.630	649.600	1761.950	3.158	3.120	3.217	3.246	Actin cytoplasmic 2
**1511**	56.320	4.610	17.619	2.379	8.130	17.014	1.246	1.093	1.208	1.231	Splicing factor 3A
**2310**	46.490	4.670	1542.760	151.880	2531.120	1314.080	3.188	3.179	3.185	3.119	Actin cytoplasmic 2
**2512**	56.120	4.670	2.285	2.886	7.491	6.436	0.359	0.460	0.875	0.809	Petidyl-prolyl isomerase
**2611**	70.110	4.690	1.000	1.000	3050.330	911.610	0.000	0.000	3.484	2.960	Ras-GAP SH3 binding protein [Homo sapiens]
**3102**	31.980	4.920	983.050	1.000	1.000	1.000	2.993	0.000	0.000	0.000	Chain A, Crystal Structure Of Full-Length Human Peroxiredoxin 4 In The Reduced Form
**3106**	32.870	4.900	2.410	1.285	1.863	2.037	0.382	0.268	0.270	0.309	serine/arginine-rich splicing factor 9 [Homo sapiens]
**3809**	126.520	5.070	1.000	1.000	3546.950	2050.240	0.000	0.000	3.550	3.312	Aldehyde oxidase
**4309**	45.150	5.830	0.937	0.655	0.591	0.474	-0.028	-0.183	-0.228	-0.324	C2orf54 protein
**4410**	49.520	5.460	2907.250	1374.560	1112.450	96.330	3.463	3.138	3.046	1.984	26S protease regulatory subunit 7 isoform 1 [Homo sapiens]
**4413**	47.540	5.840	0.626	0.467	1.264	0.200	-0.204	-0.331	-0.579	-0.699	HSP70
**4505**	53.310	5.360	4.329	4.141	4.489	0.905	0.636	0.617	0.652	-0.043	HNRPH1 [Homo sapiens]. heterogeneous nuclear ribonucleoprotein H
**4712**	89.200	5.480	2.821	1.634	1.292	0.002	0.450	0.213	0.111	-2.655	B-cell lymphoma 6 protein isoform 2 [Homo sapiens]
**4801**	126.230	5.270	1.000	1.000	2713.860	343.130	0.000	0.000	3.434	2.535	Matrin3
**5306**	46.630	6.440	1028.000	414.070	265.810	422.110	3.012	2.617	2.425	2.625	Chain A, Human Septin 2 In Complex With Gdp
**5402**	50.540	6.130	2.369	1.570	0.291	0.467	0.375	0.196	-0.537	-0.331	erbB3 binding protein EBP1 [Homo sapiens]
**5406**	50.600	6.480	2.505	0.774	0.228	0.415	0.399	-0.111	-0.642	-0.382	cell cycle protein p38-2G4 homolog [Homo sapiens]
**5407**	48.610	6.380	2.582	0.831	0.289	1.090	0.412	-0.080	-0.539	0.037	Rho GTPase activating protein 29
**5510**	64.580	6.130	645.020	592.430	360.790	676.790	2.810	2.773	2.820	2.830	lymphoid specific helicase variant7 [Homo sapiens]
**6401**	50.880	6.690	3.342	1.080	1.476	1.941	0.524	0.033	0.169	0.288	Rho GTPase
**6403**	48.370	6.820	2.889	0.004	0.951	0.687	0.461	-2.373	-0.022	-0.163	Actin-related protein

### Protein phosphorylation in sevoflurane-treated SH-SY5y cells

**Fig 2:**
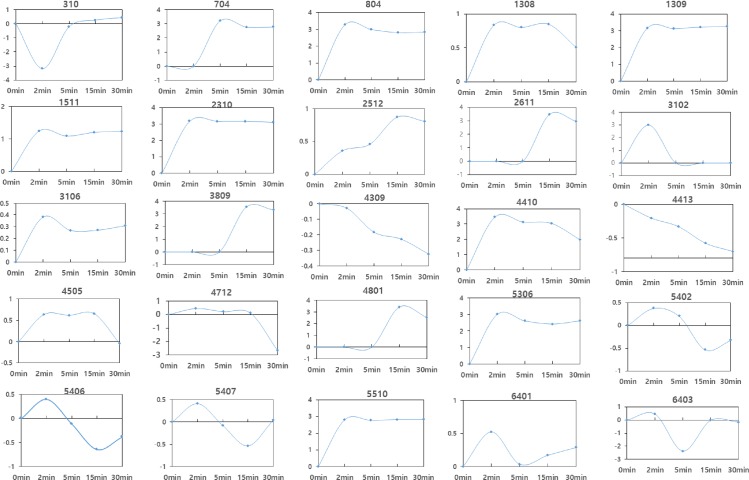
Typical kinetics of protein phosphorylation regulated in response to sevoflurane treatment in SH-SY5y cells. The kinetics of phosphorylation changes over time is depicted for selected proteins (Table 1). Each value represents single values from each gel. Lines connect average standardized abundance values. The Y axis represents the log ratio of treatment per control.

In our previous work, our experimental design for phosphoproteome analysis was formalized with a time course, in which replicated proteomes for each time point are technical replicates that have no particular relationship with each other [12]. Unequally spaced sampling intervals (2 and 3 min between the first two time points, and 15 min until the 30 min time point) with two replicates at each time point, were used, as shown in Fig 2. Similarities in phosphorylation behavior were observed for a group of proteins, including the heterogeneous nuclear ribonucleoprotein C (C1/C2, spot number 310 and 1308), the splicing factor 3A (1511), a petidyl-prolyl isomerase (2512), the serine/arginine-rich splicing factor 9 (3106), and the heterogeneous nuclear ribonucleoprotein H (4505). The line represents the trend estimation of the data with respect to time points, showing a few-fold increase after treatment, followed by a saturated trend, which is statistically distinct from random behavior.

A second group, including the B-cell lymphoma 6 protein isoform 2 (4712), erbB3 binding protein (EBP1, 5402), cell cycle protein p38-2G4 homolog (5406), Rho GTPase activating protein 29 (5407), Rho GTPase (6401), and an actin-related protein (6403) showed a few-fold increase in phosphorylation up to 2 min, and then returned to their minimum levels within 15 or 30 min after exposure.

For HSP90β (704), nucleolin (804), the 26S protease regulatory subunit 7 (4410), and septin 2 in complex with GDP (5306), phosphorylation levels reached a maximum of 1000-fold that of the control between 2 and 5 min, and gradually decreased. For peroxiredoxin 4 (3102), phosphorylation reached a maximum of 1000-fold that of the control after 2 min and drastically returned to its minimum level 5 min after exposure. For the Ras-GAP SH3 binding protein (2611), the aldehyde oxidase (3809), and matrin 3 (4801), phosphorylation levels reached their maximum at 1000-fold that of the control within 15 min, and then decreased.

In actin cytoplasmic 2 (2310) and the lymphoid specific helicase variant 7 (5510), phosphorylation was upregulated more than 1000-fold that of the control after sevoflurane exposure, followed by a saturated trend.

Finally, in the c2orf54 protein (4309) and HSP70 (4413) phosphorylation was downregulated to between half and a fifth that of the control after 15 min of exposure.

### Immunoprecipitaton and time profiling

**Fig 3:**
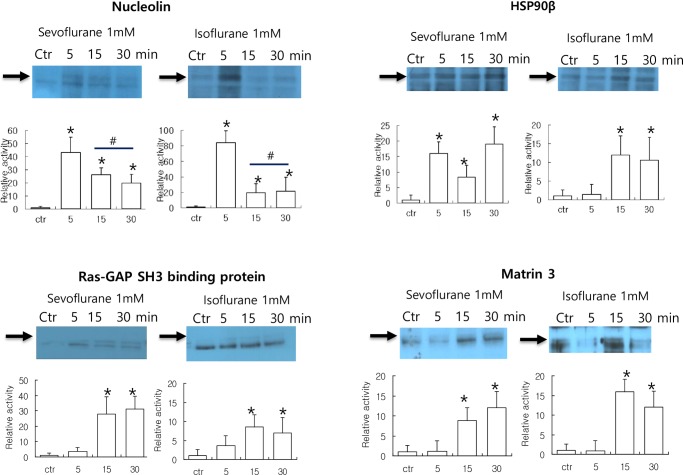
Phosphorylation levels of immunoprecipitated HSP90β, nucleolin, Ras-GAP SH3 binding protein, and matrin 3 in SH-SY5y cells exposed to sevoflurane and isoflurane. After cell lysates were incubated with each primary antibody overnight at 4°C, Protein A/G Plus was added and complexes incubated for 4 h at 4°C. Pellets were washed, and immunoprecipitated complexes were released and analyzed by western blotting using phosphospecific antibodies. Semi-quantitative analysis was performed using densitometry, and results are expressed as activity relative to that of the control. Results are shown as means ± SD of three independent experiments. Statistical significance was determined using the Student’s *t*-test compared with the control. Asterisks indicate statistically significant difference between 5, 15, 30 min treatments and the control (*p < 0.01). For nucleolin, statistical significance was additionally determined using one-way ANOVA compared with the 5 min activated level. Sharps indicate the statistically significant difference between 5 min treatment and after 15 min (#*p* < 0.01).

To verify the changes observed in the phosphoproteome profile induced by sevoflurane, SH-SY5y cells were exposed to sevoflurane and a similar ether anesthetic agent, isoflurane, in a time-dependent manner. Nucleolin; the Ras-GAP SH3 binding protein, HSP90β; and matrin 3 were immunoprecipitated using their respective antibodies, and detected with an anti-phospho tyrosine/serine/threonine antibody. As shown in Fig 3, phosphorylation of the nucleolin protein in SH-SY5y cells increased following treatment with sevoflurane and isoflurane (*showing statistically significant differences compared to the control, *p* < 0.01), and then decreased significantly (^#^showing statistically significant differences compared to the levels of the 5 min treatment, *p* < 0.01). Phosphorylation of the HSP90β protein in SH-SY5y cells also increased following exposure to sevoflurane and isoflurane (*showing statistically significant differences after 15 min compared to the control, *p* < 0.01). The phosphorylation of the Ras-GAP SH3 binding protein and matrin 3 increased markedly after 15 min following treatment with sevoflurane and isoflurane (*showing statistically significant differences compared to the control, *p* < 0.01). The results of our immunoprecipitation experiments are in agreement with those obtained from proteome profiling.

### Effect of anesthetics on actin polymerization and microfilament growth

Proteins that are known to be implicated in cytoskeleton regulation, including the Rho GTPase, Ras-GAP SH3 binding protein, actin-related protein, and actin, were differentially phosphorylated. Therefore, we examined the intracellular distribution of actin microfilaments using antibodies that recognize microfilament F-actin and monomeric G-actin, in order to determine how the phosphorylation status, induced by anesthetic stimulation, affects microfilament organization in neuronal cultures.

**Fig 4:**
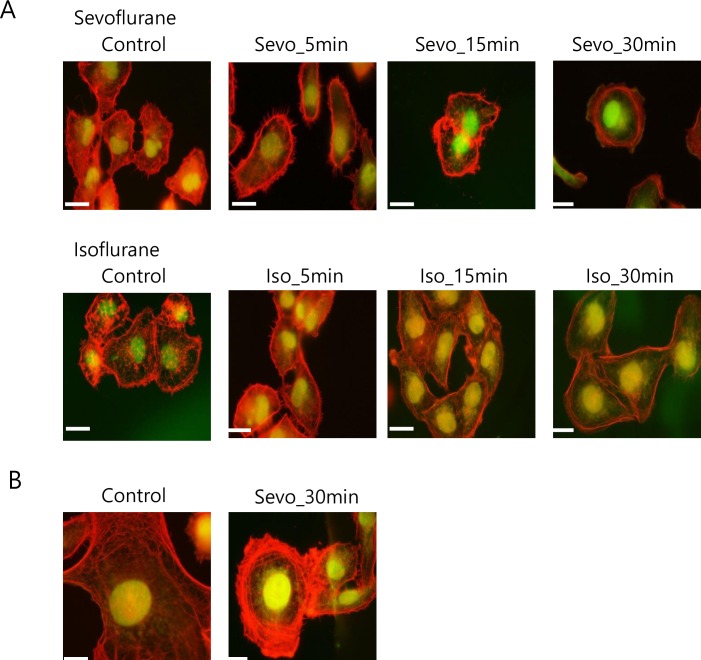
Reorganization of the microfilament cytoskeleton in anesthetic-treated SH-SY5y cells. Cells were cultured on glass coverslips and incubated for the indicated times with vehicle or 1 mM sevoflurane and isoflurane. The redistribution of filamentous actin and globular actin was determined by phalloidin–halloidinne aDNAse I-Alexa488 staining and immunofluorescence microscopy. A. Magnification, 400 × (the scale bar represents 50 μm); B. Magnification, 1,000 × (the scale bar represents 20 μm). Similar results were obtained in three independent experiments.

Without anesthetics, F-actin in neuronal cells was found in thickly diffuse and meshed structures, corresponding to microfilaments throughout the cytosolic region of the cell body and in the filopodia of membrane ruffles, rather than being localized in the cortical region (Fig 4). Upon treatment with 1 mM sevoflurane and isoflurane for 5, 15, and 30 min, the characteristic diffuse and meshed F-actin staining gradually decreased and was replaced by marked peripheral distribution (Fig 4). Subsequently, we examined the effects of anesthetics on the ratio of F-actin to G-actin, given that actin filaments are linear polymers of globular actin subunits. F-actin staining was abundantly observed in dendritic spines in the absence of anesthetics; however, the degree of staining in peripheral dendritic spines seemed to decrease upon treatment with anesthetics.

**Fig 5:**
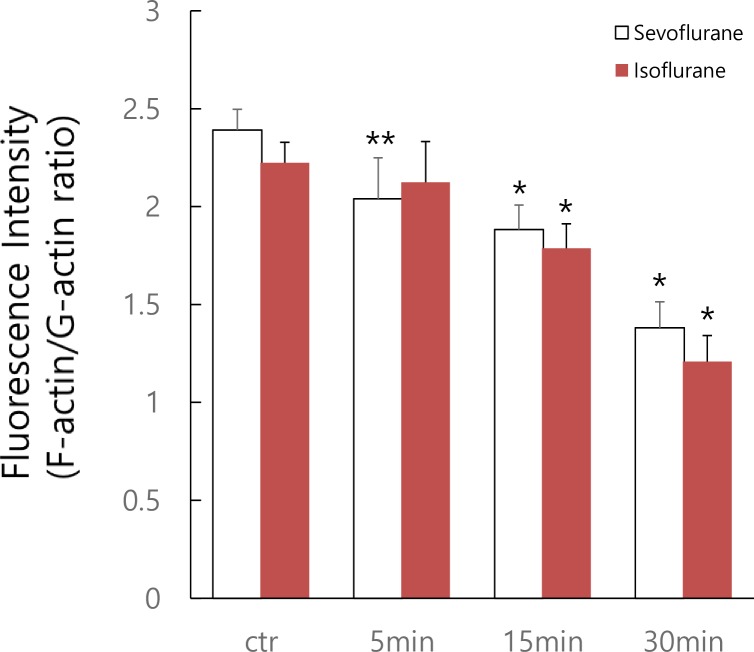
Quantification of fluorescence intensity of F-actin and G-actin. Quantification of fluorescence intensity was performed from 25 randomized districts on culture plates from each treatment group (control, 5, 15, and 30 min). Quantification was performed for four independent experiments. Results were expressed as F-actin activities relative to the one of G-actin and statistical analysis was performed. Results are shown as means ± SD of 100 randomized districts obtained from four independent experiments. Statistical significance was determined using the Student’s *t*-test compared with the control. Asterisks indicate statistically significant difference between 5, 15, 30 min treatments and control (*p < 0.01, **p < 0.05).

We next quantified the fluorescence intensities of F-actin and G-actin in 25 randomized districts on culture glass for each treatment group (control, 5, 15, and 30 min). The calculation of the ratio of F-actin to G-actin at different time intervals of anesthetic treatment revealed that the ratio decreased in a time-dependent manner, indicating that anesthetics cause dissolution of actin filaments (Fig 5).

### Involvement of group 1 PAKs signaling in the effect of anesthetics on actin depolymerization

**Fig 6:**
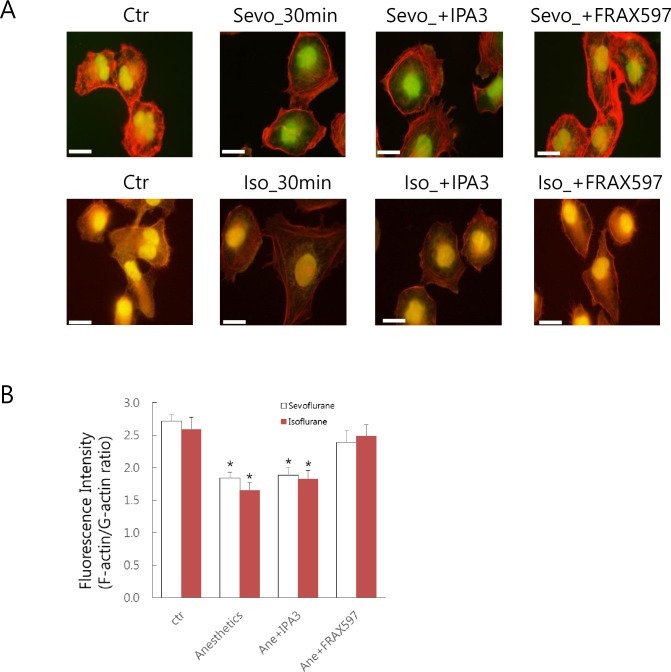
PAK-dependent rearrangement of actin filaments induced by sevoflurane and isoflurane. (A) Cells were cultured on glass coverslips and pretreated with IPA3 (5 μM) or FRAX597 (50 nM) for 30 min and subsequently exposed to 1 mM sevoflurane or isoflurane for 30 min. The redistribution of filamentous actin and globular actin was determined by phalloidin–Alexa568 and DNAse I-Alexa488 staining and immunofluorescence microscopy. Magnification, 400 × (the scale bar represents 50 μm). Similar results were obtained in three independent experiments. (B) Quantification of fluorescence intensity was performed in 25 randomized districts from each treatment group. Quantification was performed for four independent experiments. Results were expressed as F-actin activity relative to that of G-actin and statistical analysis was performed. Results are shown as means ± SD of 100 randomized districts obtained from four independent experiments. Statistical significance was determined using the Student’s t-test compared with the untreated control. Asterisks indicate statistically significant differences between treatment and control (*p < 0.01).

Recently, p21-activated kinases (PAKs), a family of serine/threonine kinases, have been identified to directly phosphorylate actin and be involved in peripheral or cortical actin reorganization and depolymerization of fibers leading to the formation of filopodia [19–21]. Therefore, we investigated the effect of PAK inhibitors on anesthetic-induced cytoskeletal rearrangements. Pre-incubation of cells with the specific PAK1 inhibitor IPA3 (5 μM) did not abolish the characteristic decrease in diffuse and meshed F-actin staining caused by anesthetics, showing statistically significant differences between treatment and untreated control (*p* < 0.01). However, pre-incubation of cells with 50 nM FRAX597, an inhibitor of group 1 PAKs (PAK1, 2, and 3) abolished peripheral distribution of F-actin and a decrease in the ratio of F-actin to G-actin induced by anesthetics, as shown in Fig 6, confirming the involvement of PAK2 or PAK3 in anesthetic-induced actin rearrangement signaling.

## Discussion

The present study provides time-dependent phosphoproteomic data following exposure of the neuronal cell line SH-SY5y to sevoflurane. This is the first investigation on the anesthetic-responsive phosphoproteome and its dynamic changes in a time course.

Analyzing the identified kinetic pattern of phosphorylation, proteins implicated in cytoskeletal regulation appeared to be phosphorylated in sevoflurane-treated cells. The Rho GTPase, one of these proteins, is known to play essential roles in the regulation of microfilament dynamics accompanied by morphogenetic changes such as cytokinesis, vesicle trafficking, cell migration, and axonal guidance [22]. The Rho GTPase has recently been found to be implicated in the plasticity of dendritic spines in the adult brain as well [23]. Another protein that was phosphorylated in sevoflurane-treated cells and considered a related marker for cytoskeletal dynamics is the Ras-GAP SH3 binding protein, which has been shown to interact with RasGAP *via* its SH3 domain and to play a role in cell migration and adhesion [24, 25]. Finally, proteins that act as key regulators of cytoskeletal dynamics include the Rho GTPase activating protein, actin-related protein, and actin. The Rho GTPase activating protein interacts with members of the Rho family of small GTPases, which have been shown to be involved in signaling for the dynamic formation of actin filaments in the cytoskeleton, such as lamellipodia and filopodia [26–28]. Microtubule and microfilament dynamics have been shown to be important in the maintenance of neuronal morphology and function [29–40]. In particular, changes in dendritic microtubule dynamics were suggested to be important determinants for NMDA-dependent long-term depression [29]. In our phosphoproteome profiling, the Rho GTPase activating protein and Rho GTPase were increasingly phosphorylated (by a few-fold) until 2 min, and returned to their minimum levels between 15 and 30 min of treatment. Subsequently, the phosphorylation levels of the Ras-GAP SH3 binding protein reached a maximum of 1000-fold that of the control within 15 min, and subsequently returned to their minimum within 30 min. In addition, phosphorylation of actin cytoplasmic 2 was upregulated to more than 1000-fold that of the control after sevoflurane exposure, followed by a saturation trend. The sequential phosphorylation behavior of the Rho GTPase activating protein, Rho GTPase, Ras-GAP SH3 binding protein, and actin proteins observed upon sevoflurane exposure conforms to existing knowledge regarding signaling processes involved in actin rearrangement.

Based on the cited literature, together with our results showing increased phosphorylation of the Ras-GAP SH3 binding protein, the Rho GTPase activating protein, Rho GTPase, and actin-related protein, it is plausible that immediate changes in the phosphorylation status of these proteins might be intimately related with anesthetic-induced neuronal events, such as channel gating at inhibitory or excitatory synapses. When anesthetic agents cause decreased memory, learning, and immobility *via* the inhibition of AMPA and NMDA receptors activating glycine receptors and depressing spinal cord functions, their mechanism of action might involve changes in the structure of synaptic connections through rearrangement of the actin cytoskeleton, which is regulated by Rho GTPases.

A previous work has suggested that exposure of cells to opioids results in modifications of the actin cytoskeleton through a PAK1-dependent mechanism [21, 41]; we further investigated actin depolymerization in cells due to anesthetics, using an inhibitor of PAKs. Experiments using an inhibitor of Group 1 PAKs did not reveal the presence of accumulated peripheral distribution of F-actin nor a reduction in the ratio of F-actin to G-actin, suggesting that the observed microfilament redistribution was a consequence of the involvement of these PAKs in actin phosphorylation. As shown in Fig 6, PAK1-specific inhibitors failed to nullify the observed effects of anesthetics, and a decrease in the ratio of F-actin to G-actin was observed, indicating that the activation of PAK2 or PAK3, rather than PAK1, is associated with the consequent actin phosphorylation in the early stages of actin repolymerization induced by the anesthetics sevoflurane and isoflurane.

RNA processing proteins, such as the heterogeneous nuclear ribonucleoprotein C (C1/C2), splicing factor 3A, serine/arginine-rich splicing factor 9, nucleolin, ErbB3 binding protein 1, and heterogeneous nuclear ribonucleoprotein H, were also phosphorylated transiently in response to sevoflurane exposure in SH-SY5y cells. Nucleolin and matrin 3 are nuclear matrix proteins and function in RNA processing [42, 43]. HSP90β, which was also transiently phosphorylated in SH-SY5y cells upon treatment with sevoflurane and isoflurane, is an abundant molecular chaperone. HSP90 is known to affect nucleolin levels by modulating its phosphorylation by CDK1 to increase mRNA stability during mitosis [44]. The heterogeneous nuclear ribonucleoprotein family plays a role in processes of RNA biogenesis, such as pre-mRNA splicing [45]. The ErbB3 binding protein 1 binds to RNA targets and acts as a downstream effector of RNA processing [46].

Based on these previous observations and our present results, we believe that anesthetic-stimulated events at the cellular level cause changes in the splicing machinery through phosphorylation, which may be linked to their related functionality.

In the present report, we suggest that phenotypic changes in microfilaments could be caused by the anesthetics sevoflurane and isoflurane through phosphorylation dynamic behaviors, which might be a general mechanism that underlies anesthetic-induced neuronal events, leading to a decrease in memory, learning, and immobility.

## Supporting Information

S1 File Compilation of comparative proteome datasets. (DOC)

S2 File Proteomics raw data. (ZIP)
