# Supplementary material for: Phosphoproteome Profiling of SH-SY5y Neuroblastoma Cells Treated with Anesthetics: Sevoflurane and Isoflurane Affect the Phosphorylation of Proteins Involved in Cytoskeletal Regulation
Source: PLoS One. 2016 Sep 9;11(9):e0162214. doi: 10.1371/journal.pone.0162214 (PMC5017685; doi:10.1371/journal.pone.0162214)

| **1. Analyses** |
| --- |

| **2-DE Gel** | **No of sample** | **Sample origin** | **Protein staining** | **Gel type** |
| --- | --- | --- | --- | --- |
| 2-DE image acquisition | 5 | Human | ProQ Diamond  Colloidal CBB | 12cm small |

| **2. Condition** |
| --- |

| **2-DE Gel** | **IEF(1^st^)** | **Slab gel(2^nd^)** | **Image acquisition** | **Image analysis** |
| --- | --- | --- | --- | --- |
| 5 gels | 4-10 NL IPG | 12% step gradient  8.5x7cm | 16bit TIFF | PDQuest |

| **3. Results** |
| --- |

1. **2-DE image** : scanned original images were attached with TIFF files separately.

* pI and MW as follows


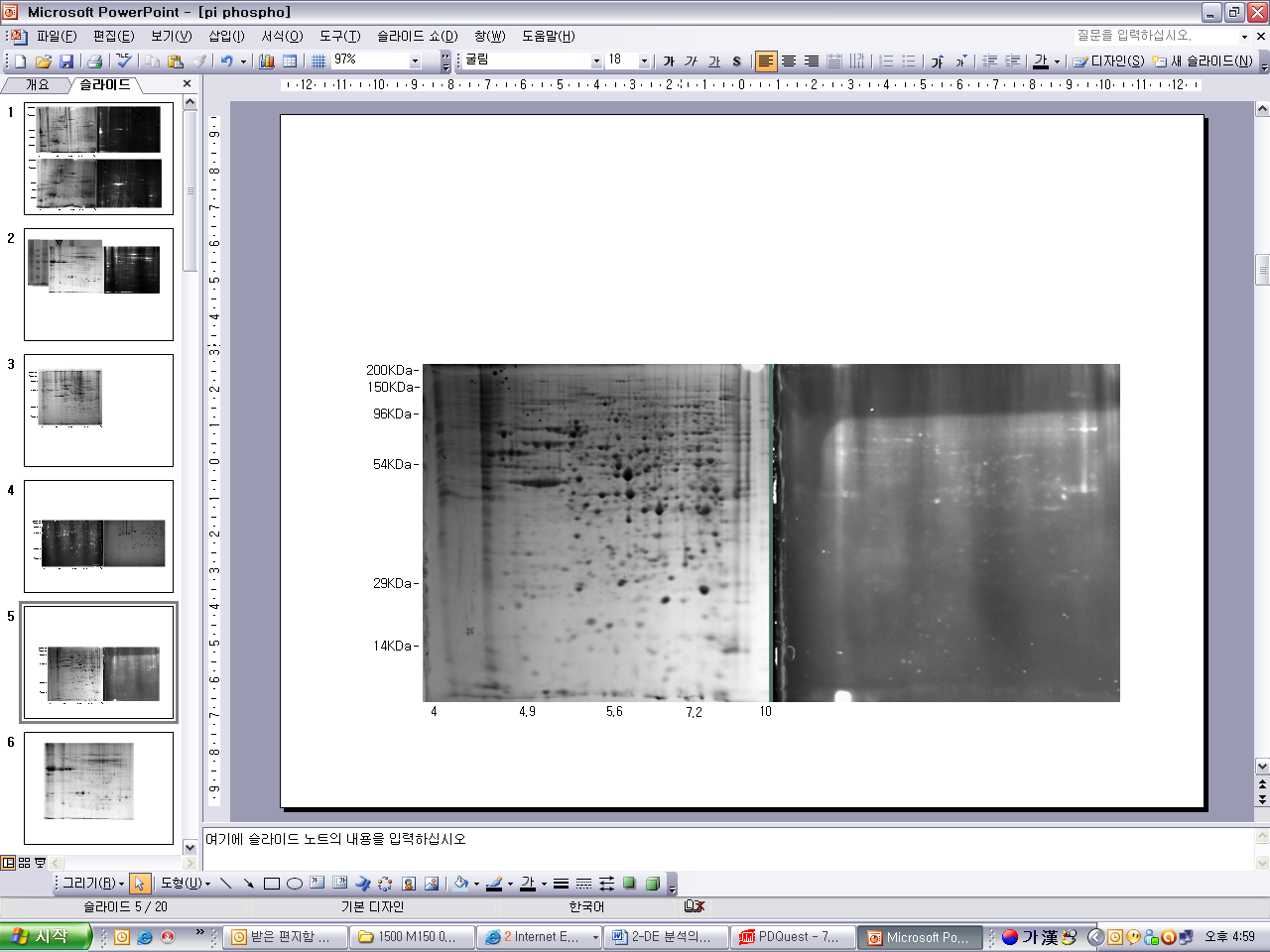


1. **2-DE sample Match set- full images (match set )**

**
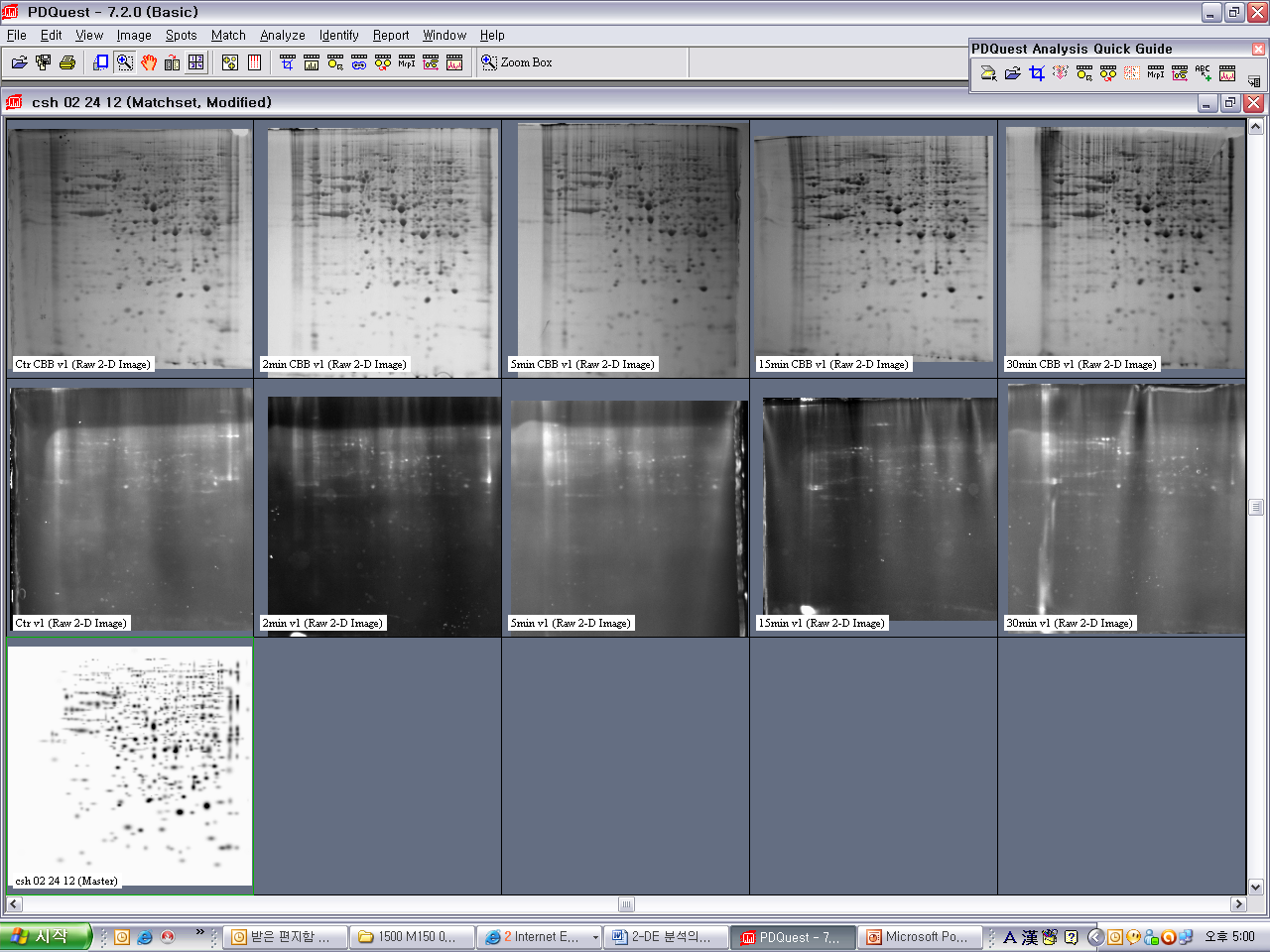
**

*** approximate sample spot number**

**ctr : 629 CBB staining (68 proQ staining)**

- **2min : 688 (85)**
- **5min : 582 (75)**
- **15min : 707 (75)**
- **30min : 766 (86)**

**after the gel was stained with ProQ Diamond and scanned using photographic equipment with a Cy3 emission filter, the proteins were stained with Coomassie Brilliant Blue G-250 and scanned again. Spots on the phosphoimage and the CBB image were compared to the same coordinate, which was represented on the Master image (the lowest of the following captured images; it is a virtual integrated image of our data series, to handle the coordinates).**

1. **2-DE sample Match set partial image**

: cut gel into 6 pieces

①

②

③

⑥

⑤

④


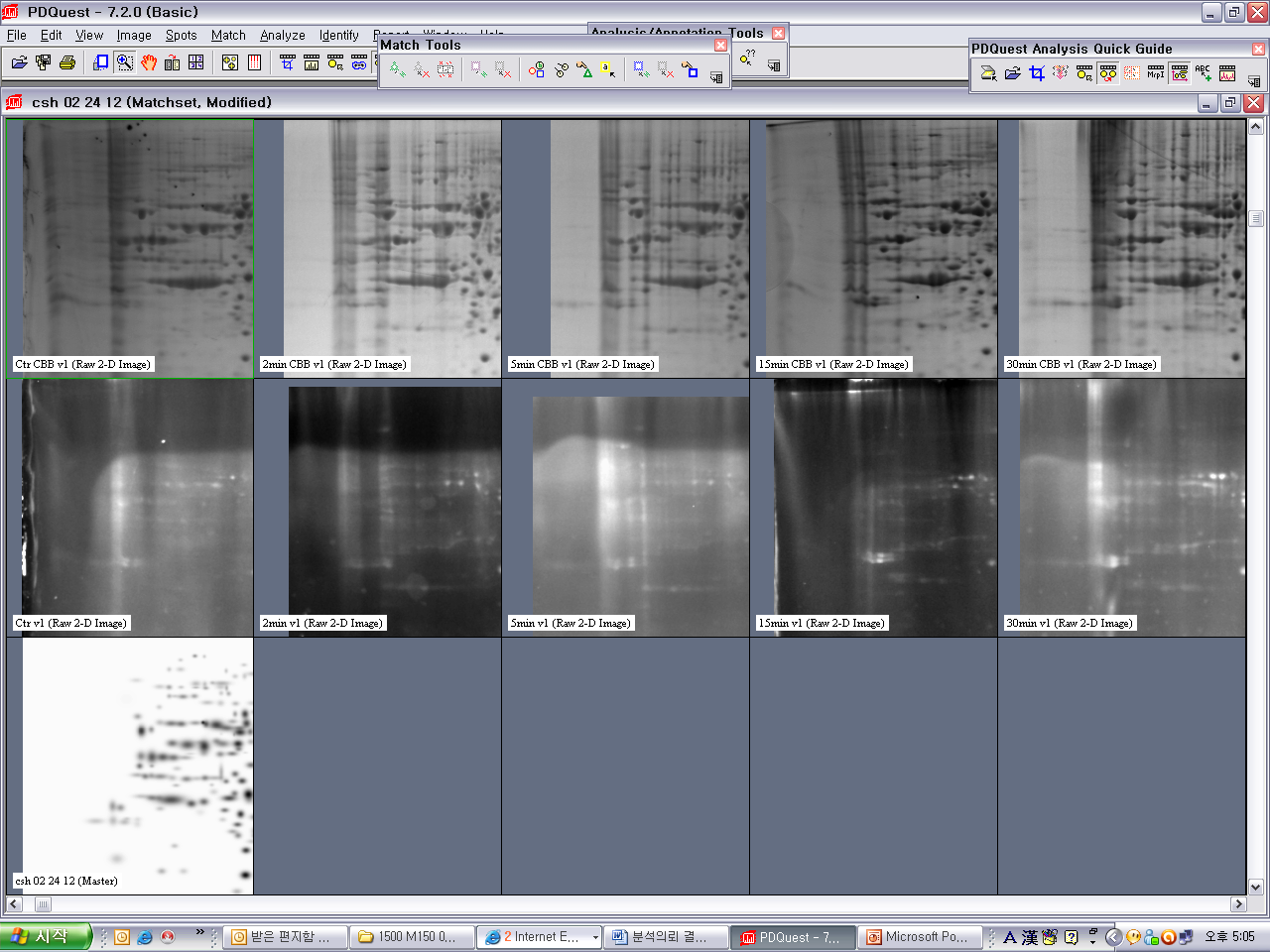


②


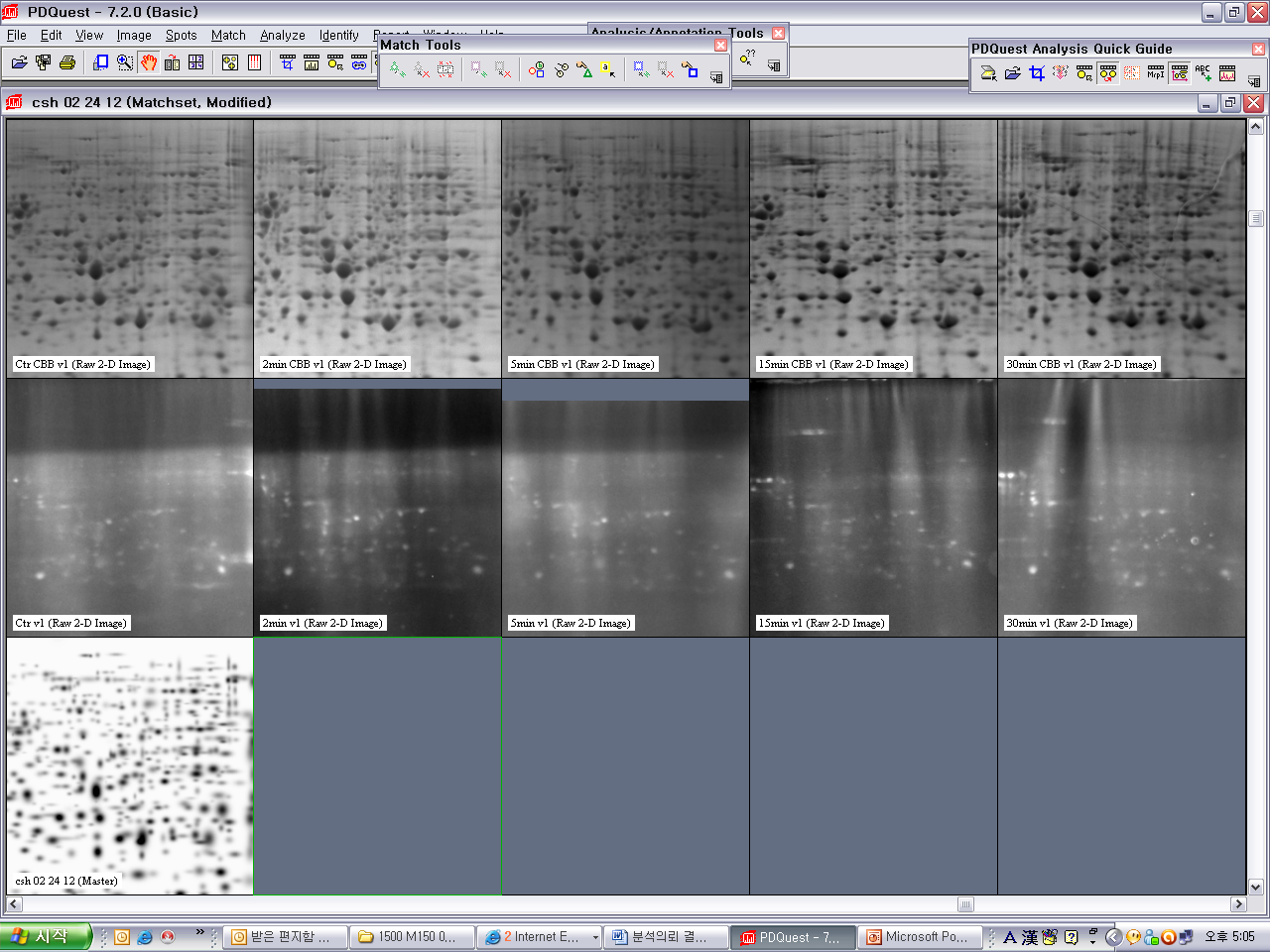


③


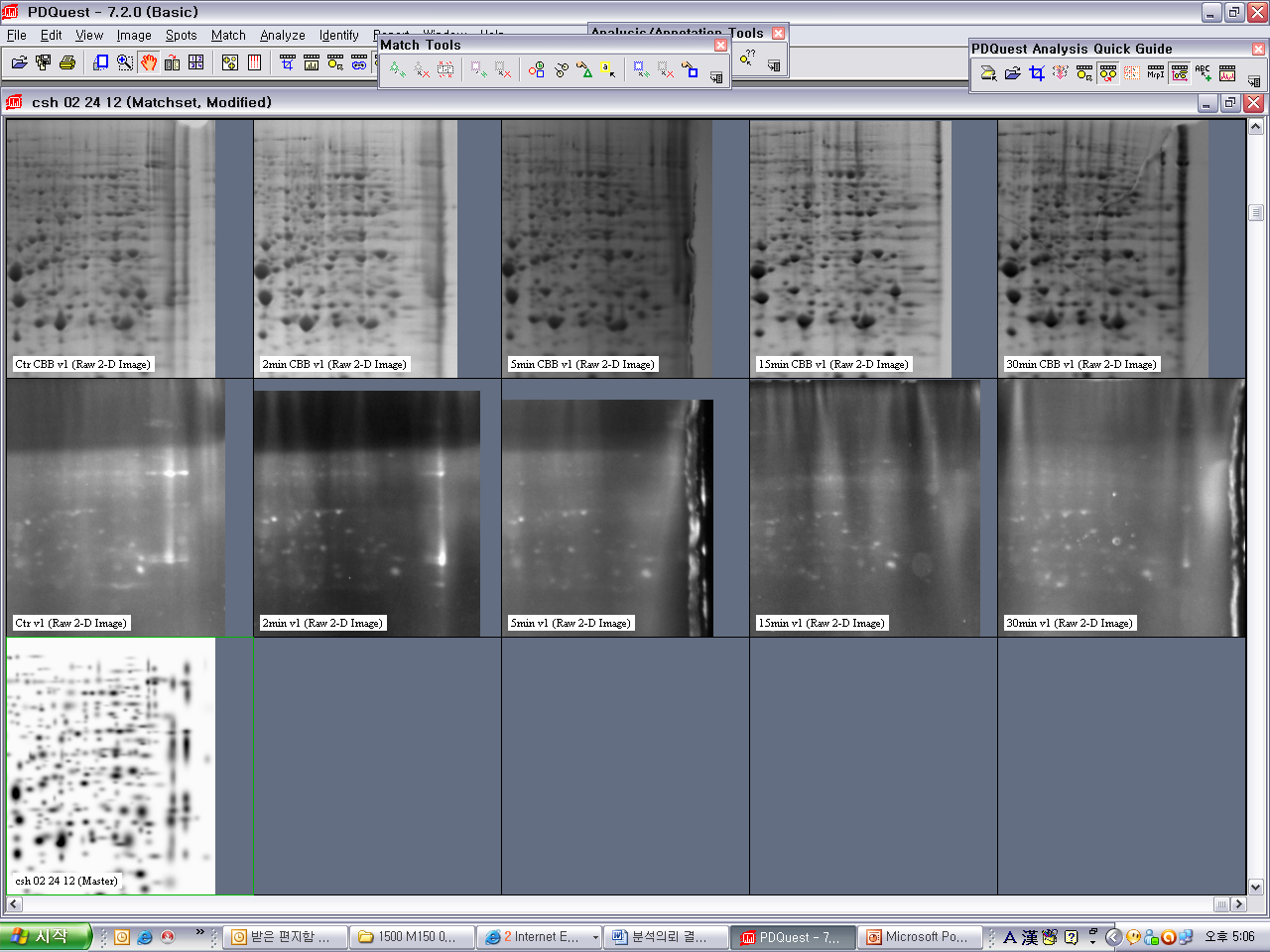


④


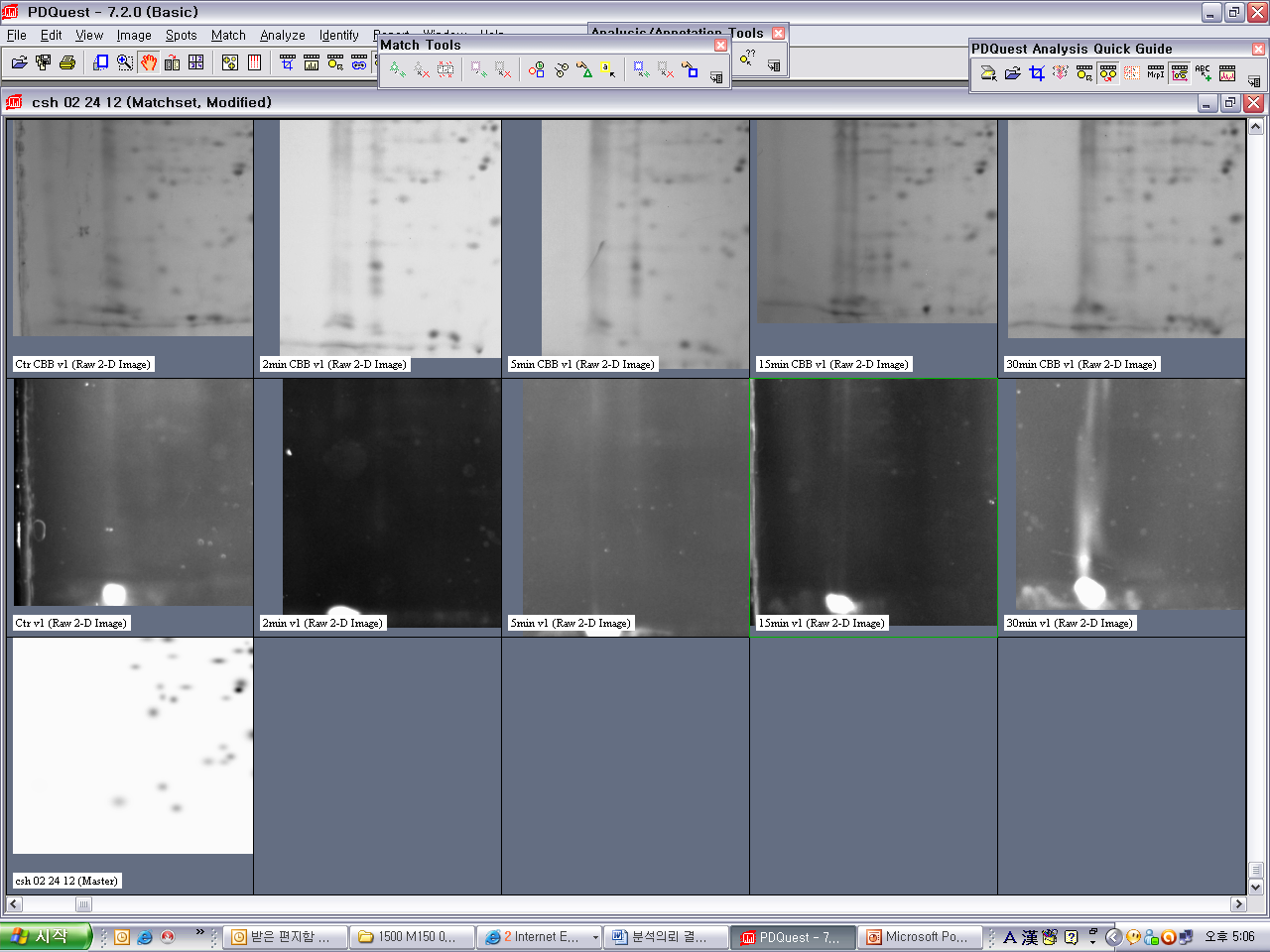


⑤


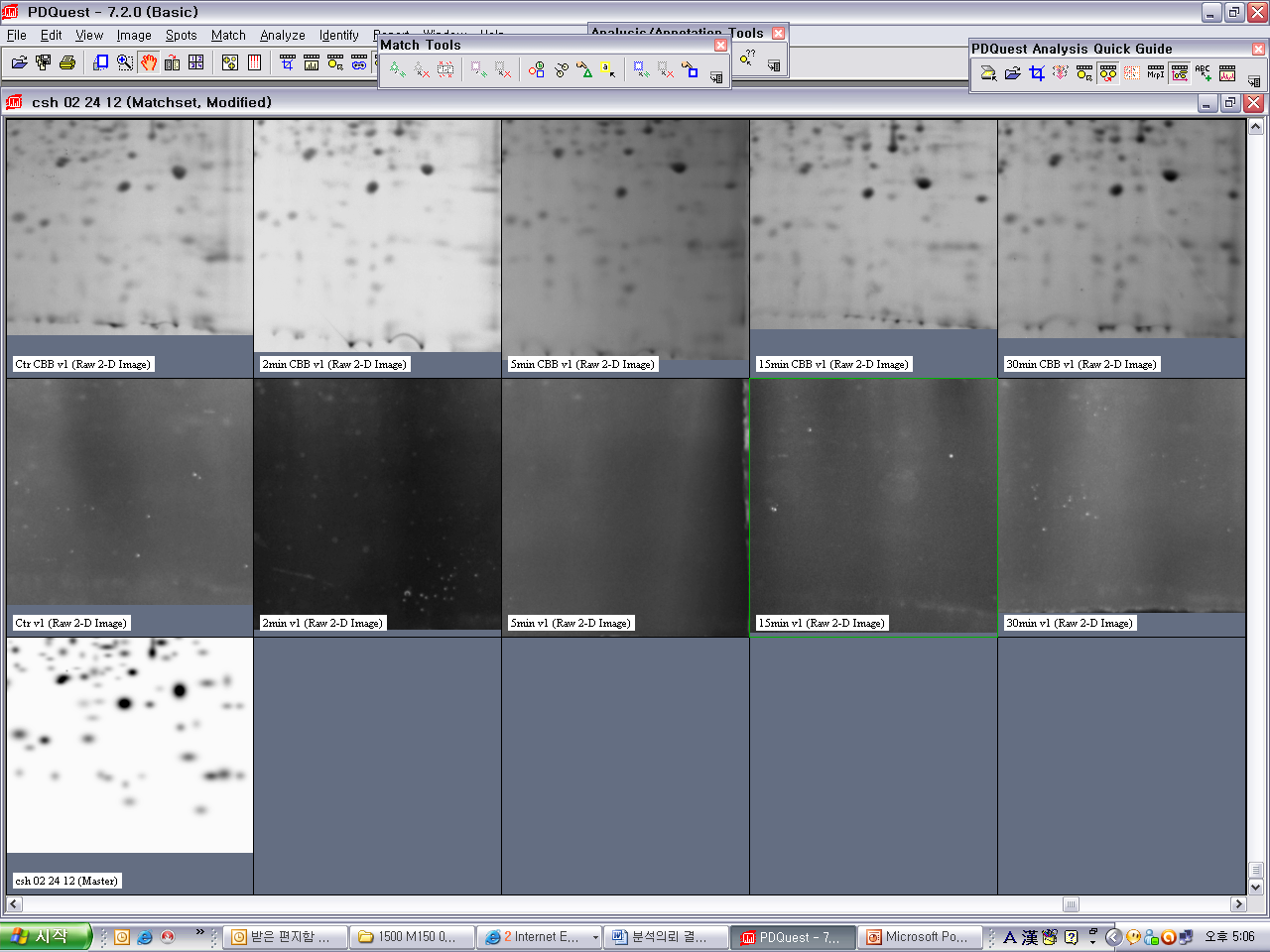


⑥


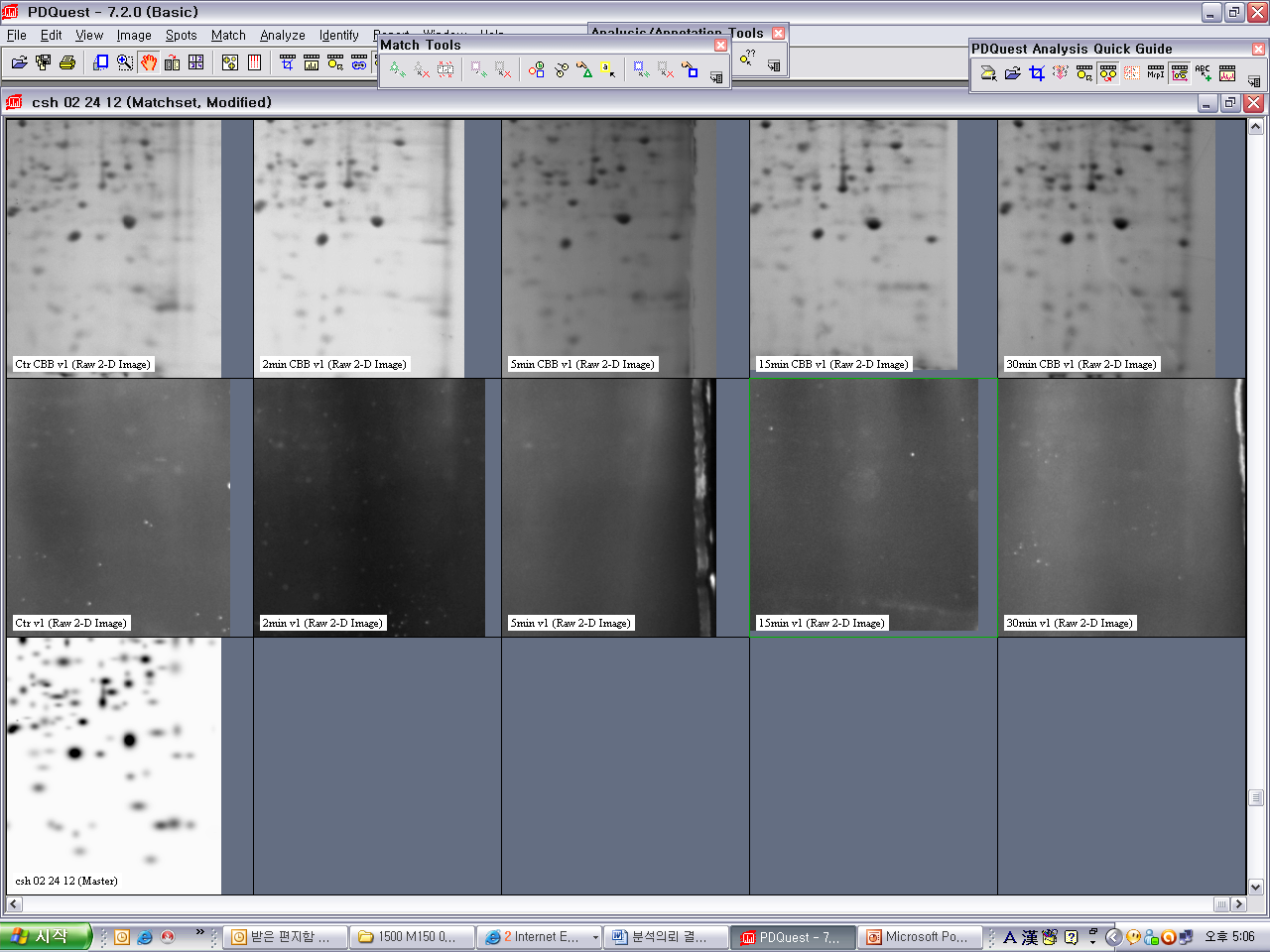


1. **selected spot image**


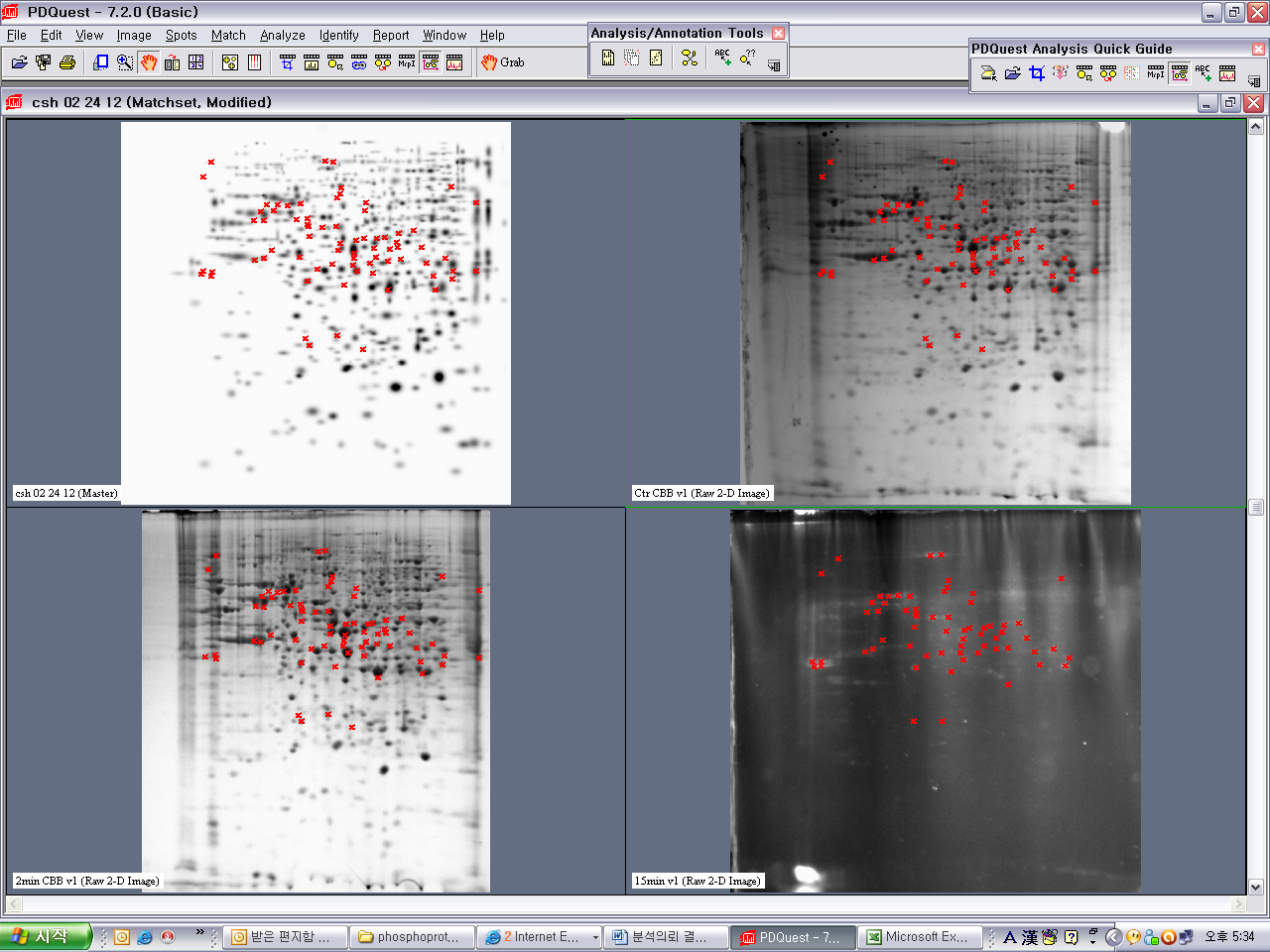


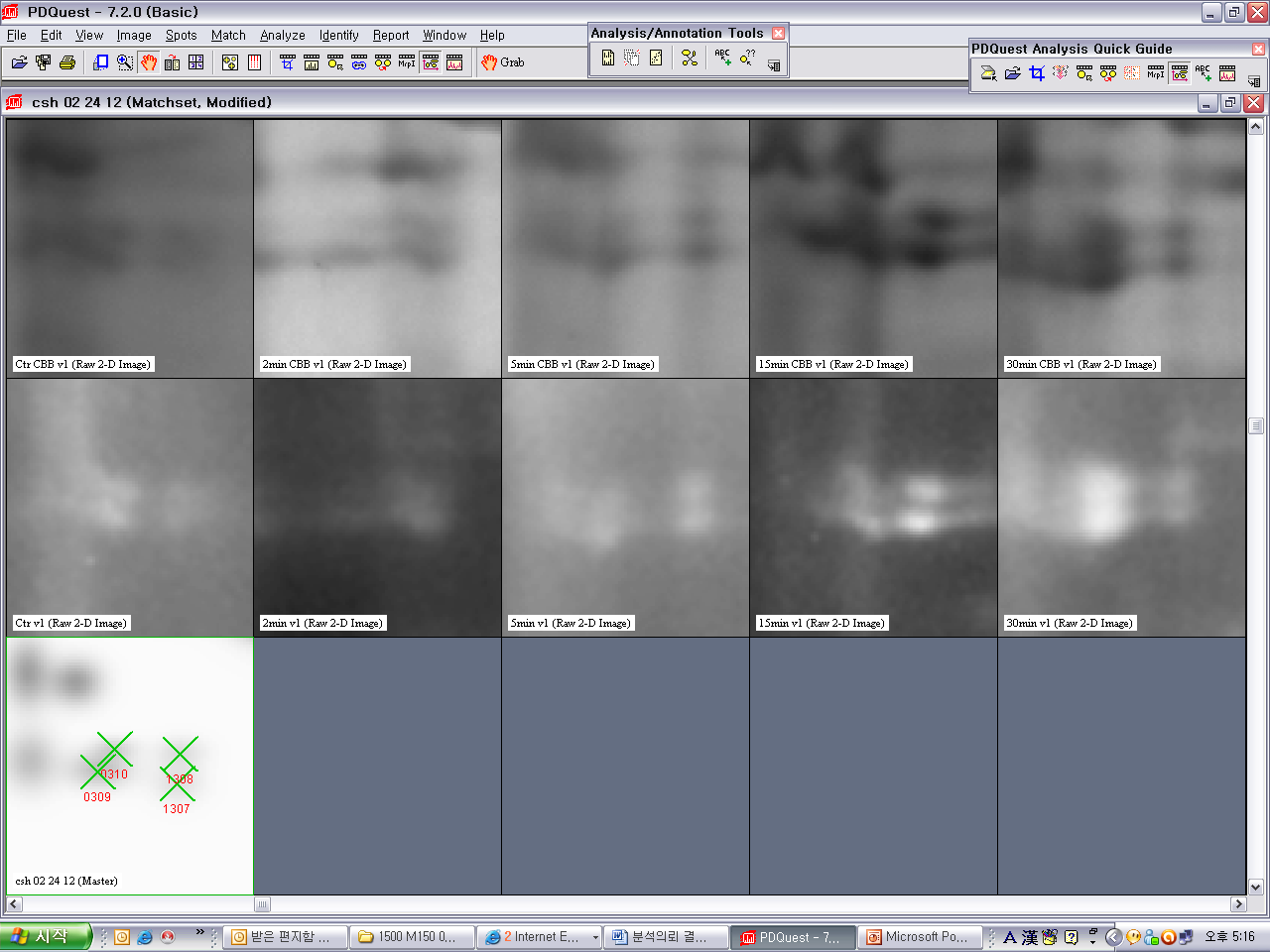


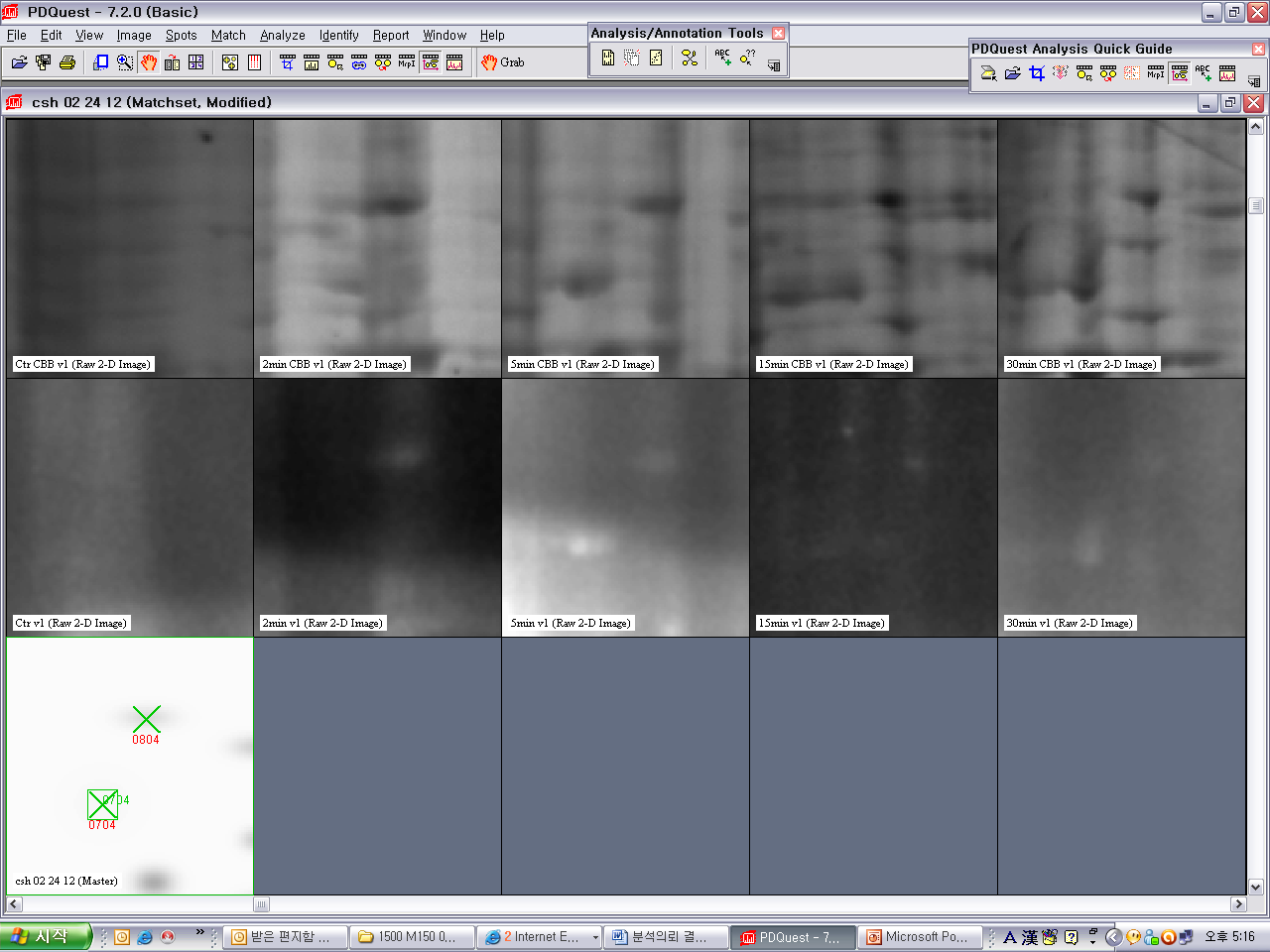


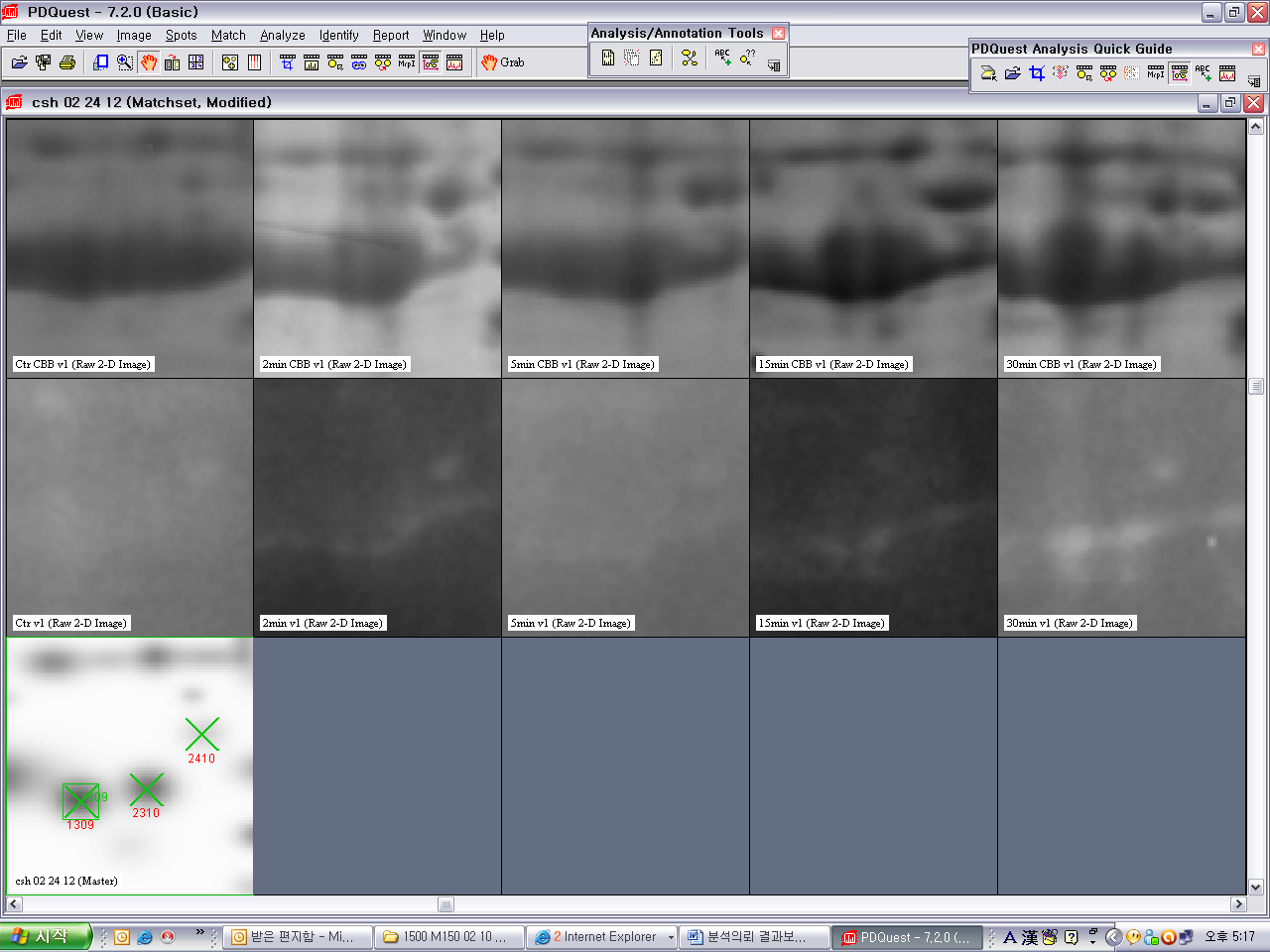


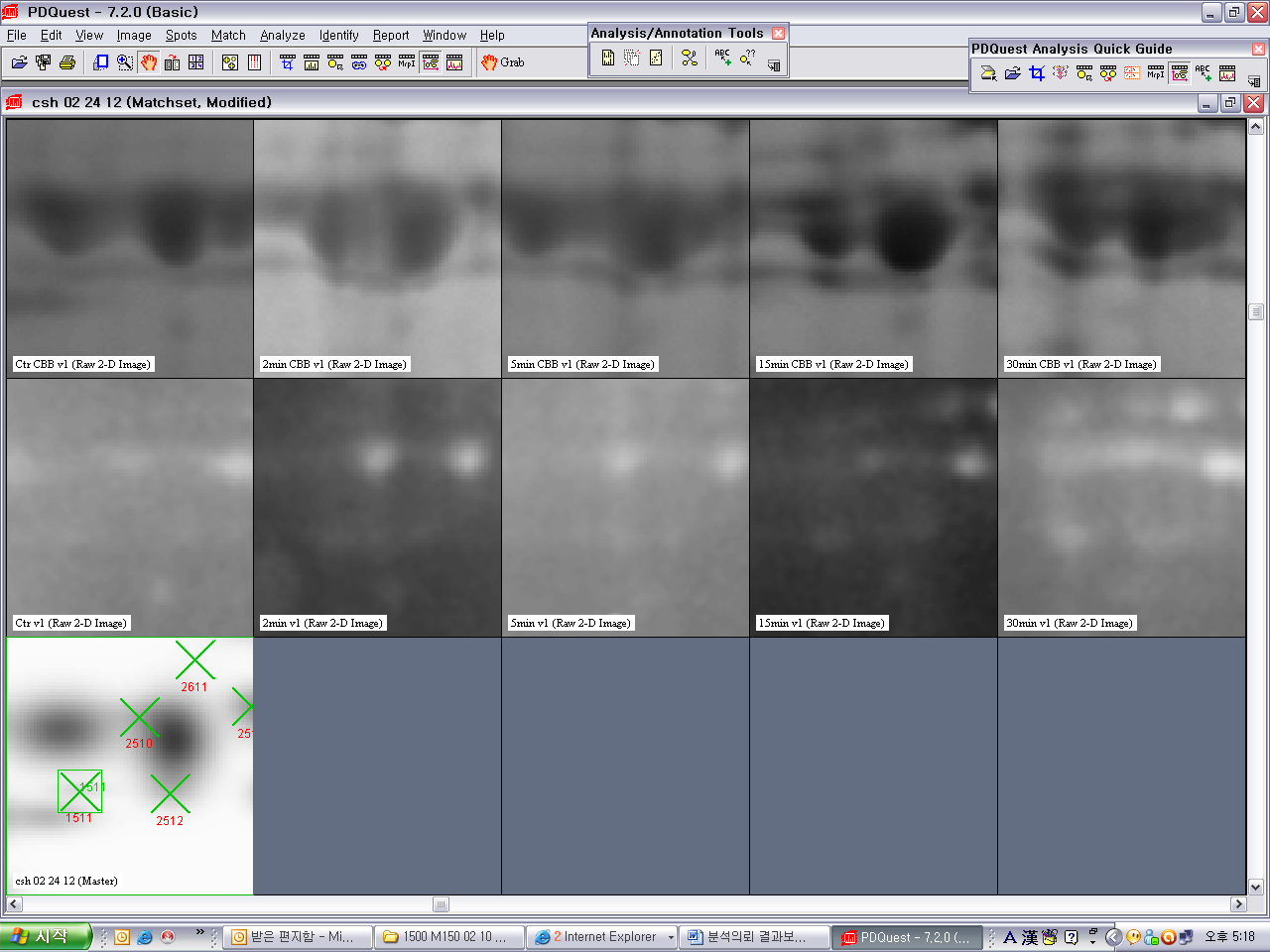


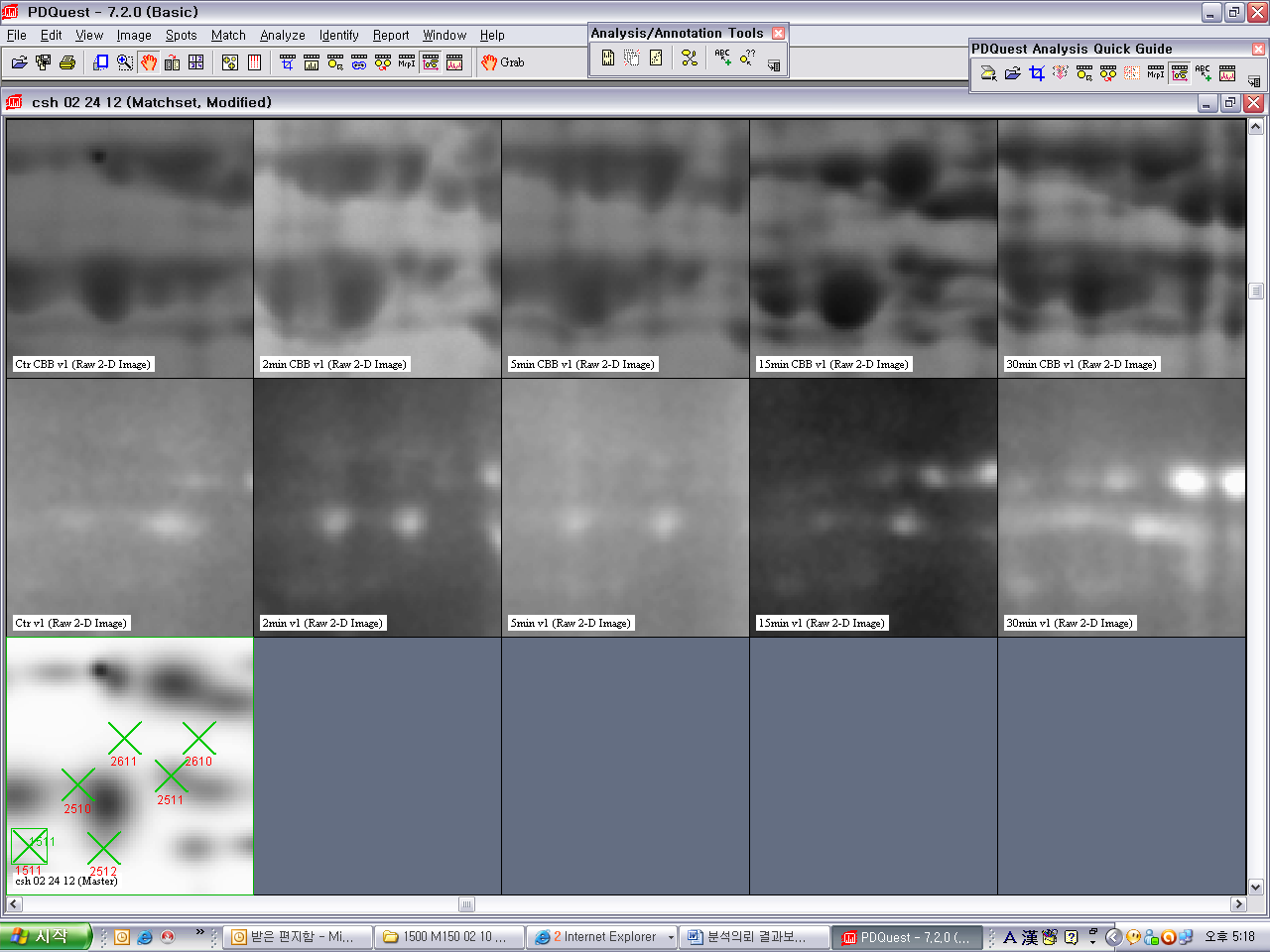


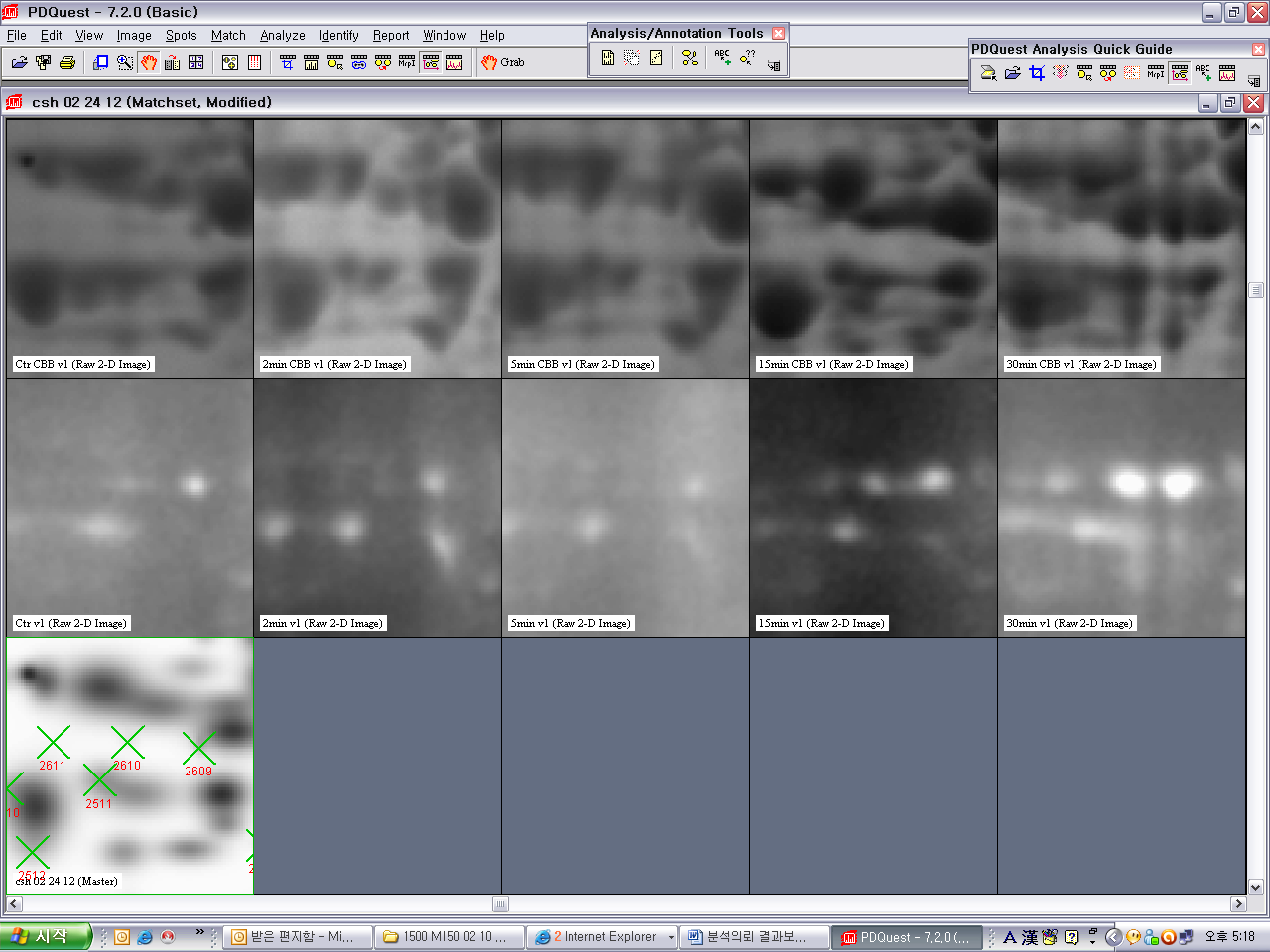


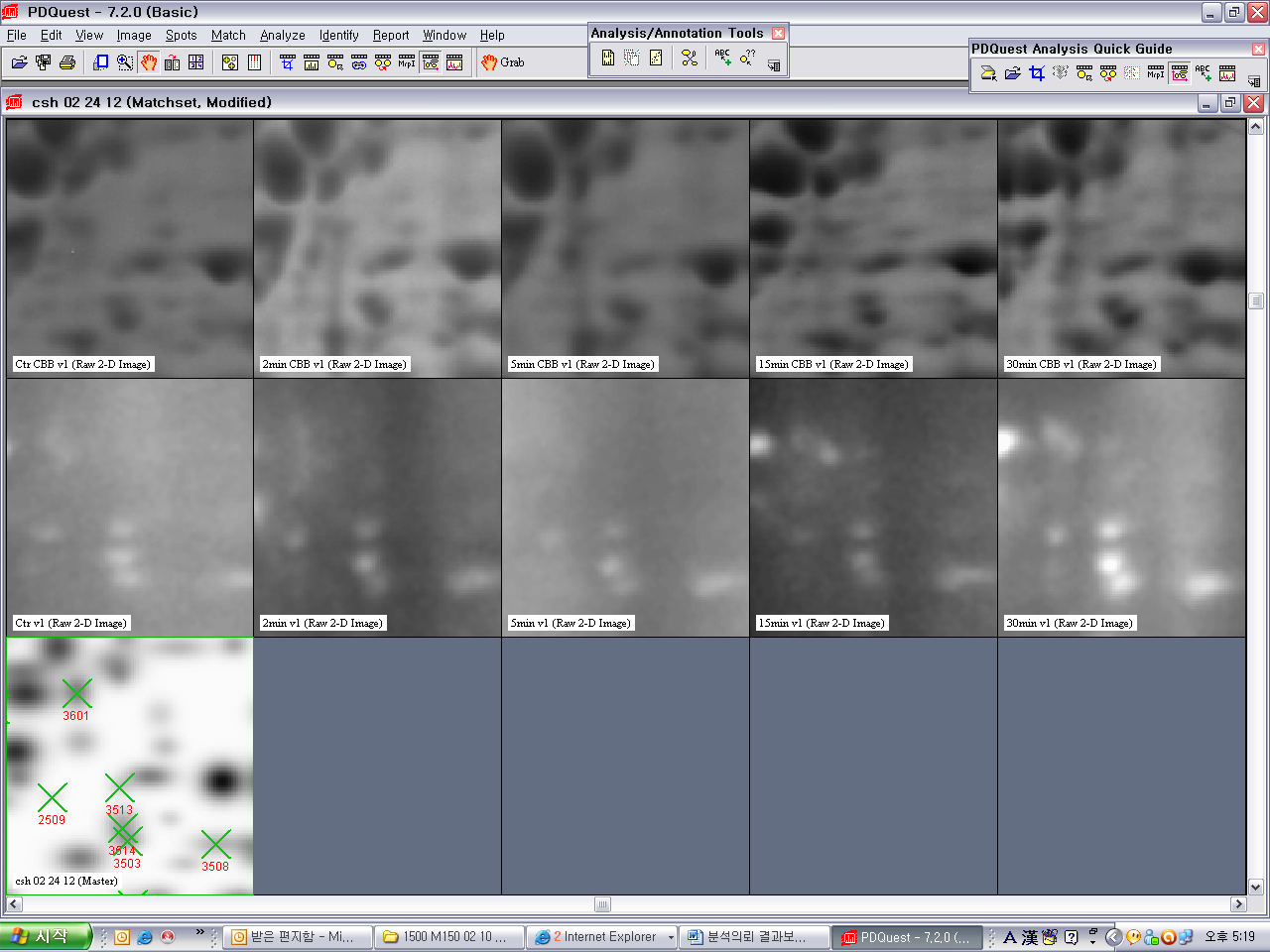


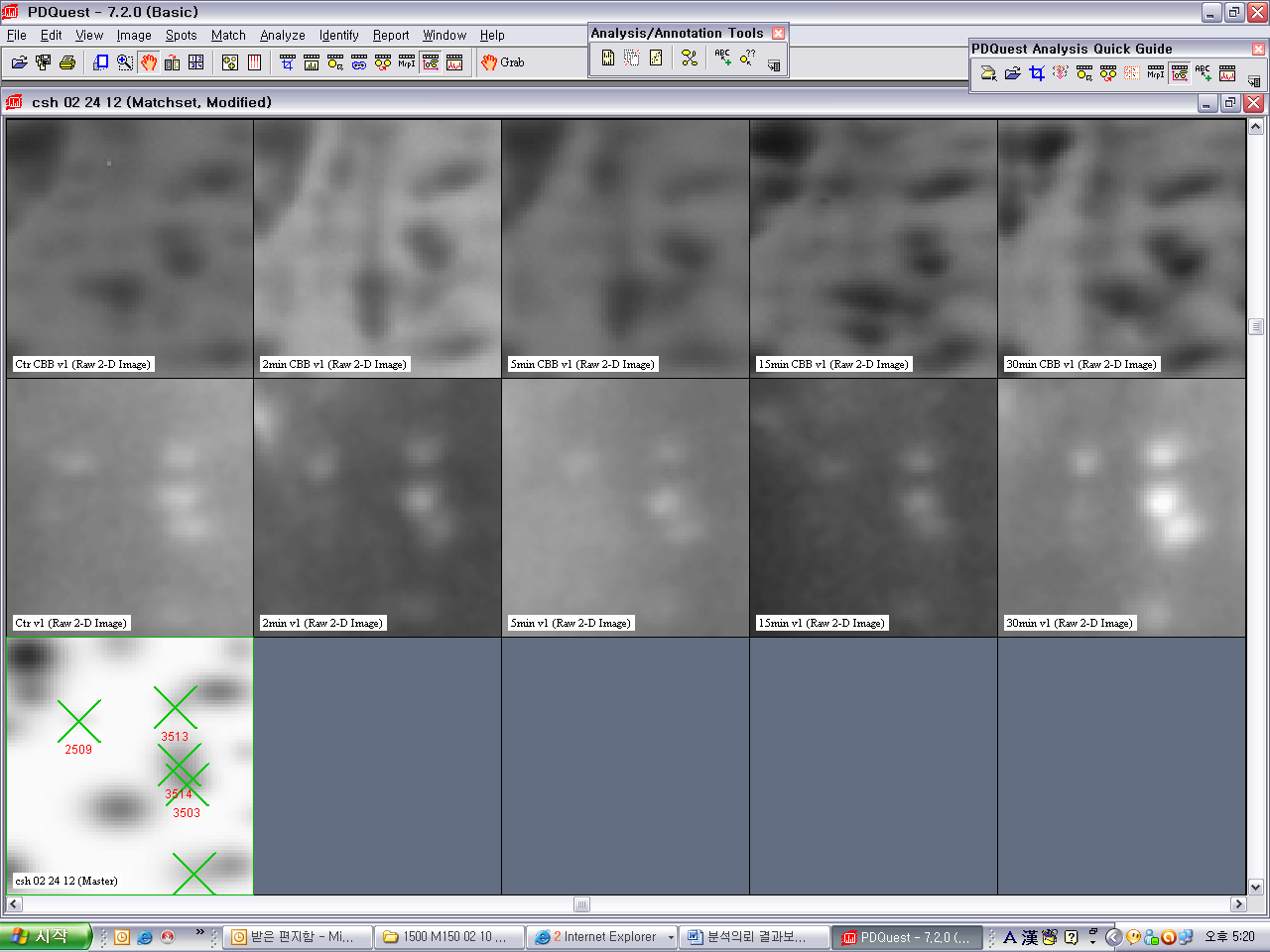


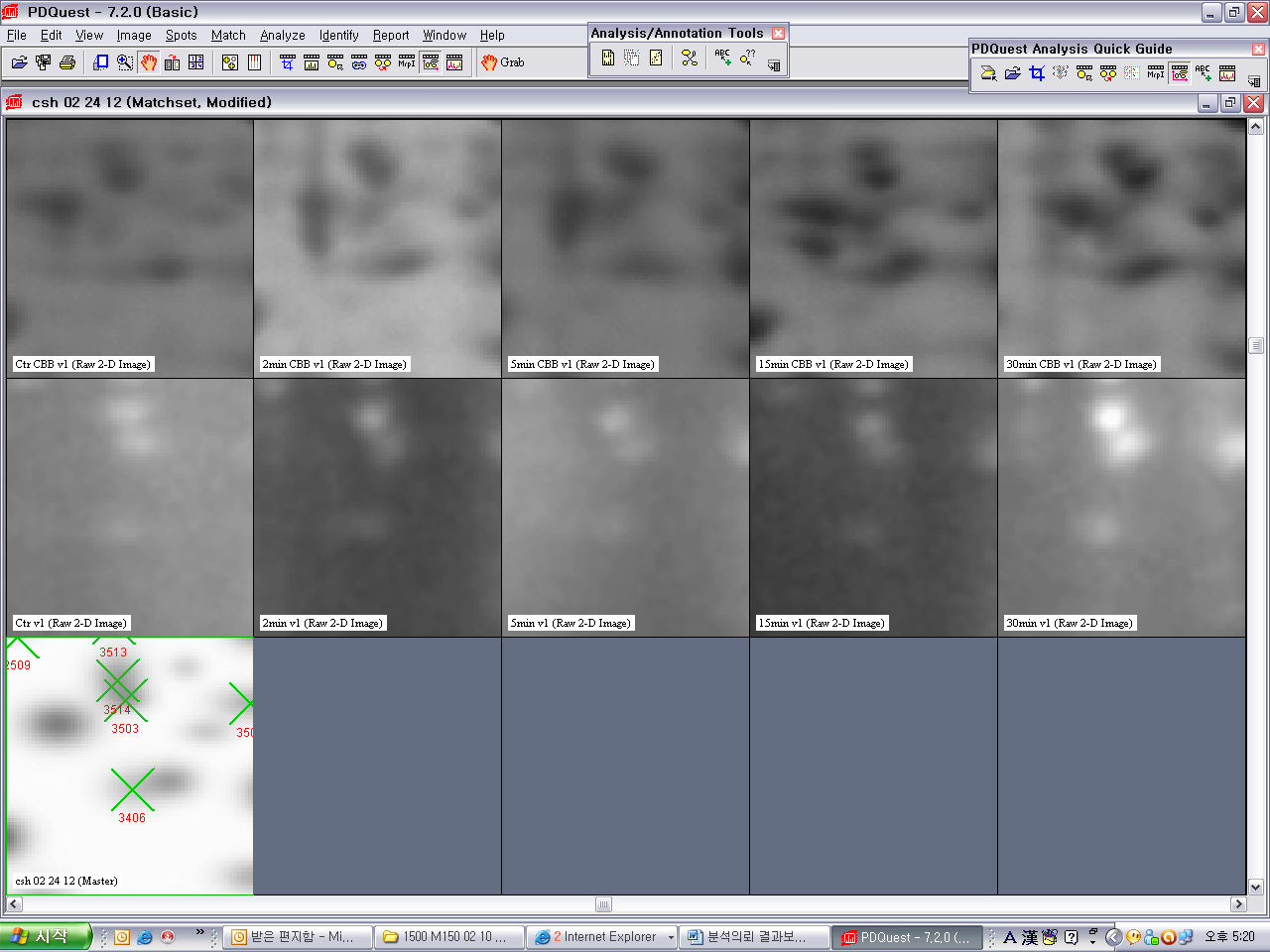


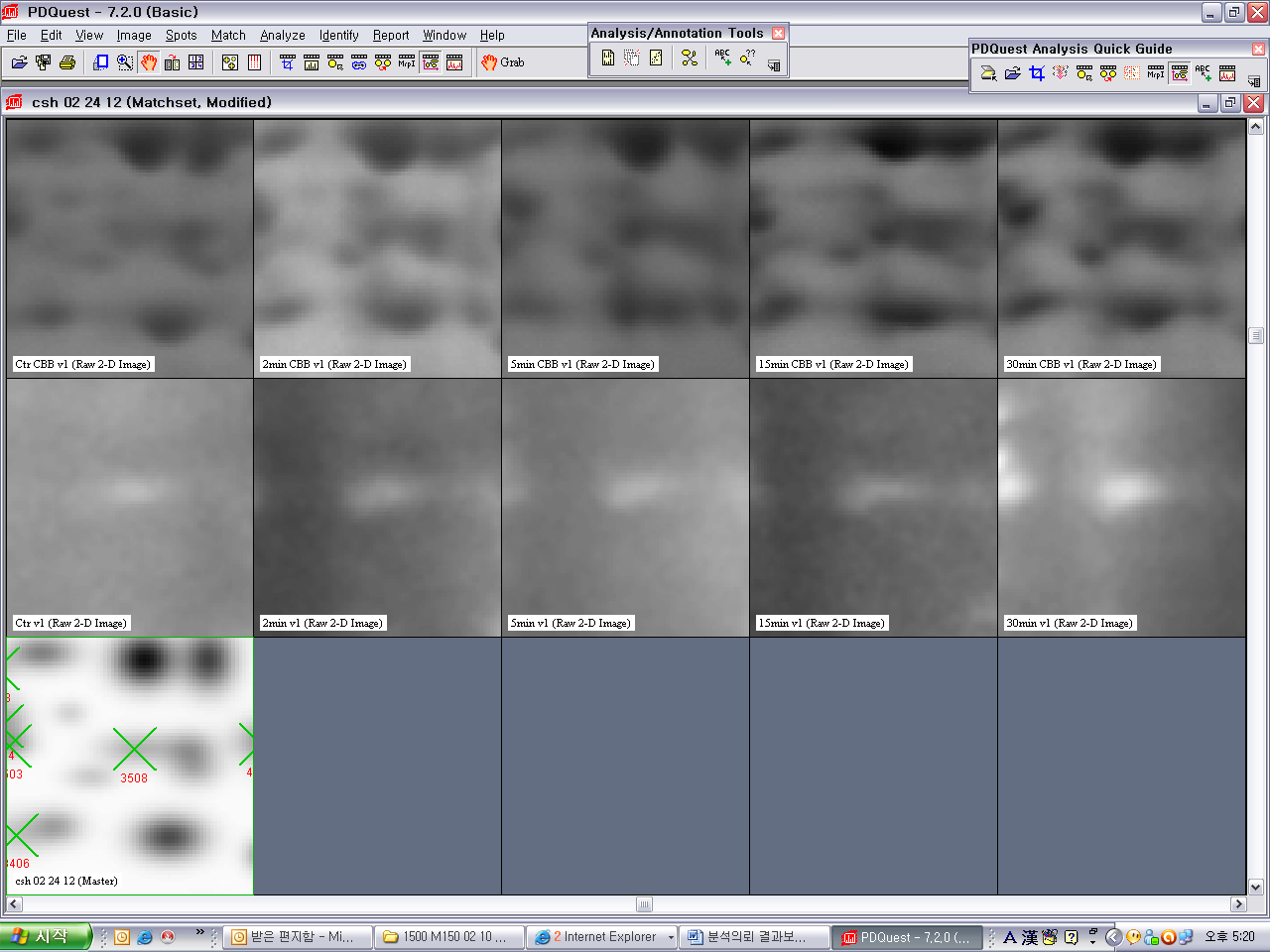


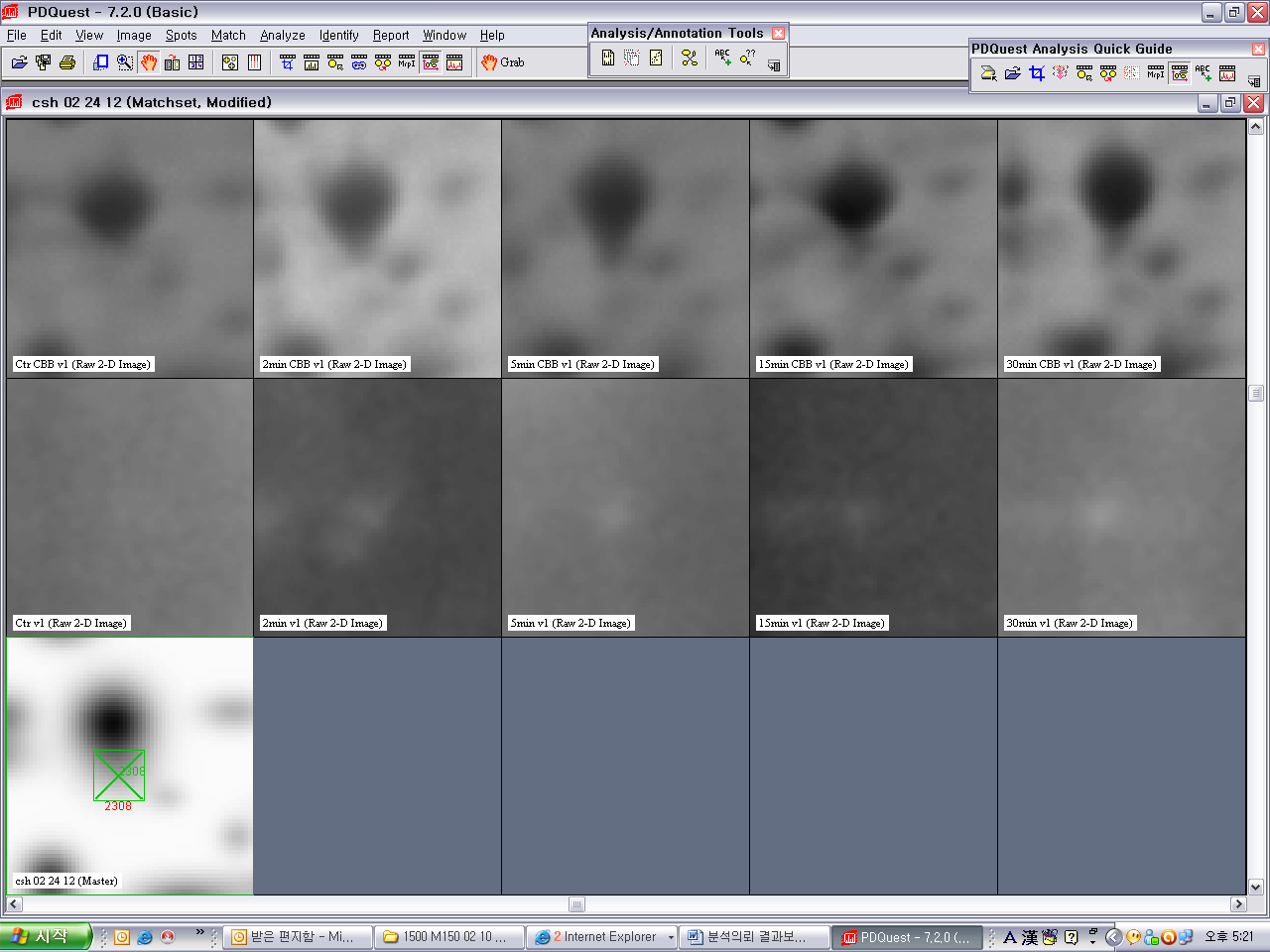


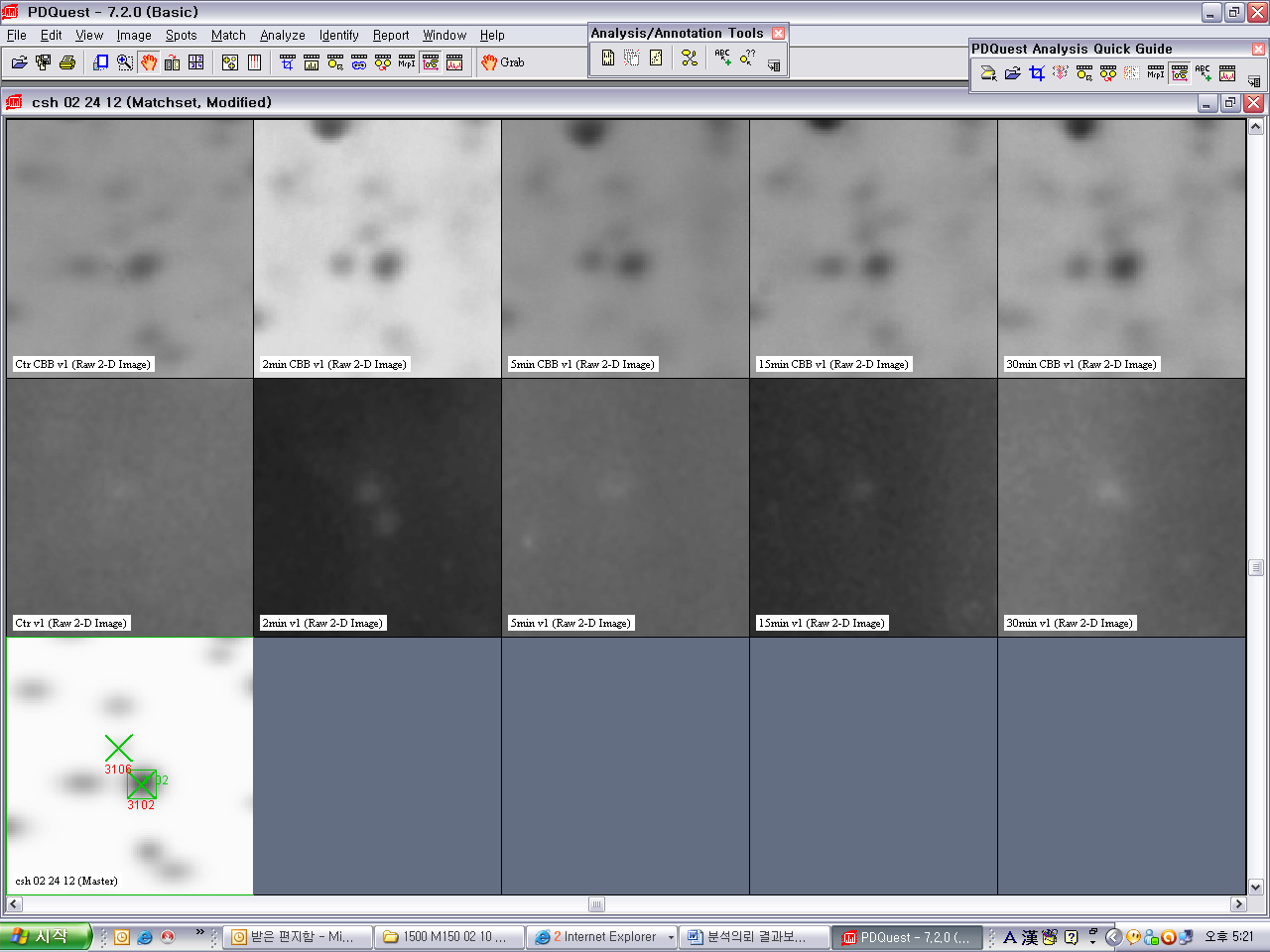


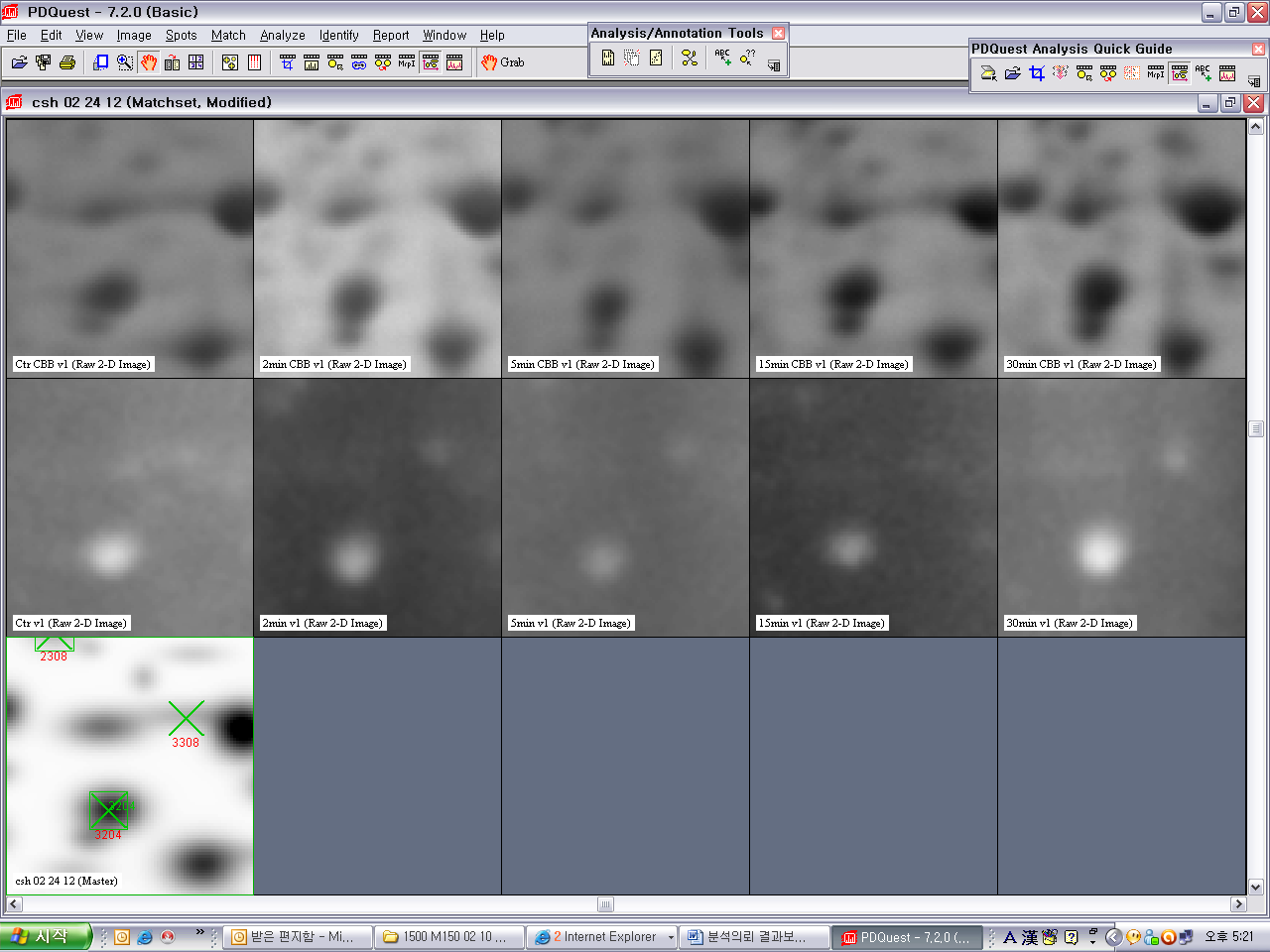


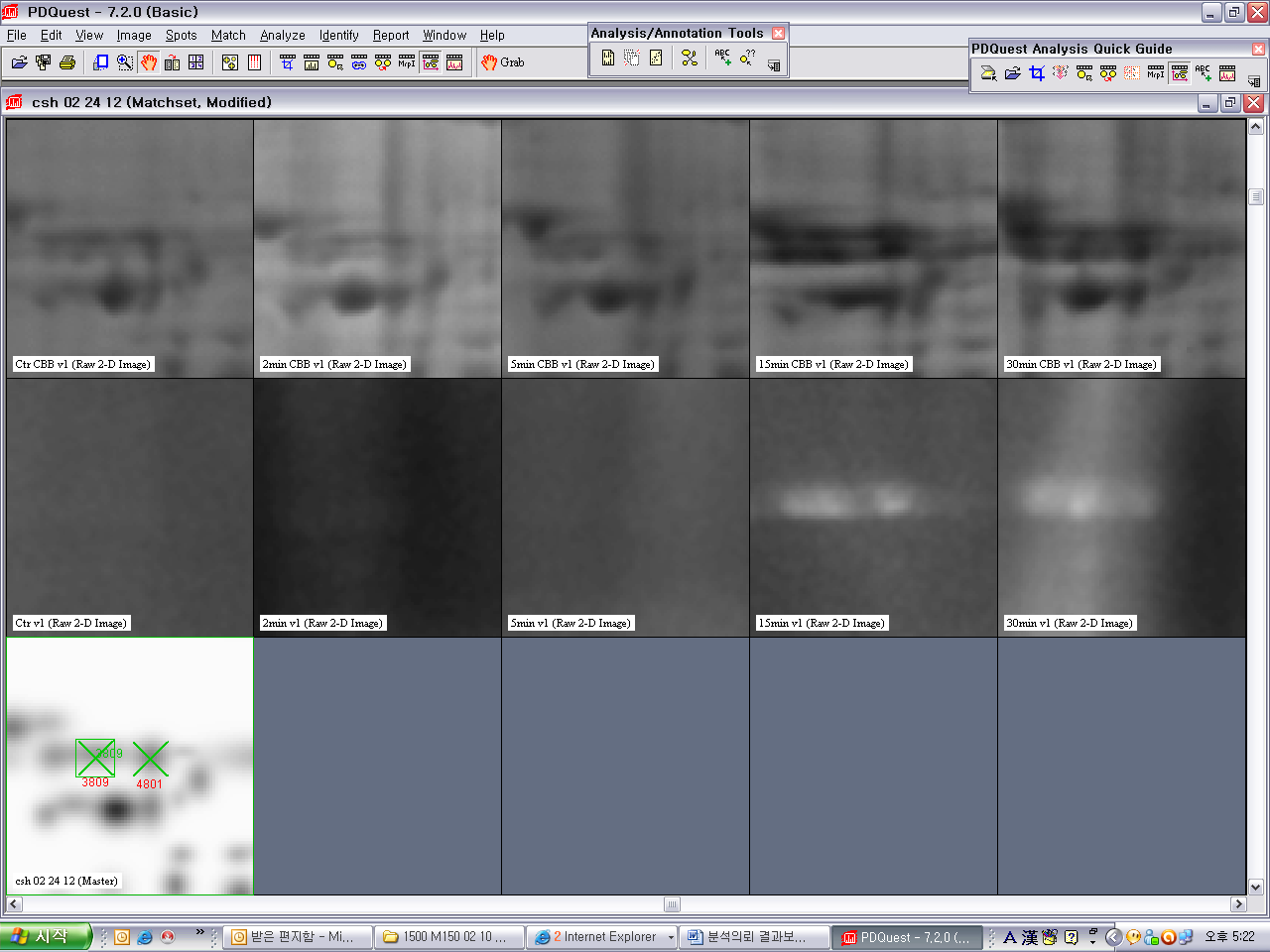


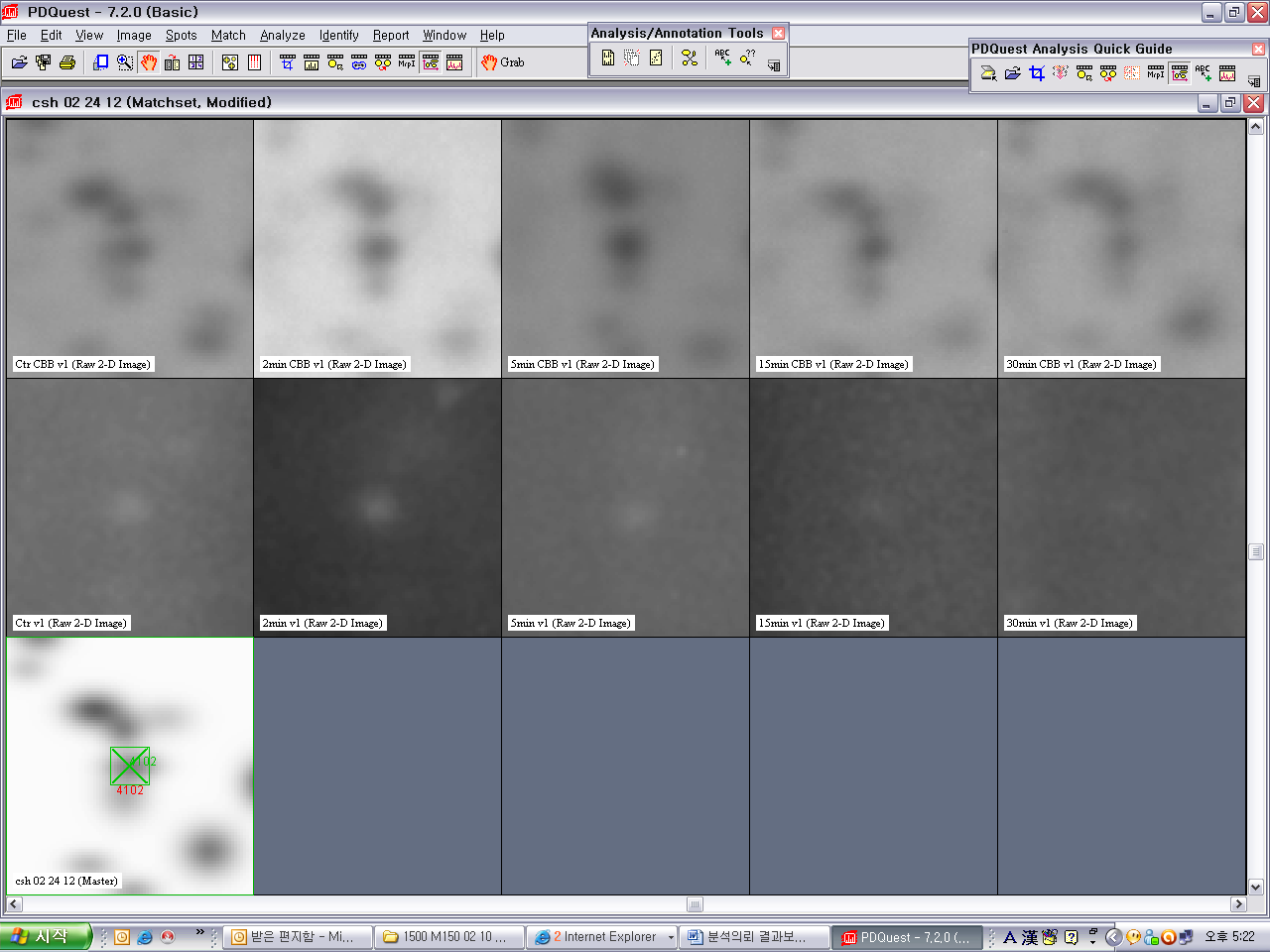


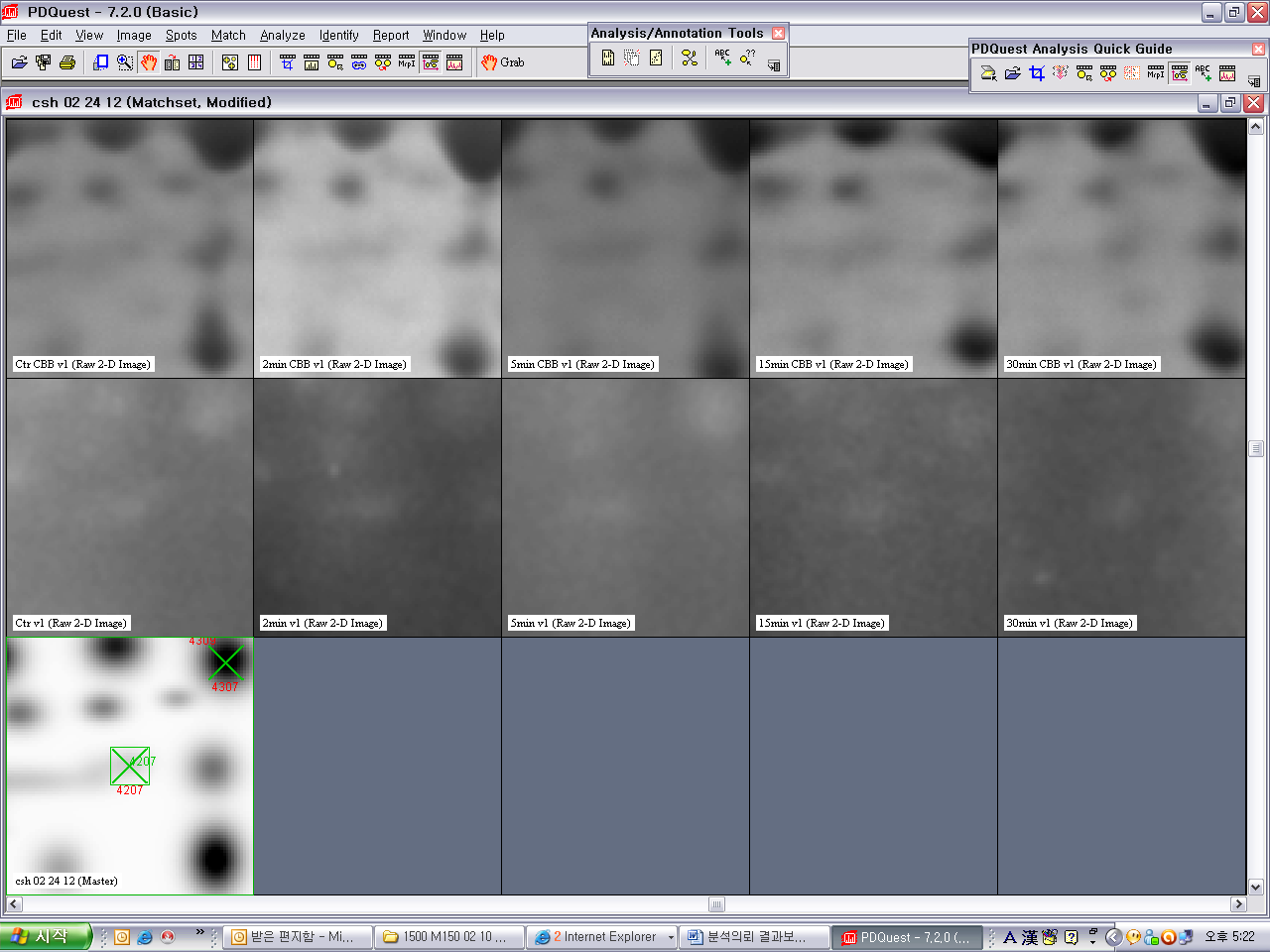


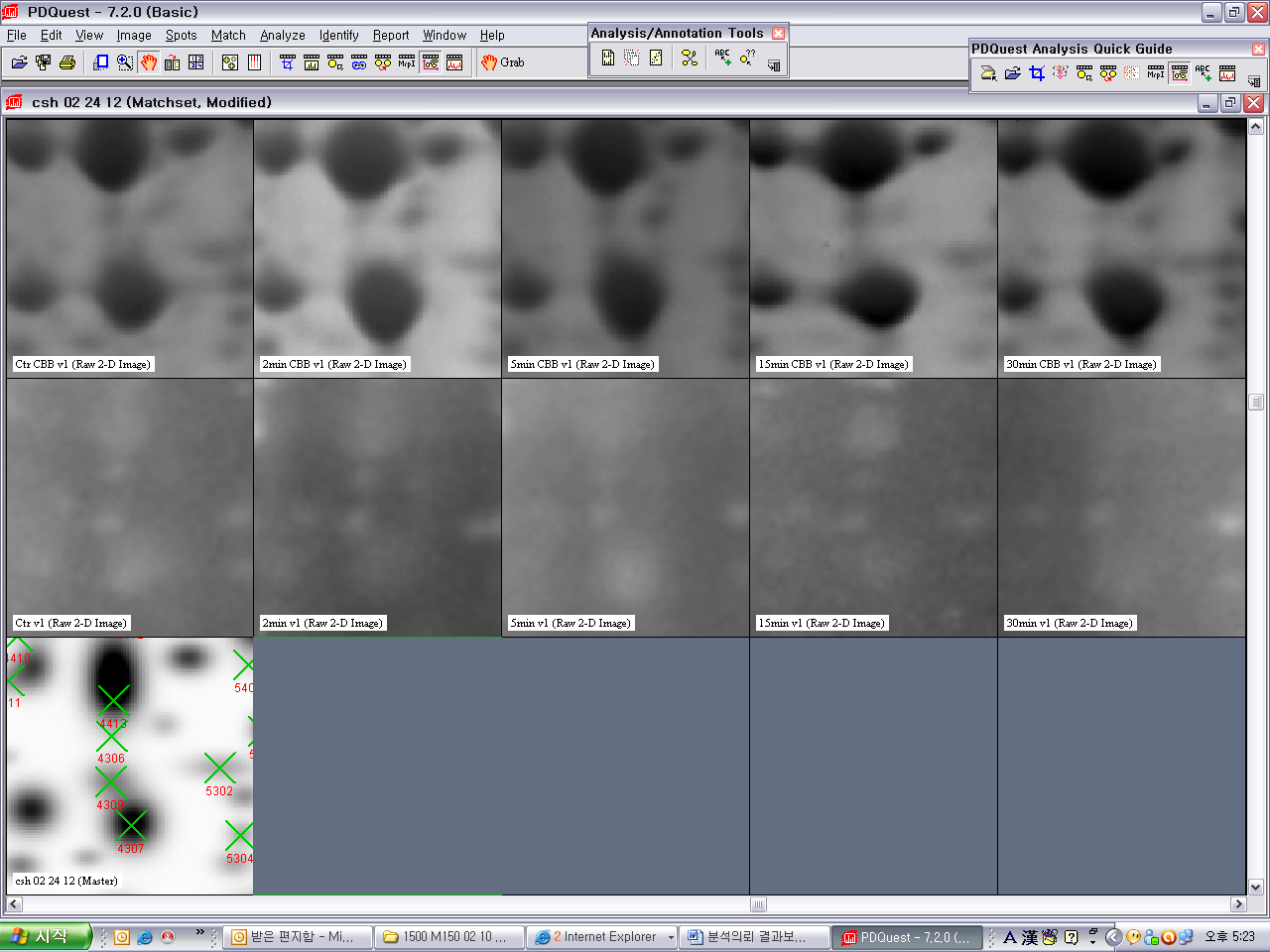


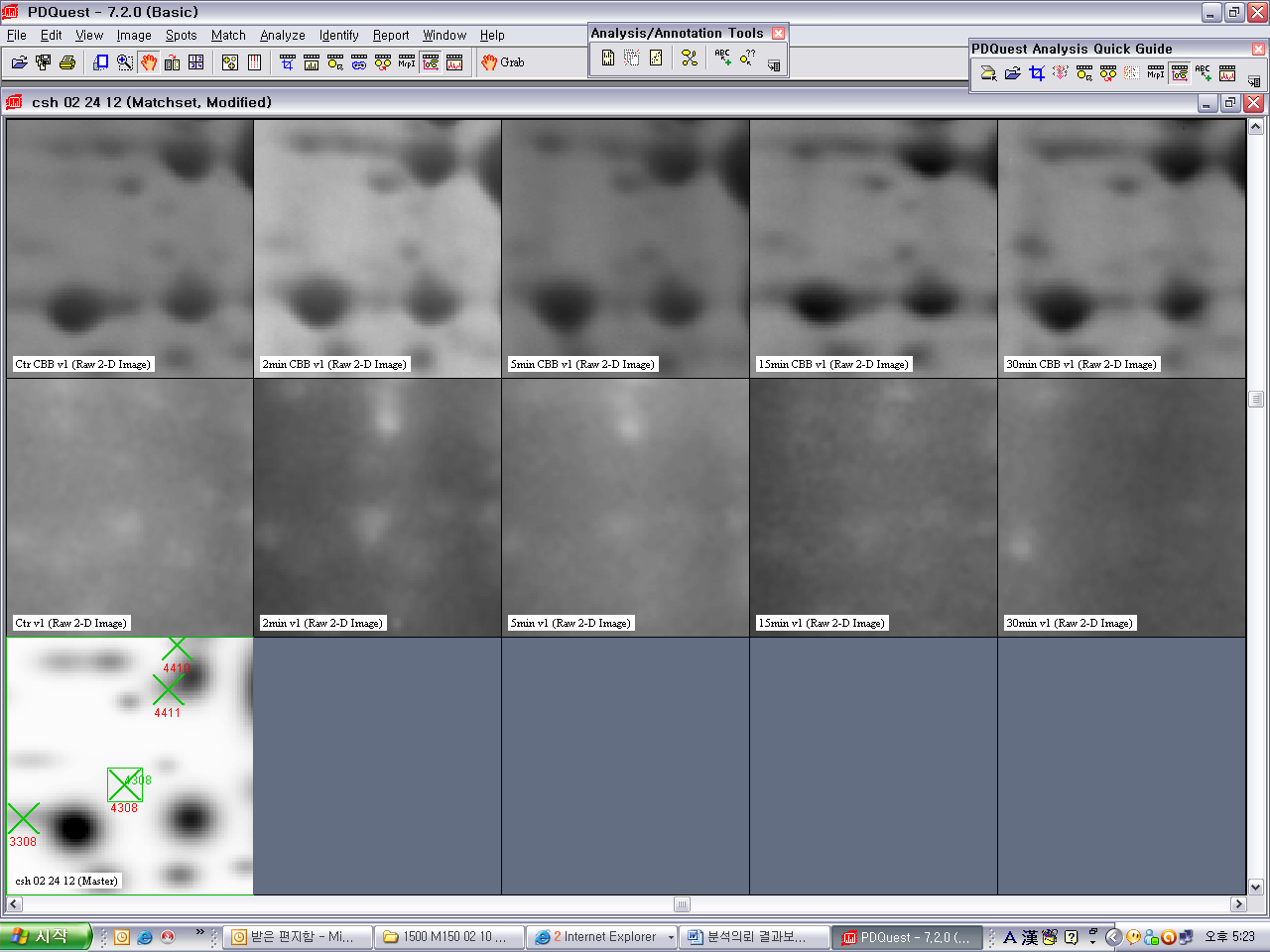


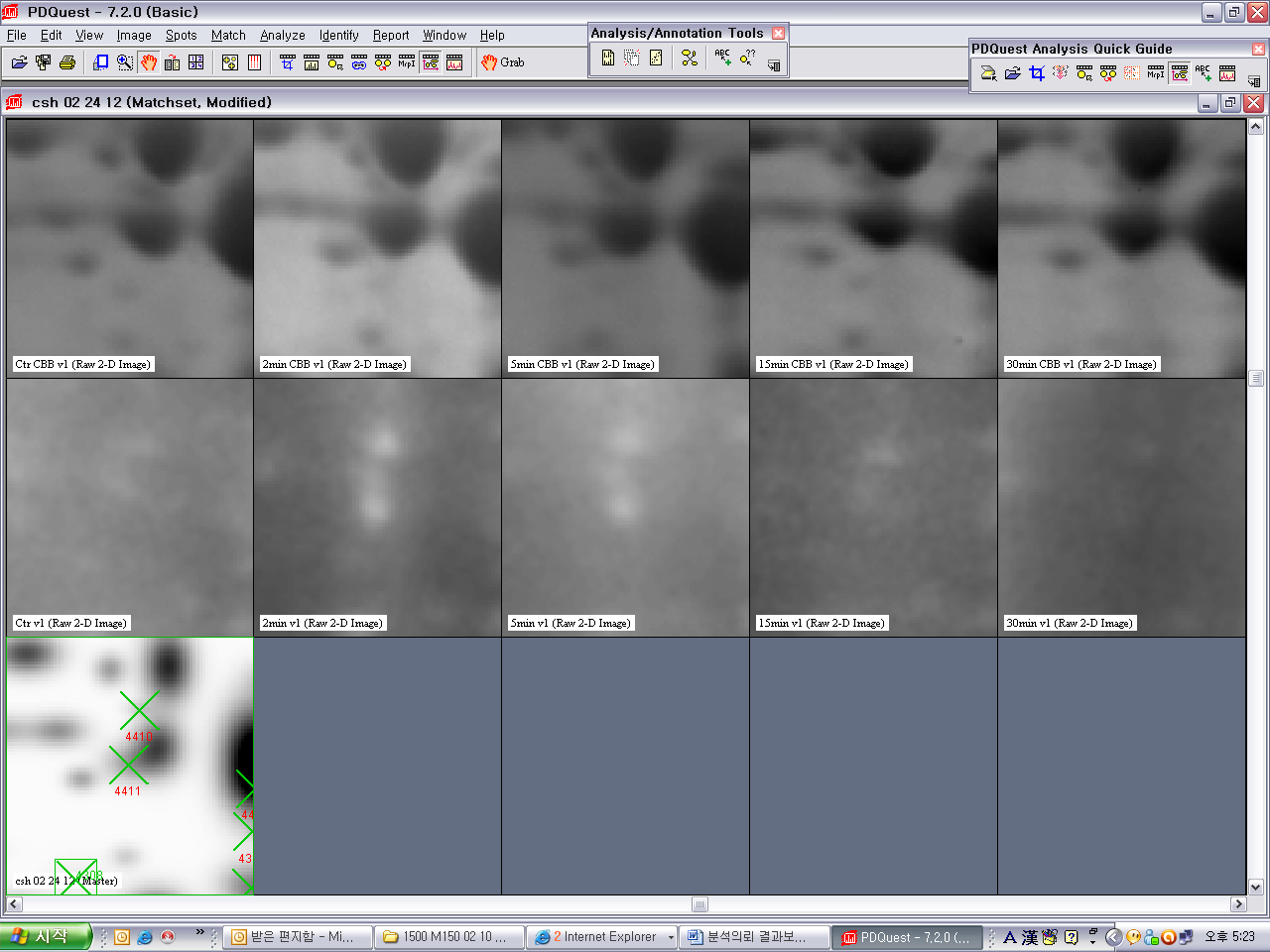


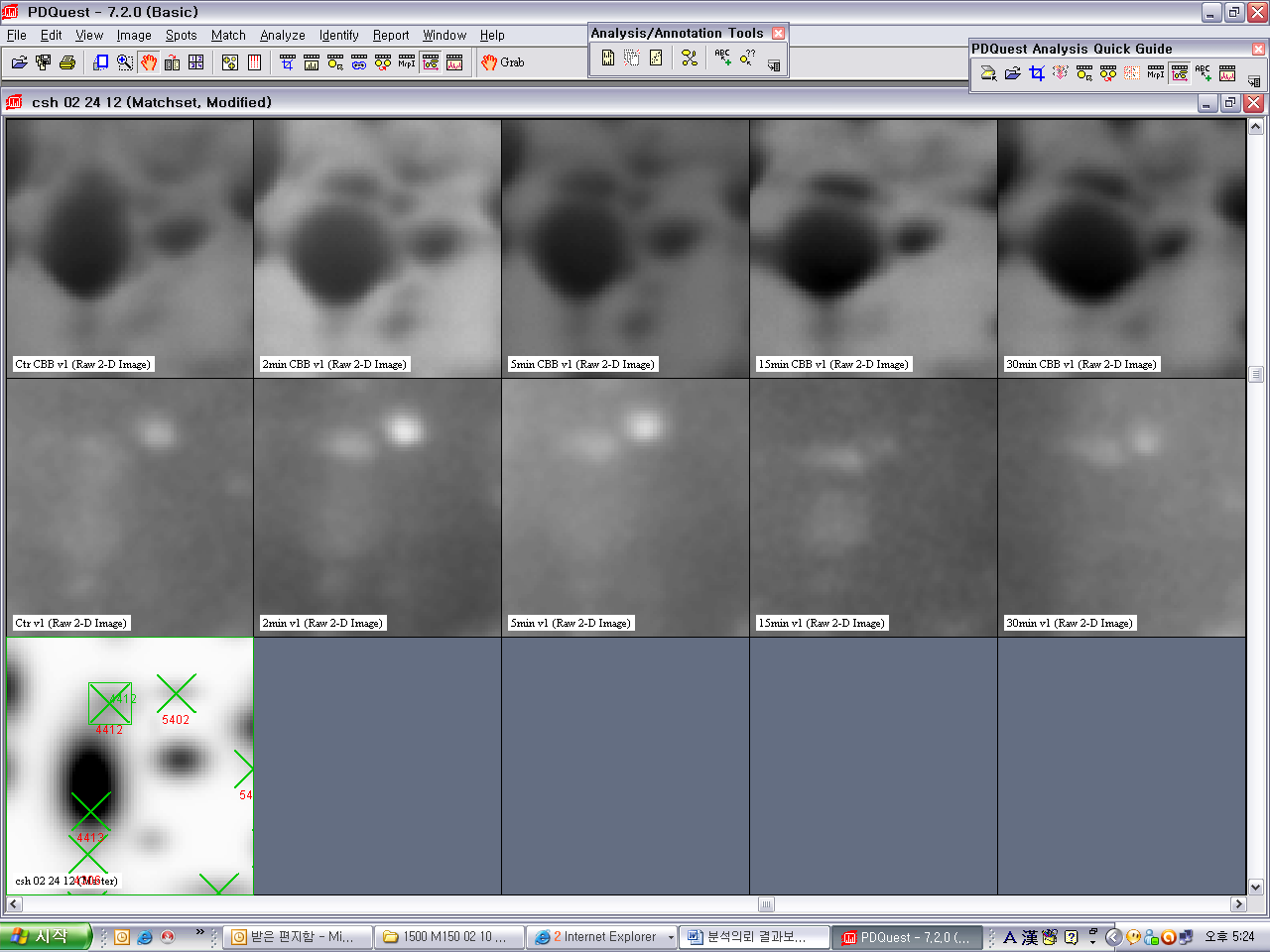


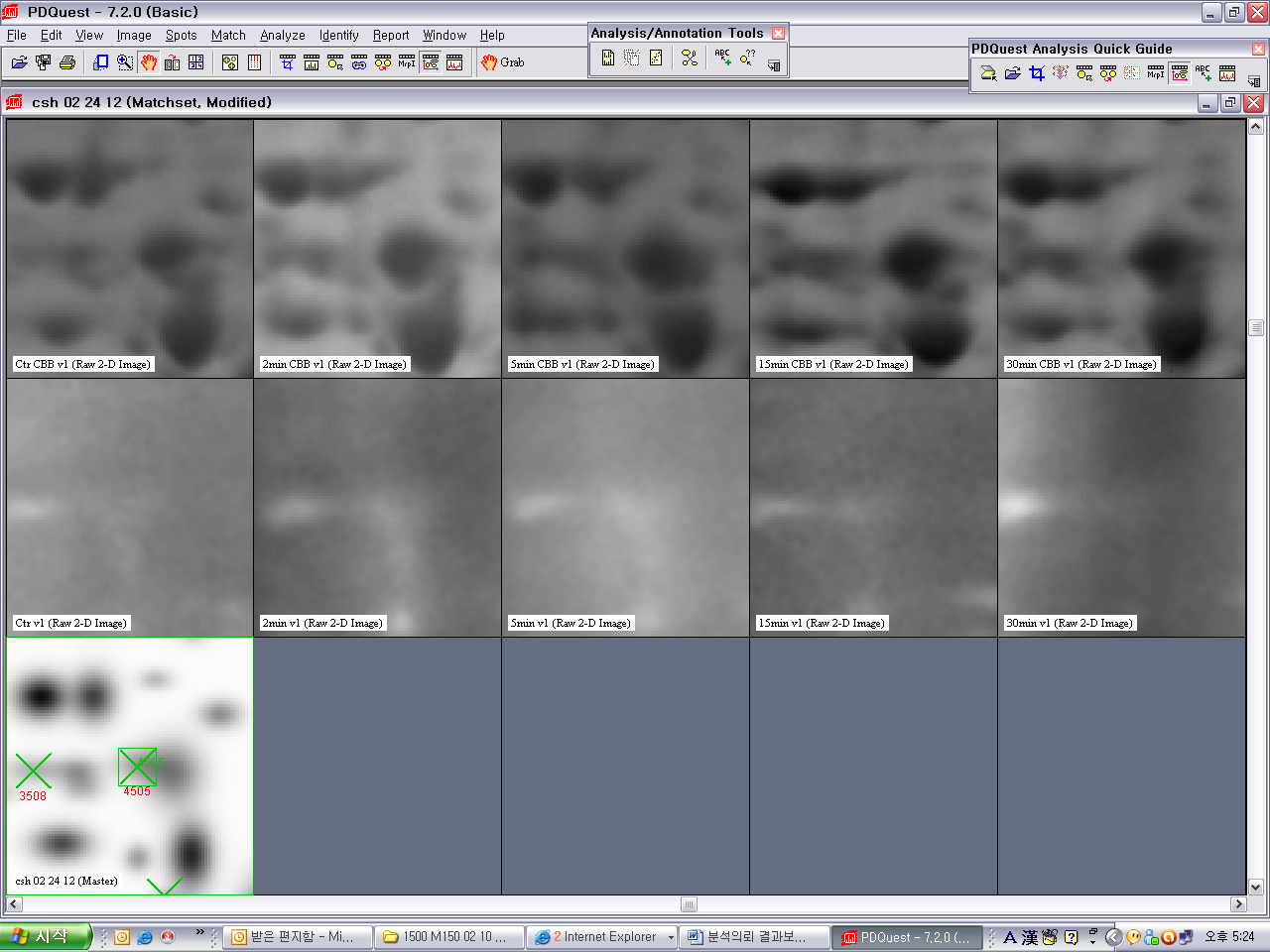


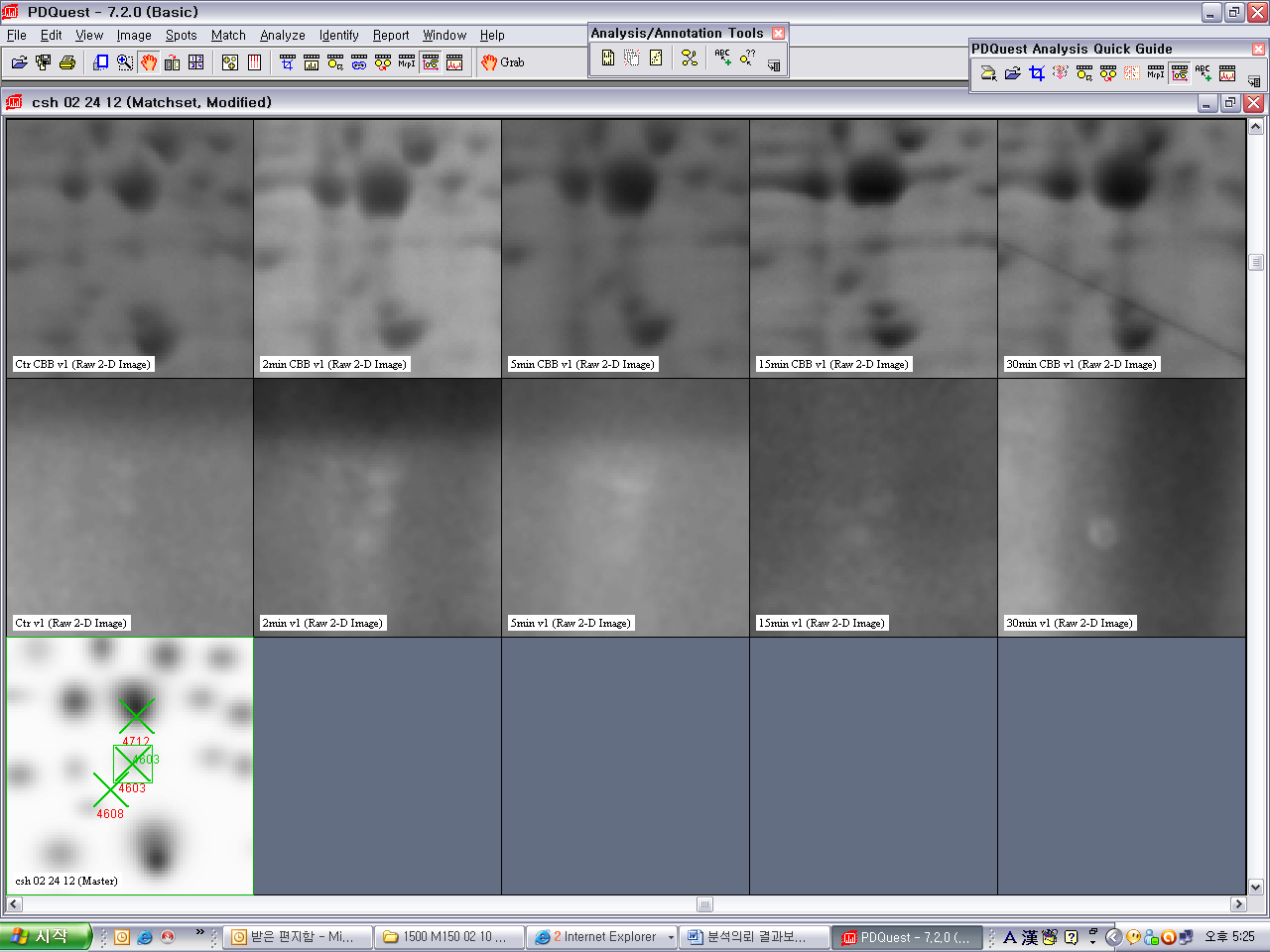


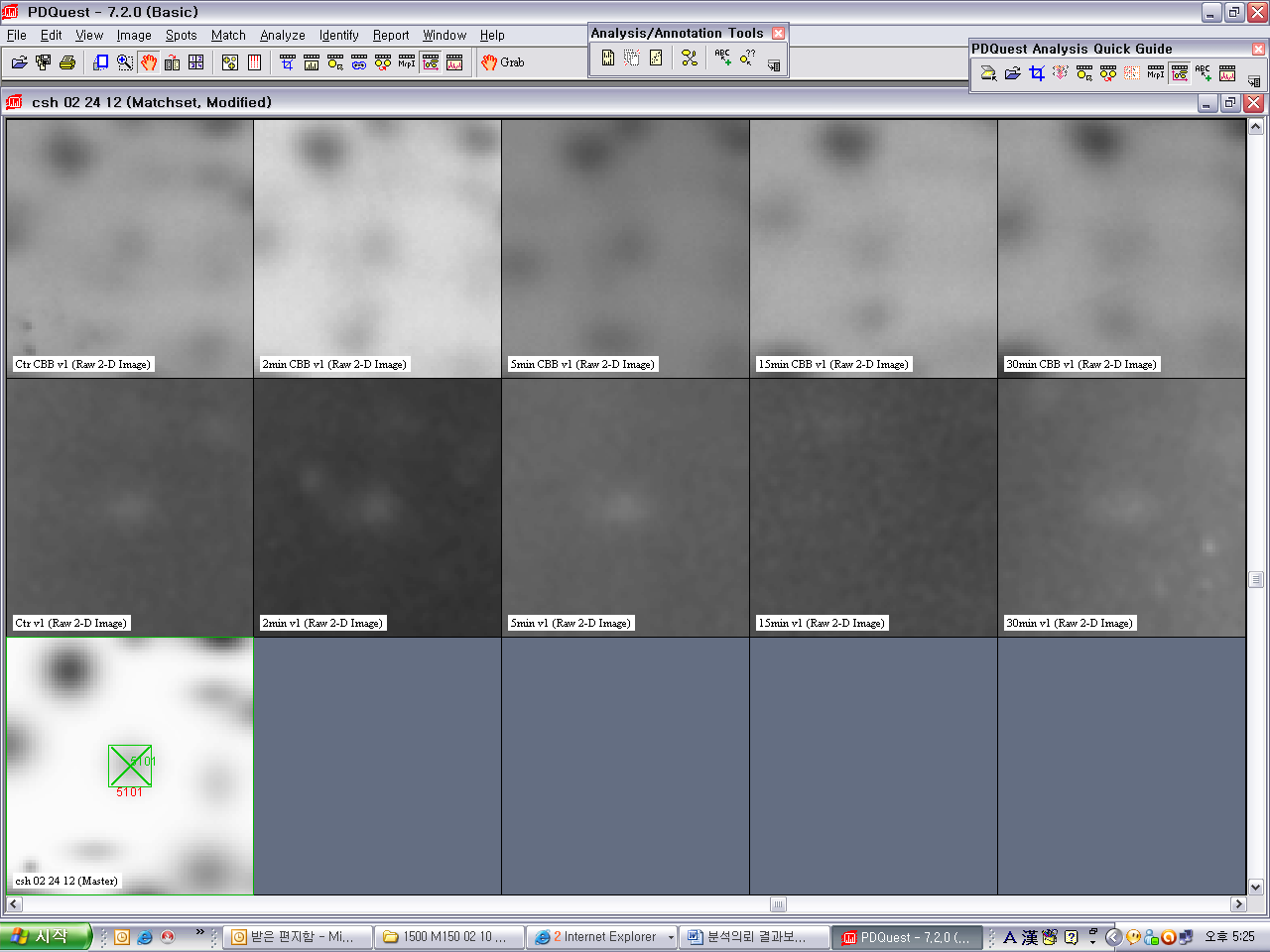


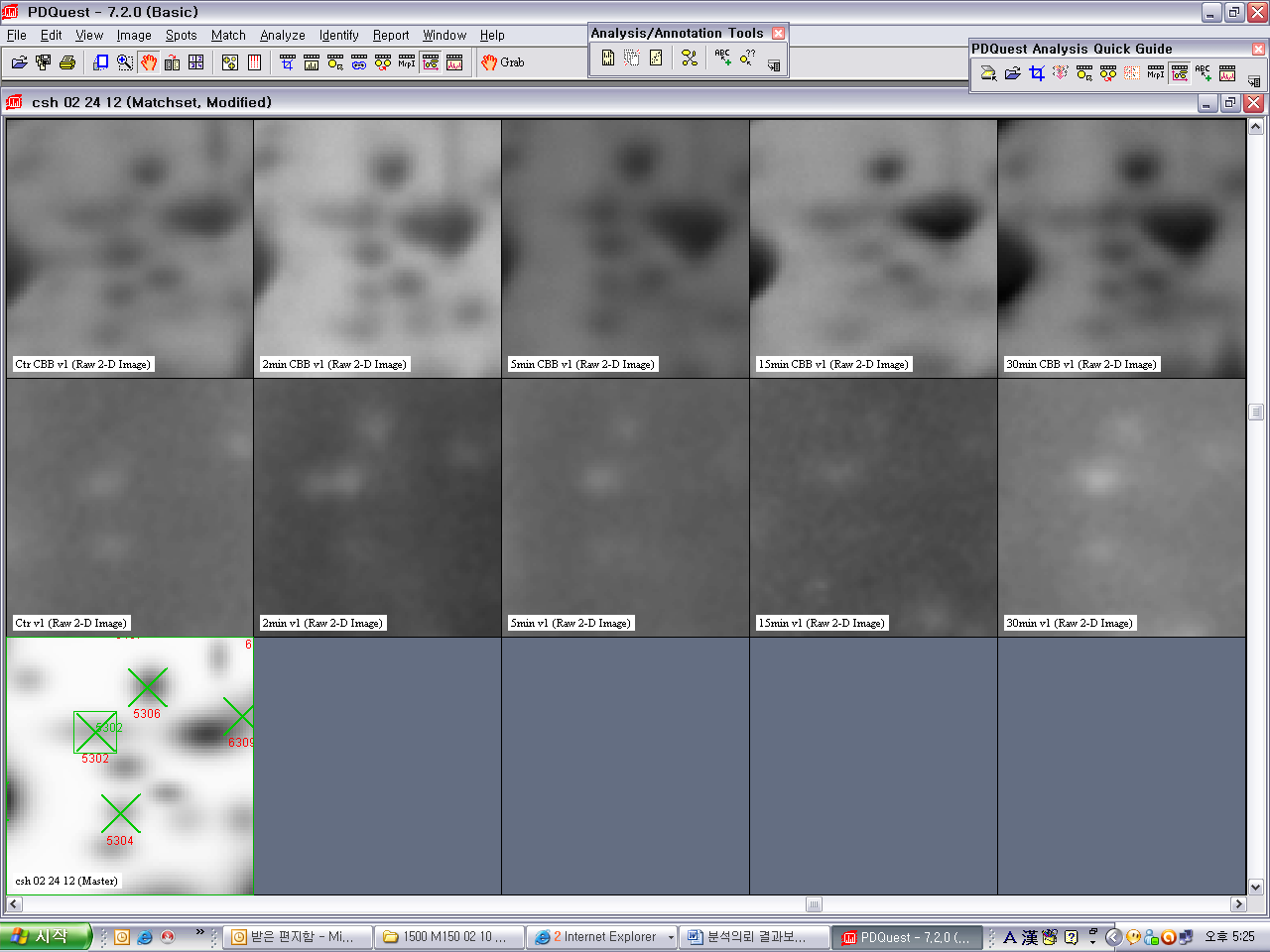


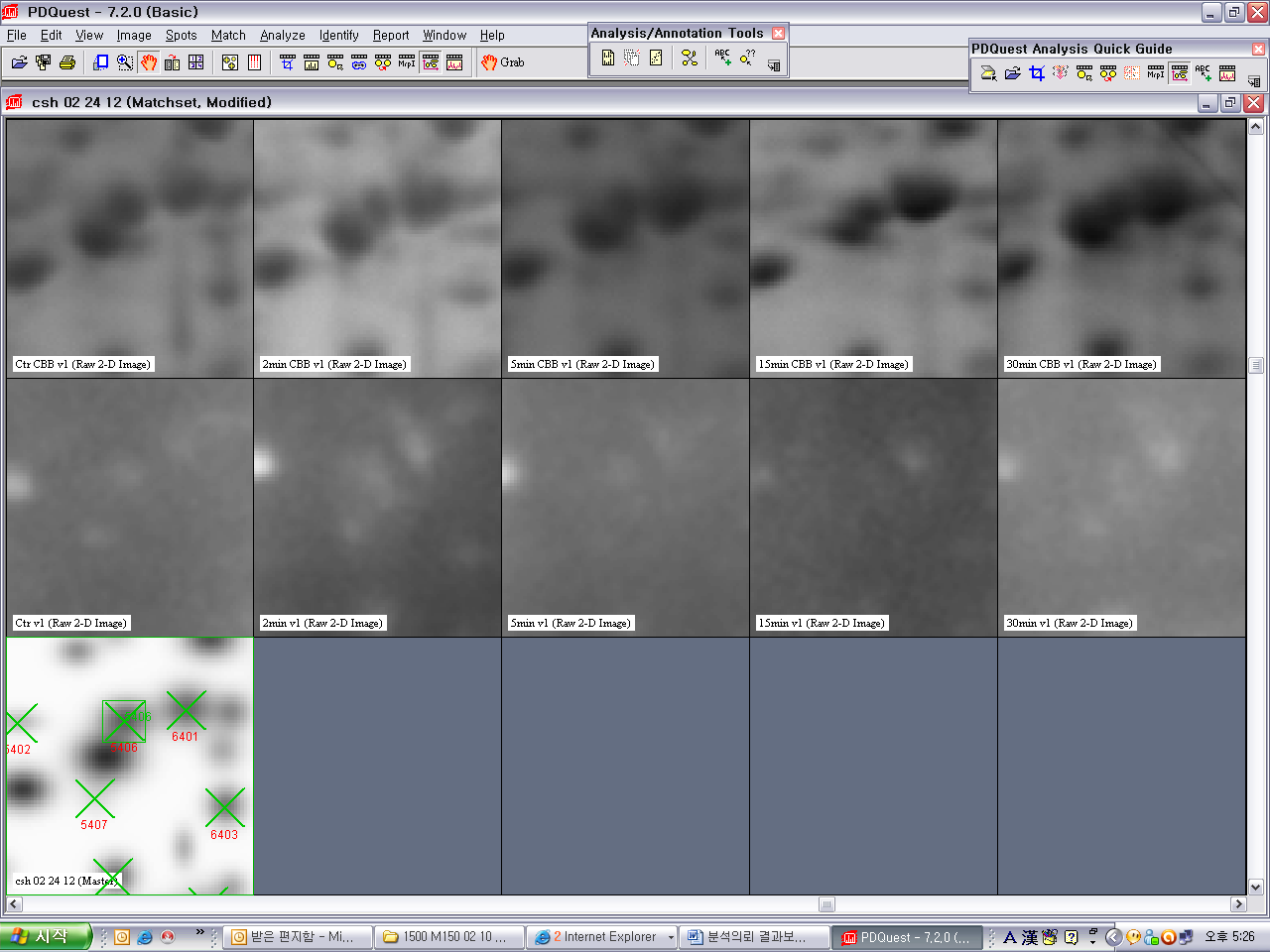


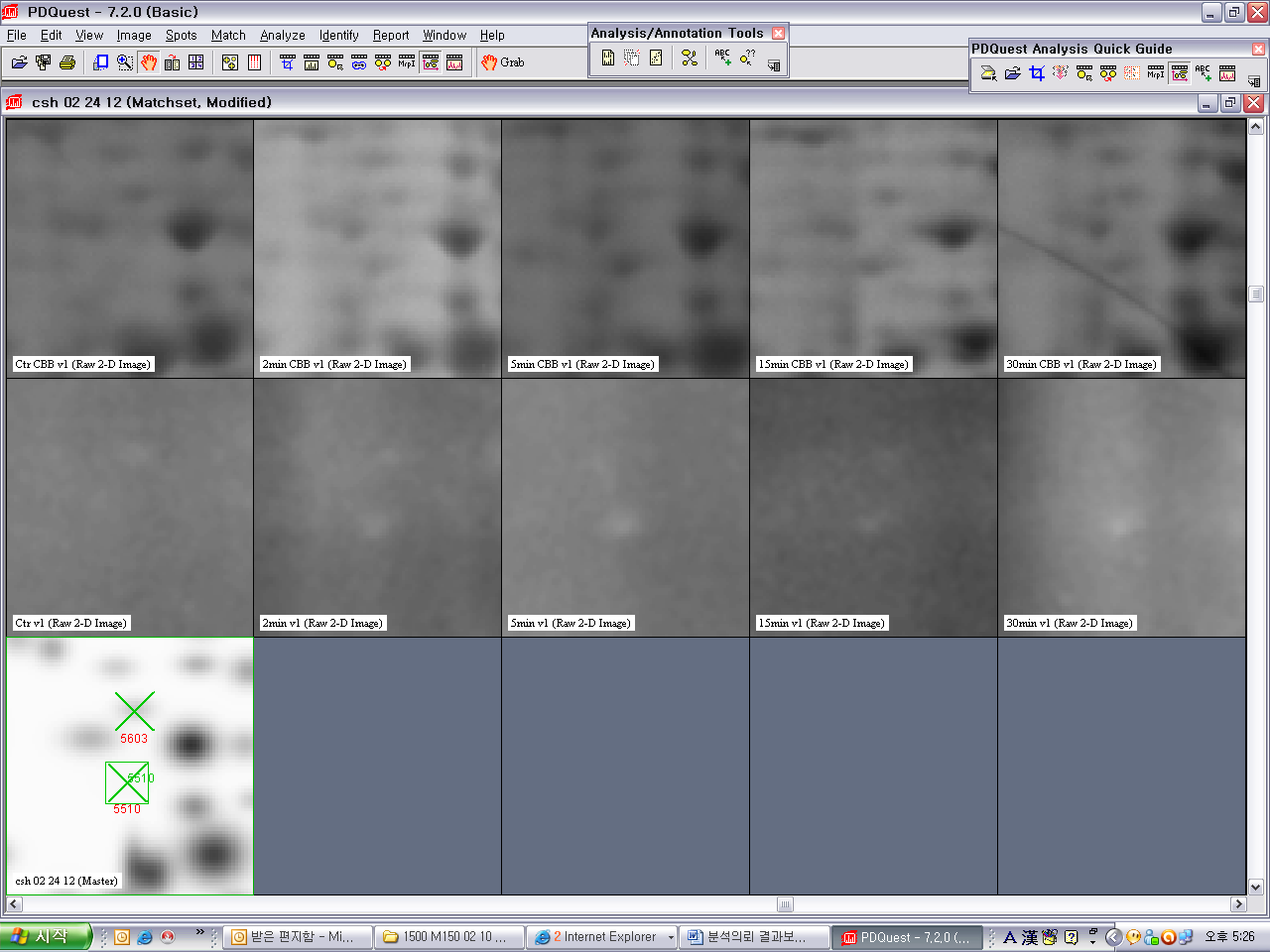


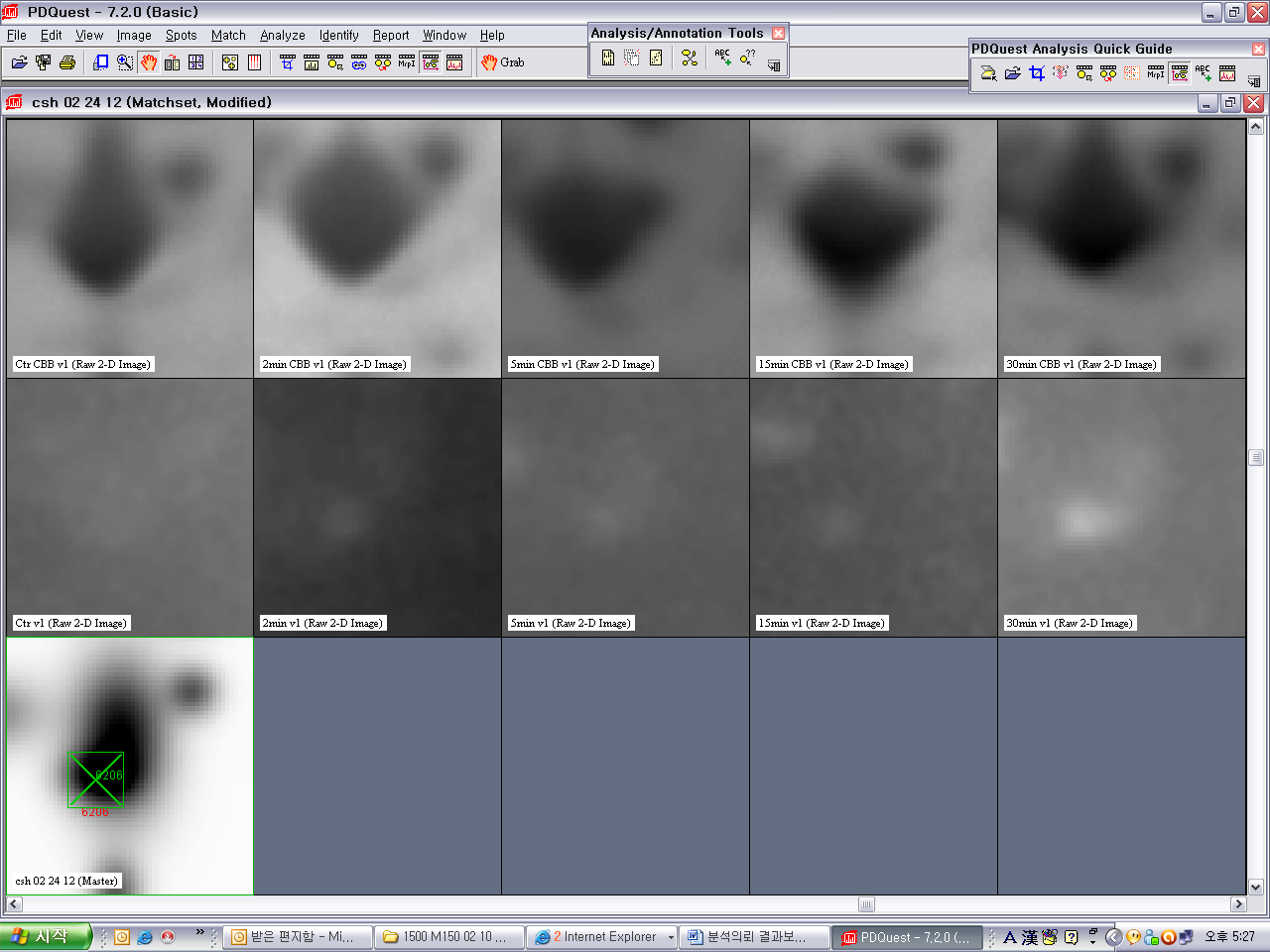


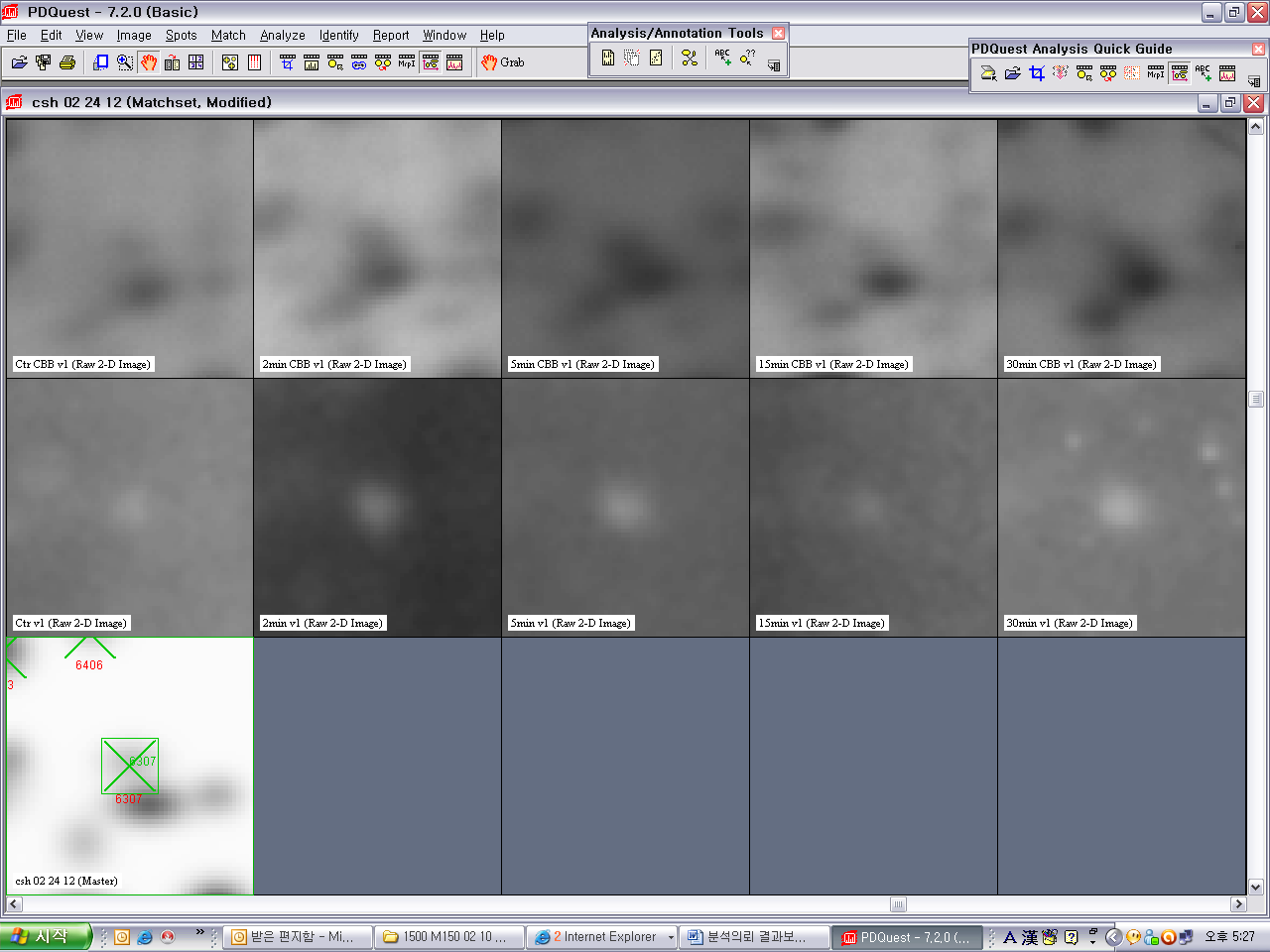


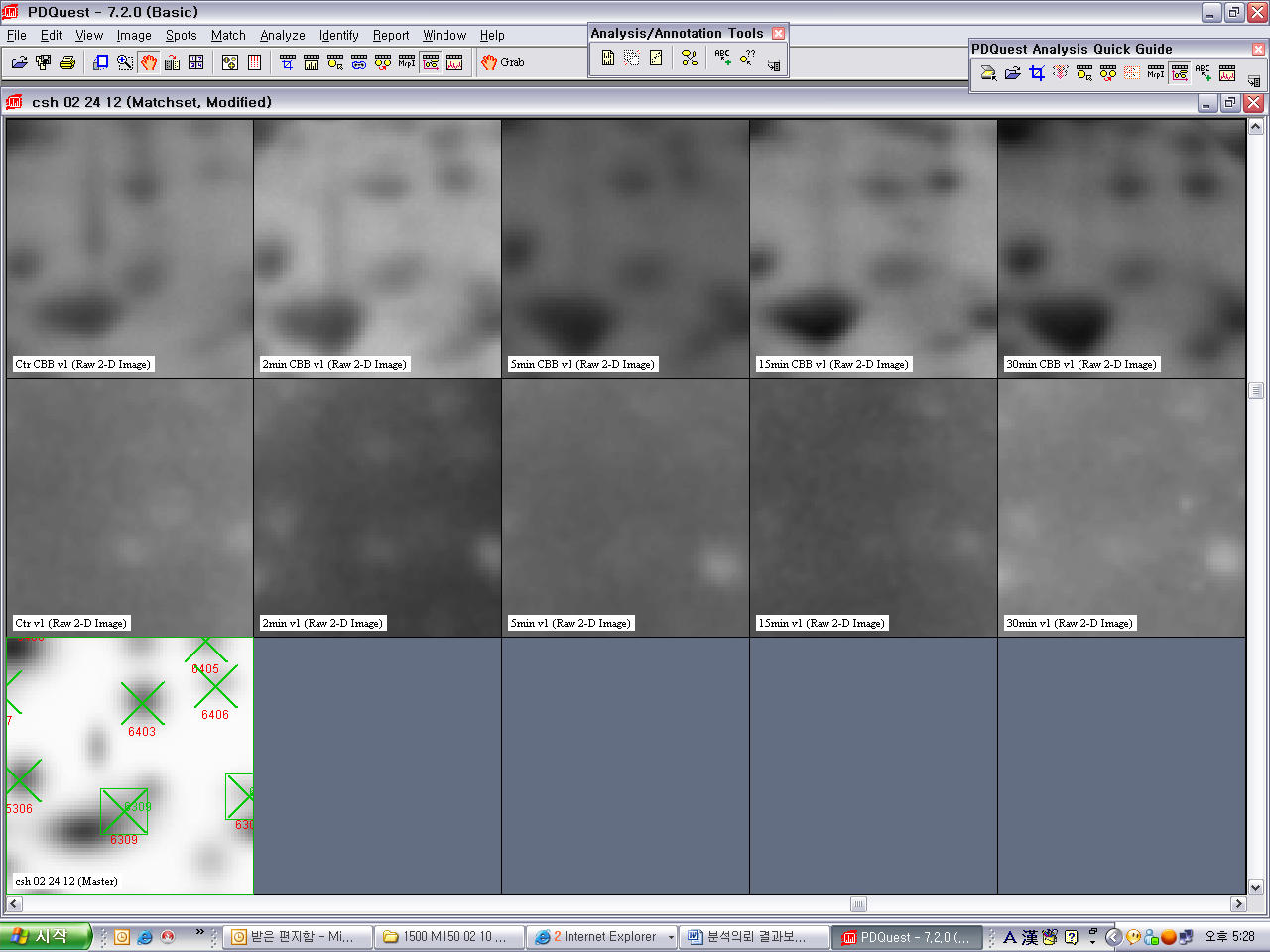


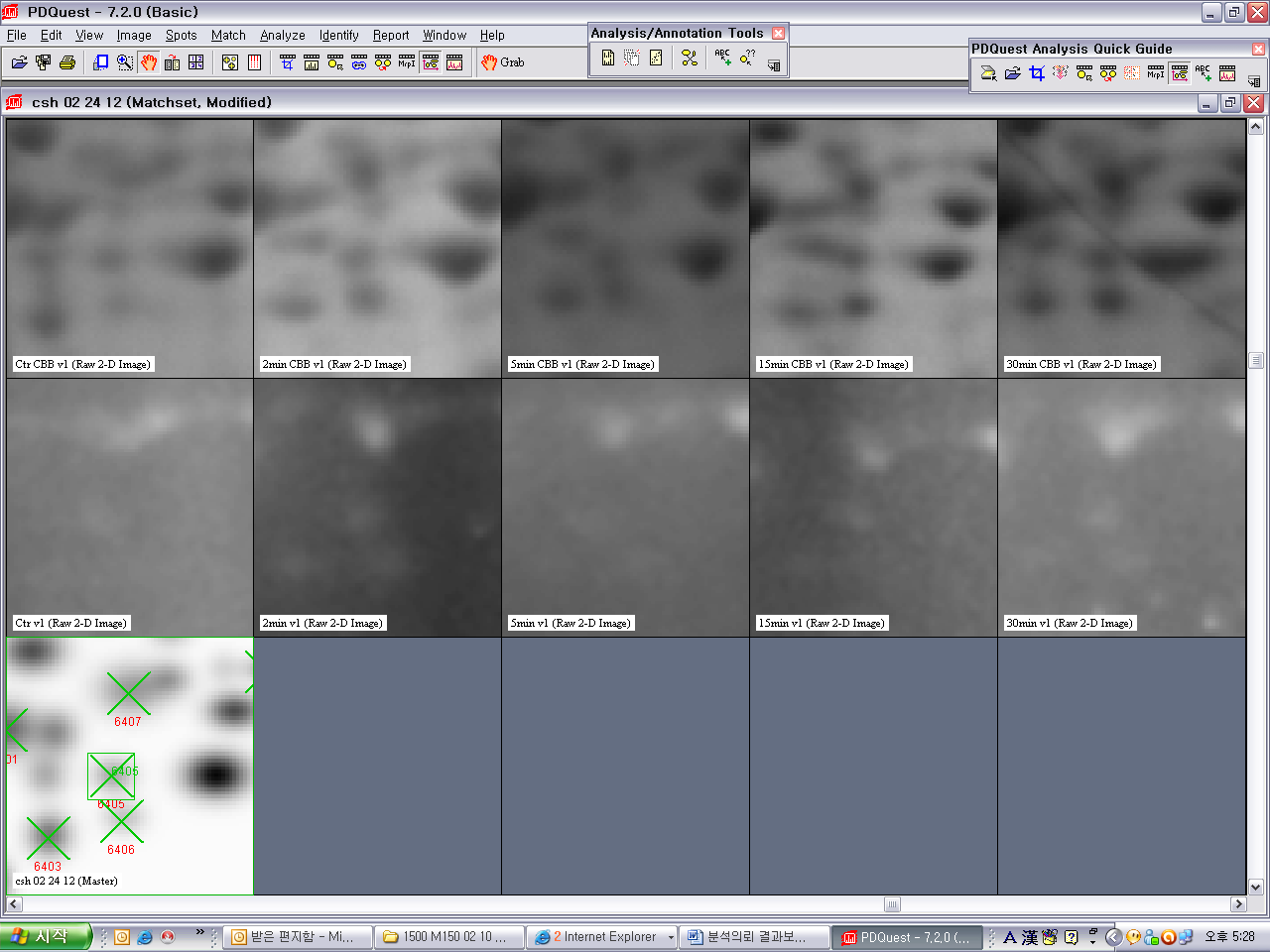


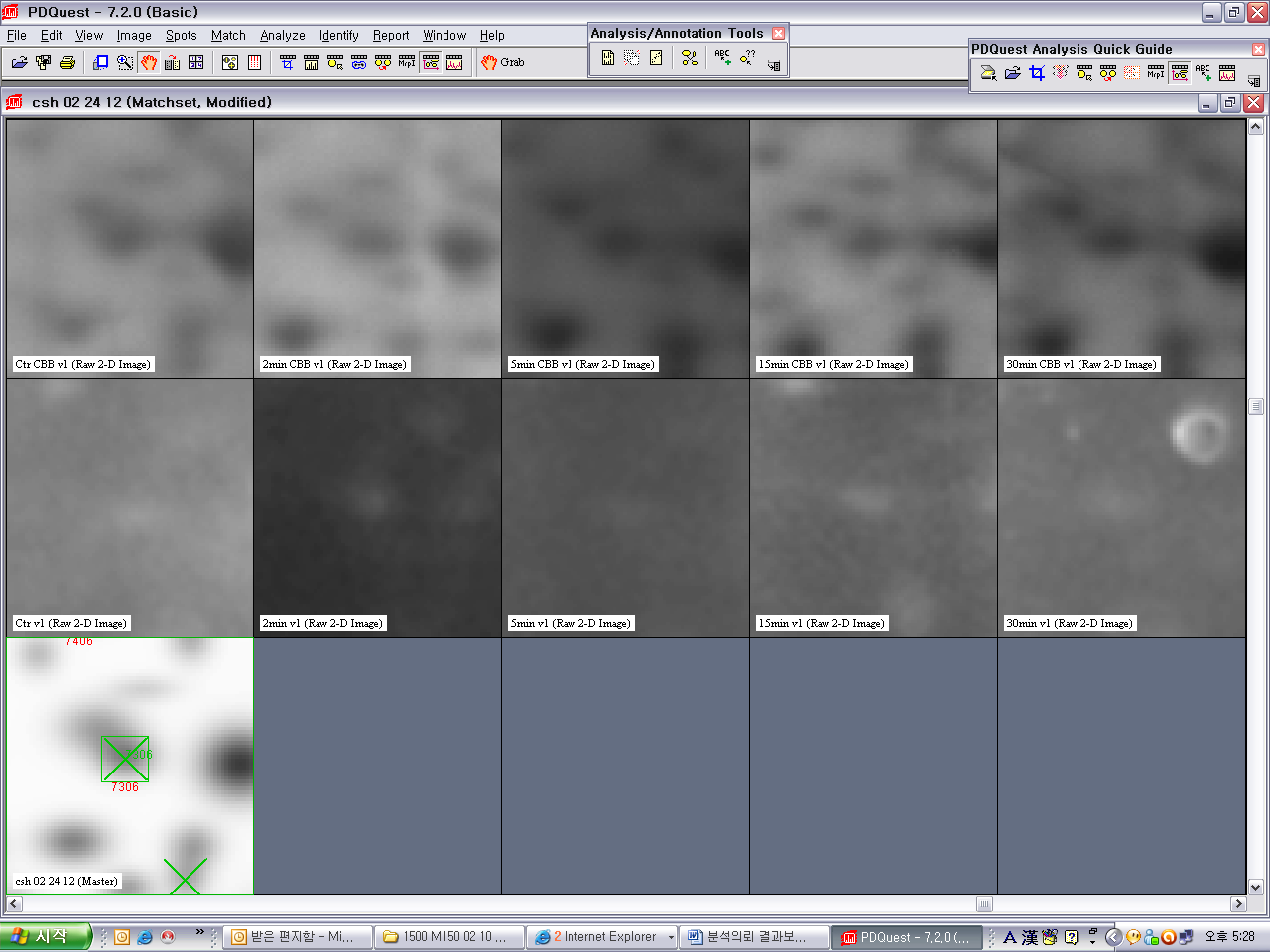


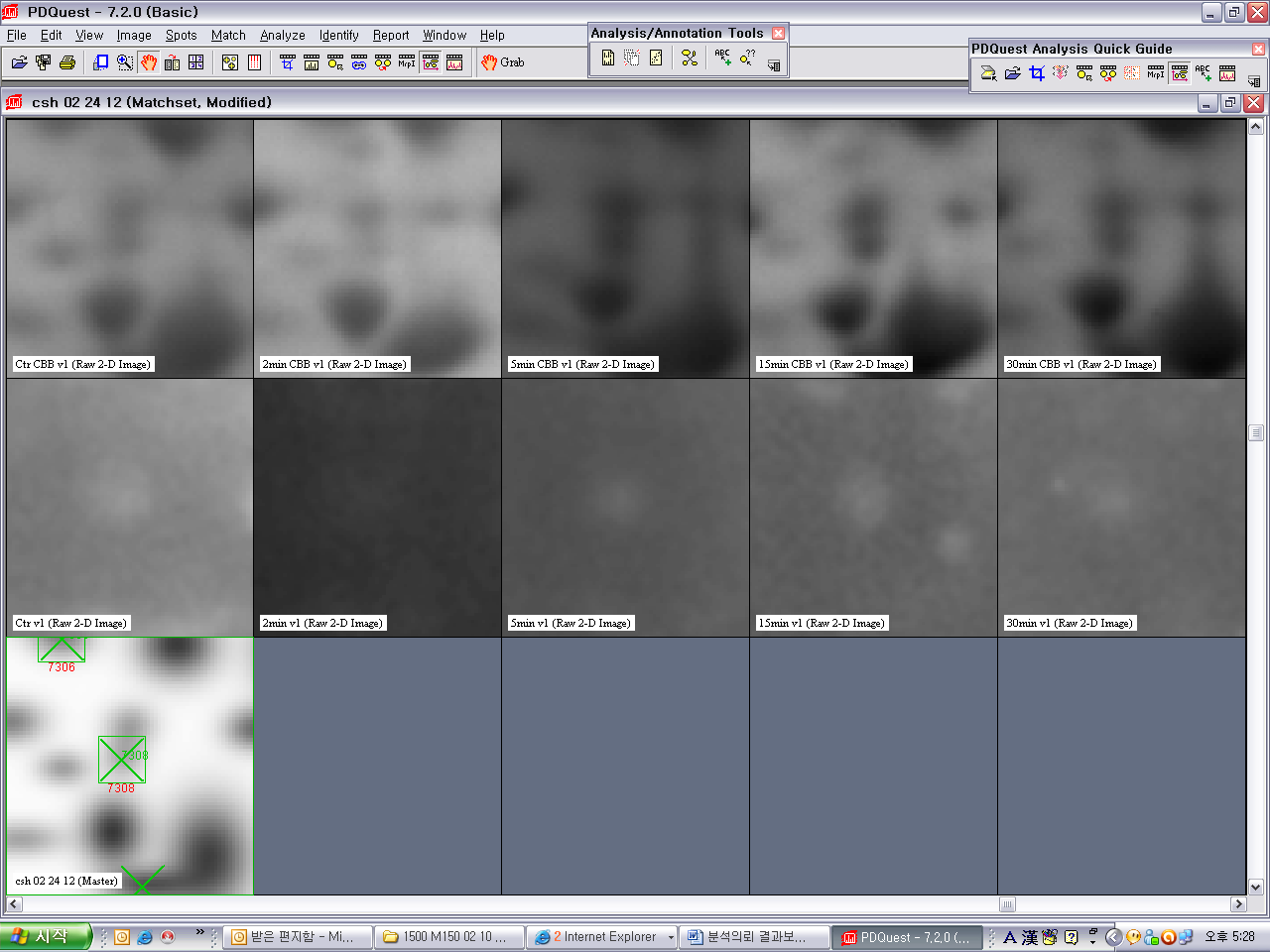


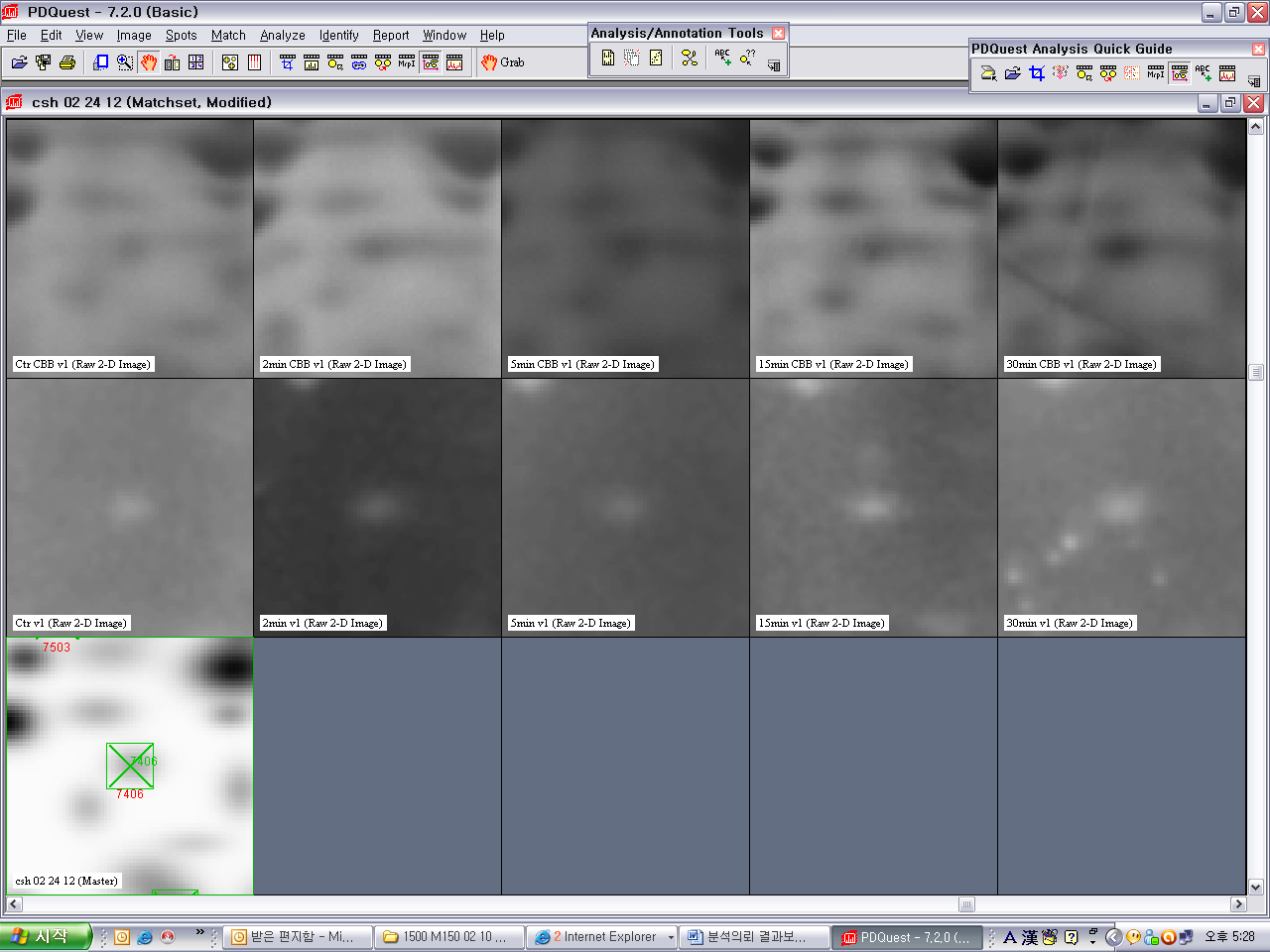


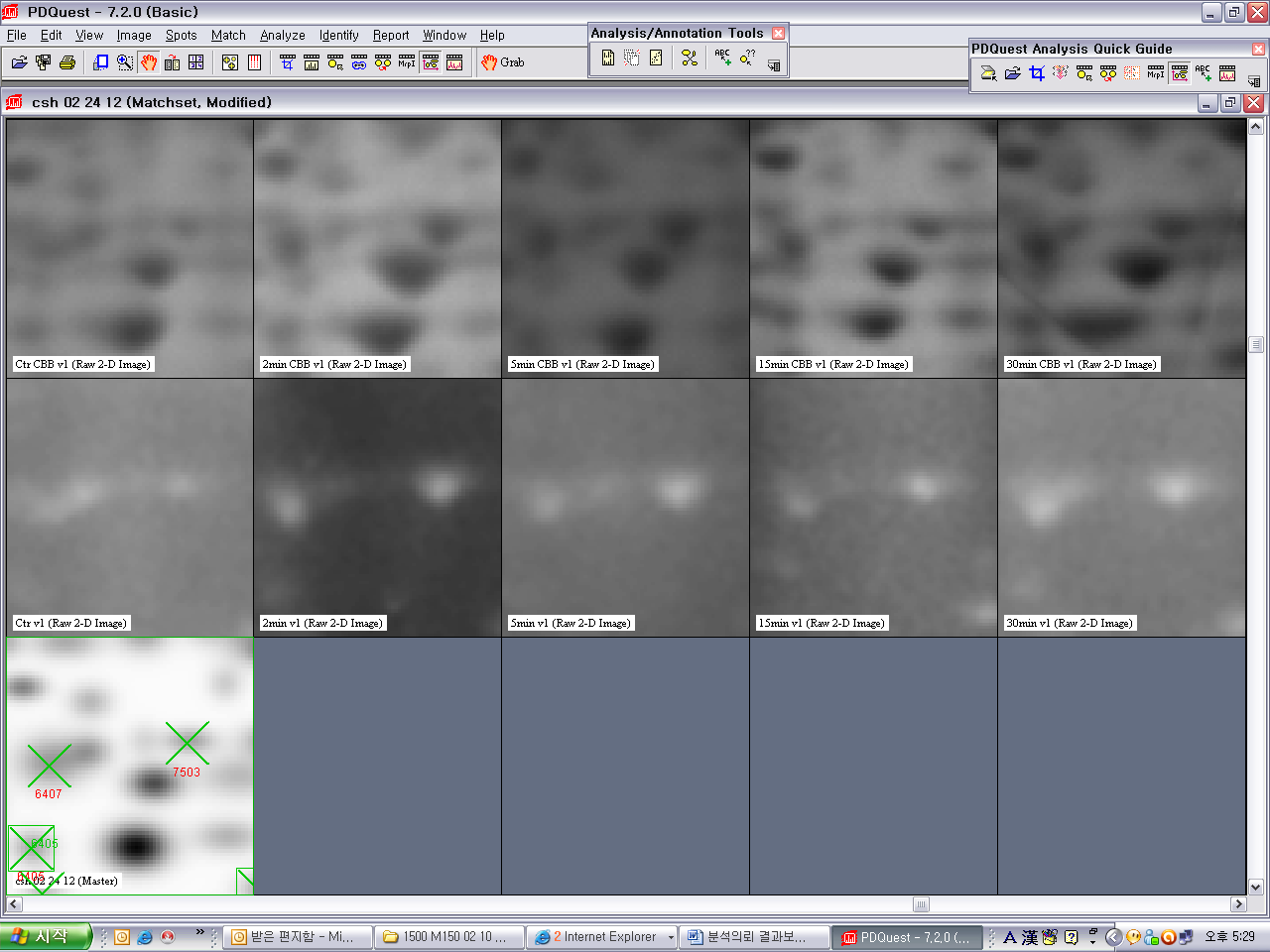


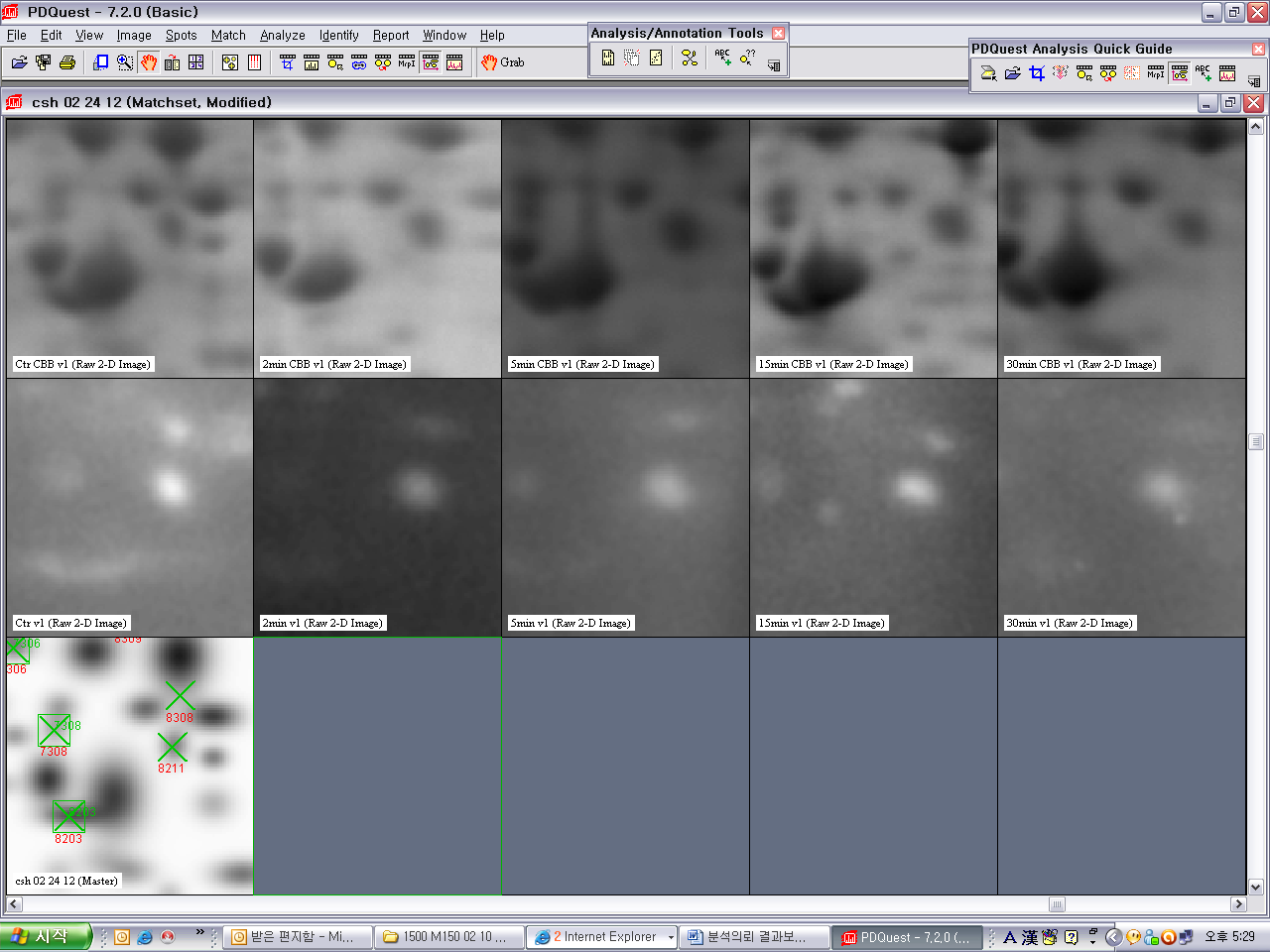


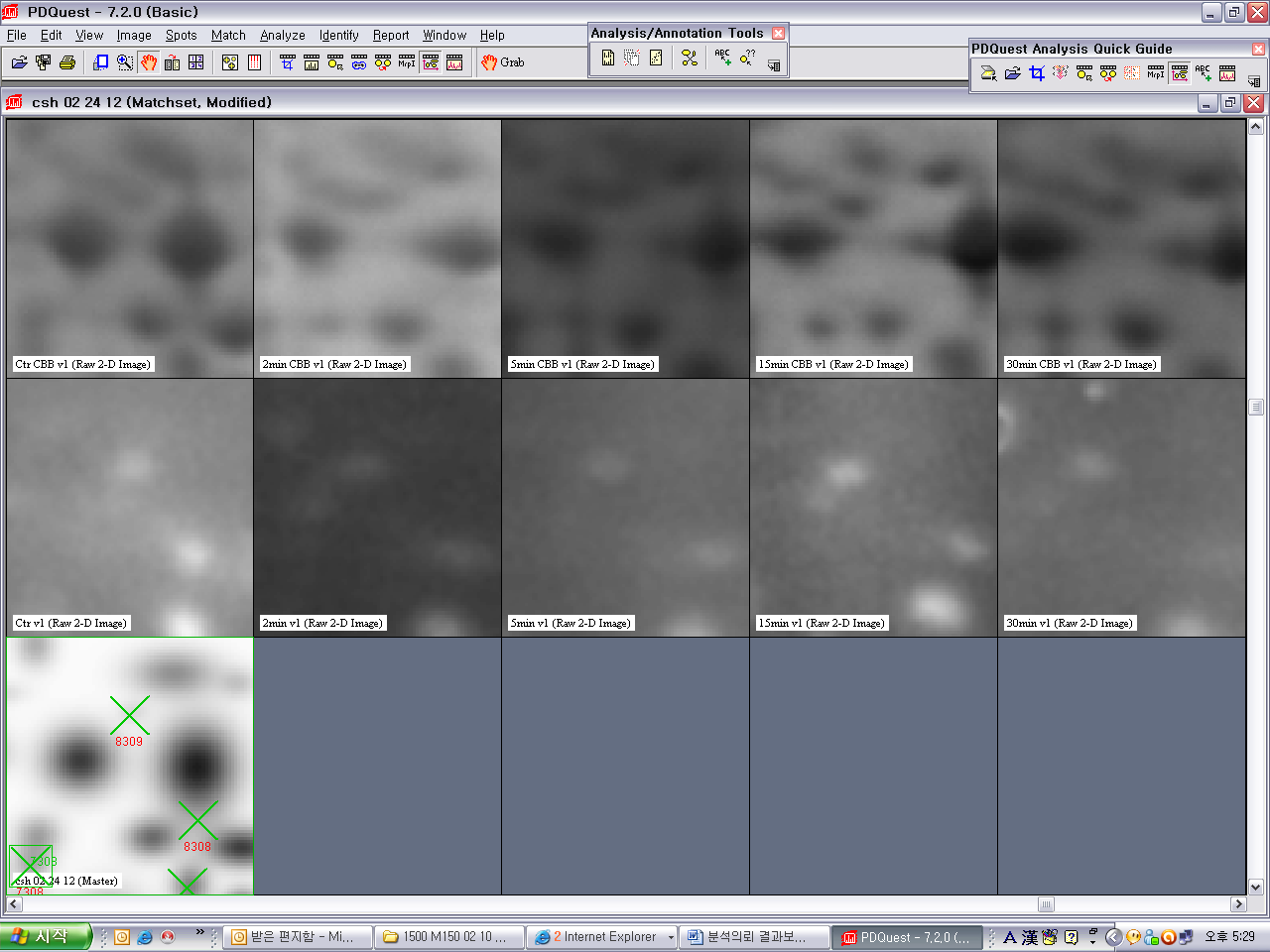


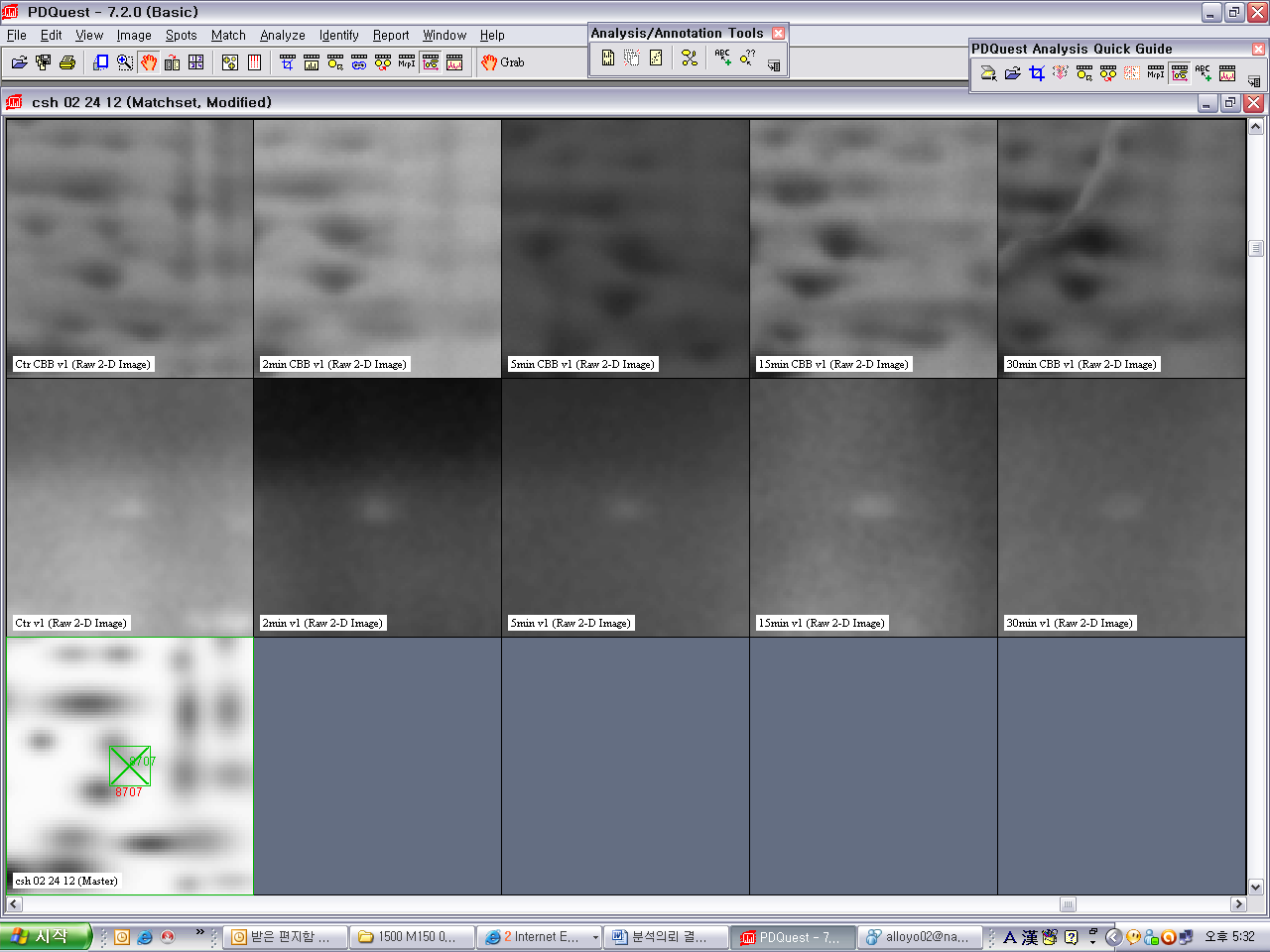


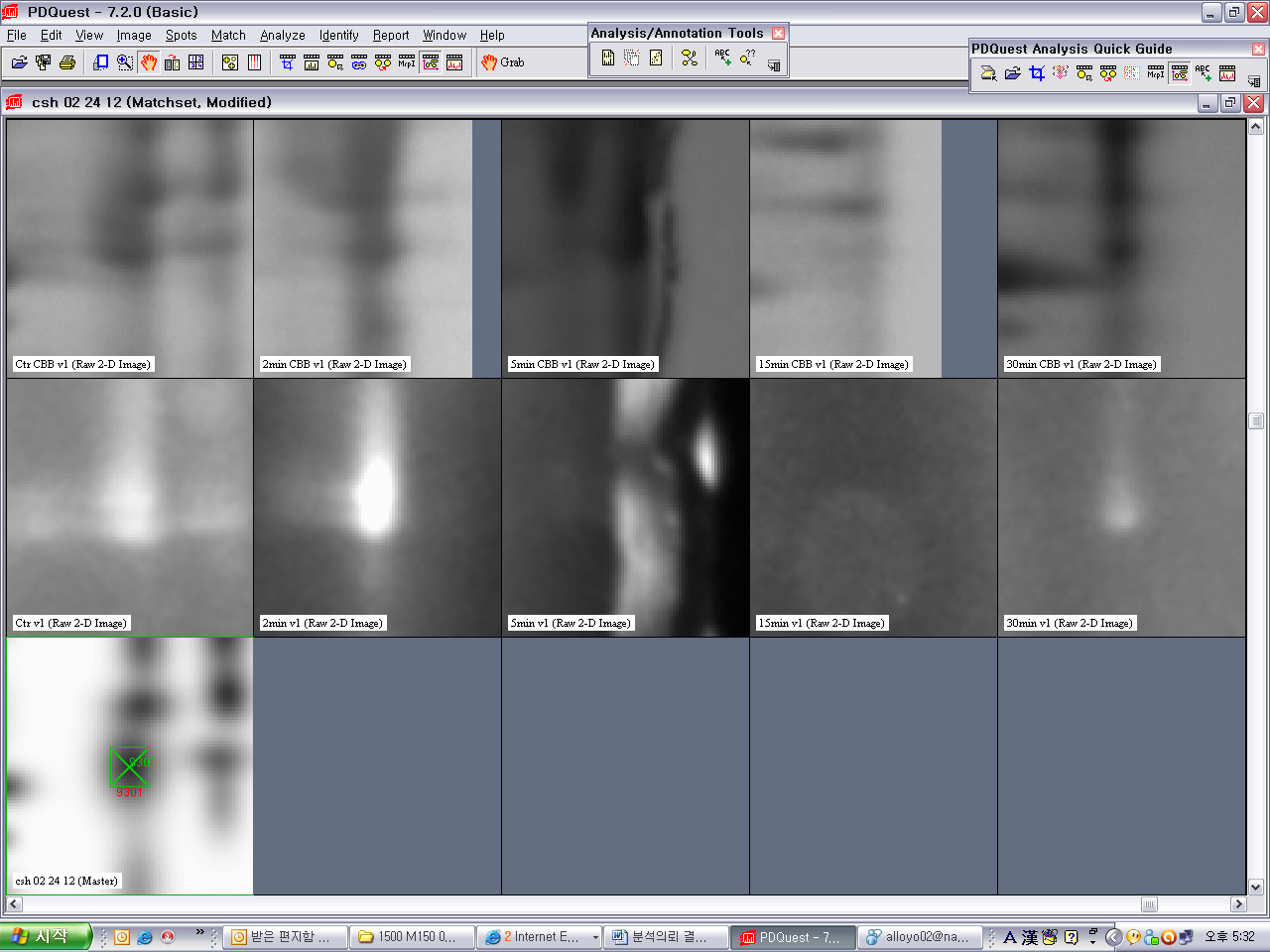


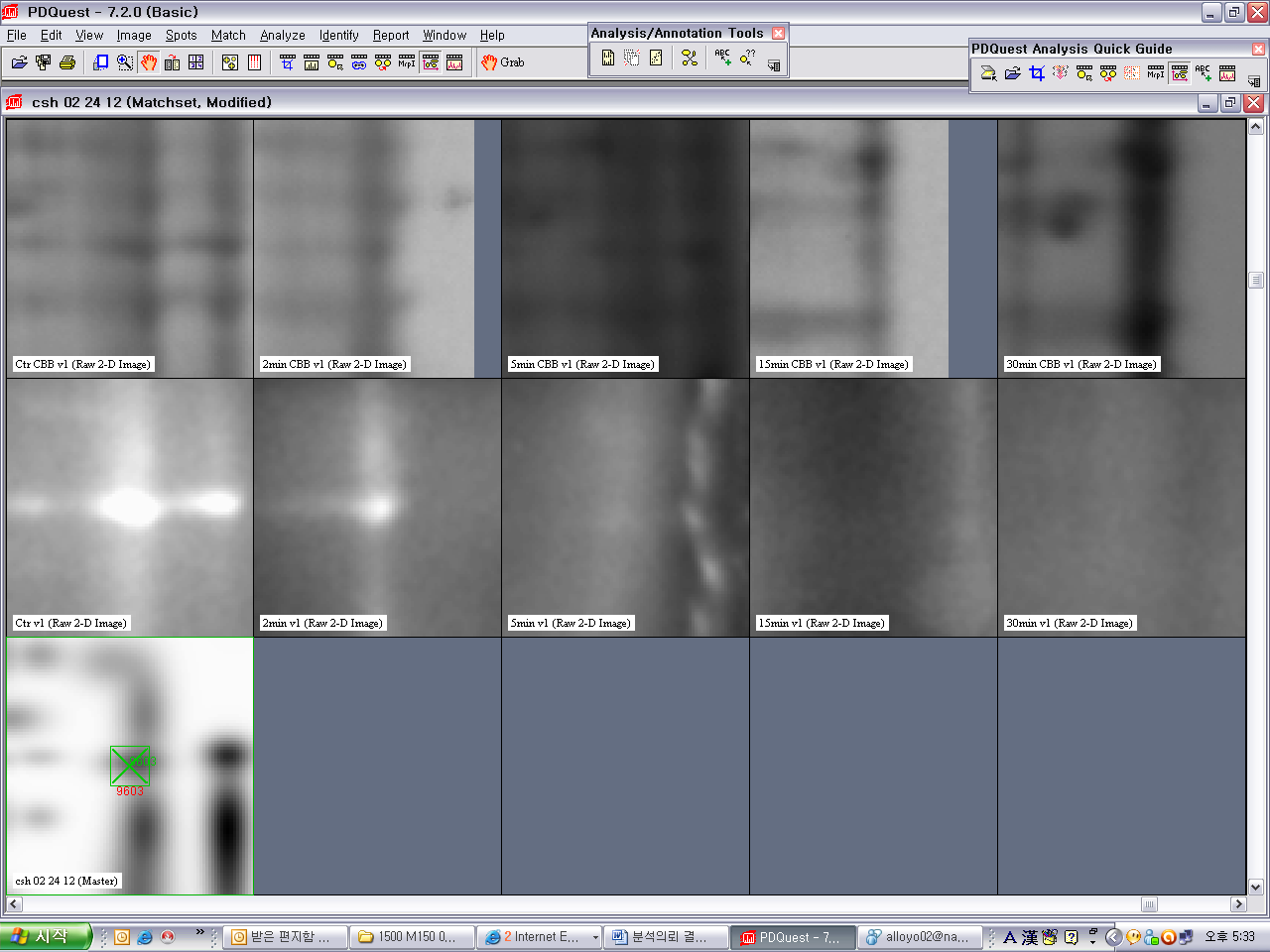

Supplement: S1 File — (DOC) [file pone.0162214.s001.doc]
