# Supplementary figures and images for: Phosphoproteome Profiling of SH-SY5y Neuroblastoma Cells Treated with Anesthetics: Sevoflurane and Isoflurane Affect the Phosphorylation of Proteins Involved in Cytoskeletal Regulation
Source: PLoS One. 2016 Sep 9;11(9):e0162214. doi: 10.1371/journal.pone.0162214 (PMC5017685; doi:10.1371/journal.pone.0162214)

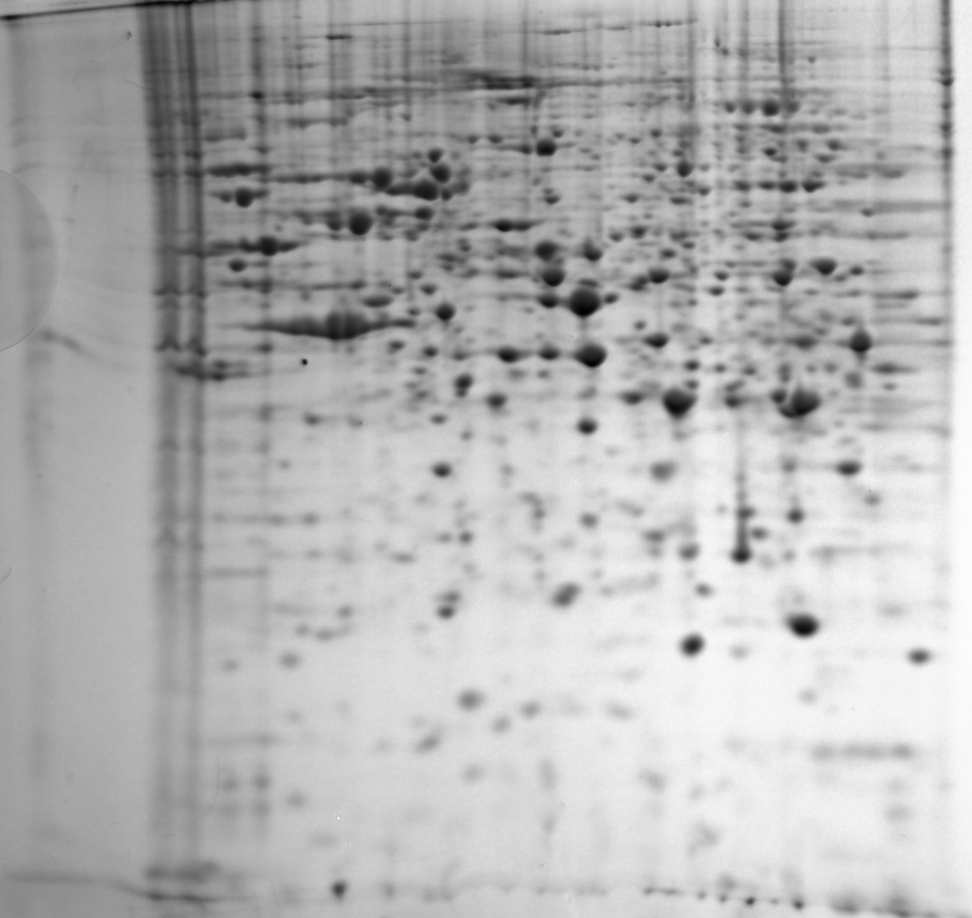

Supplement: S2 File — (ZIP) [file pone.0162214.s002.zip › 15min CBB.tif]

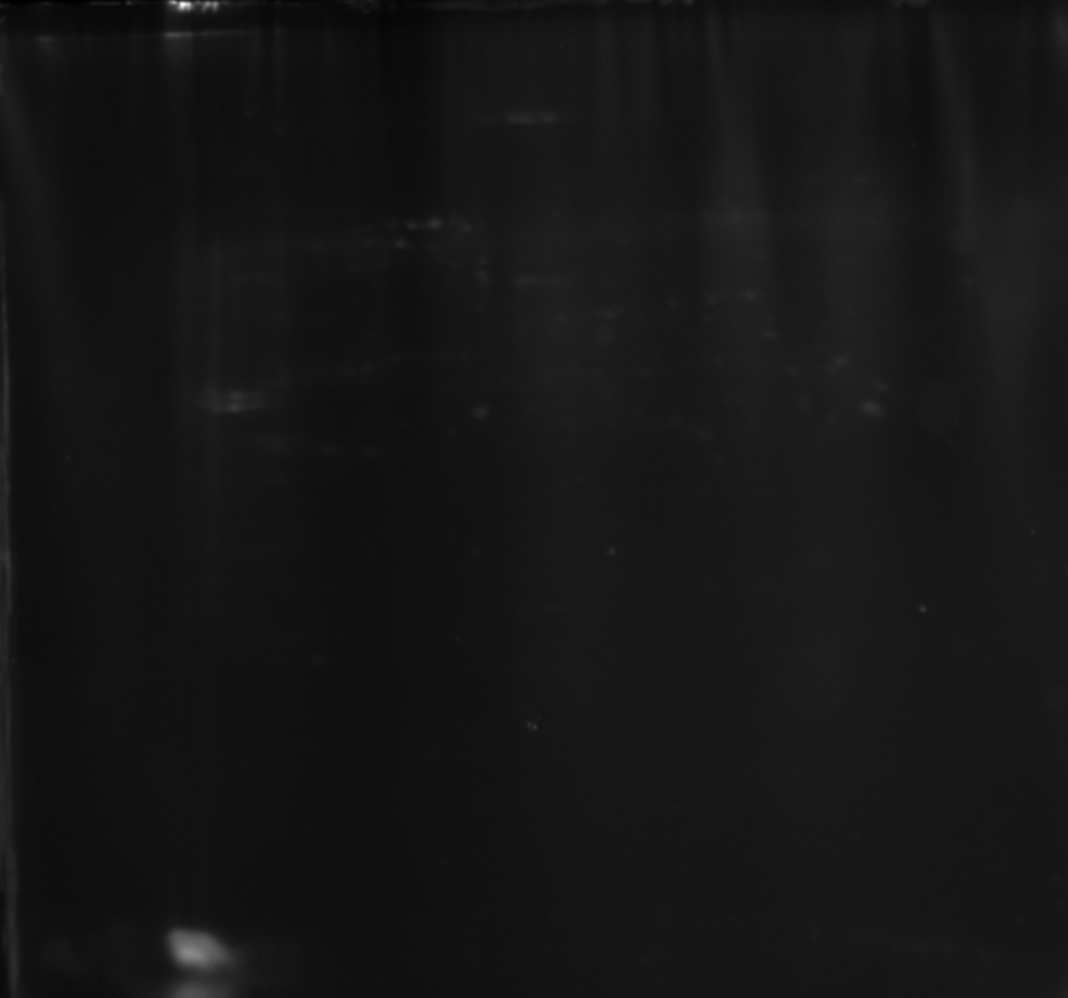

Supplement: S2 File — (ZIP) [file pone.0162214.s002.zip › 15min.tif]

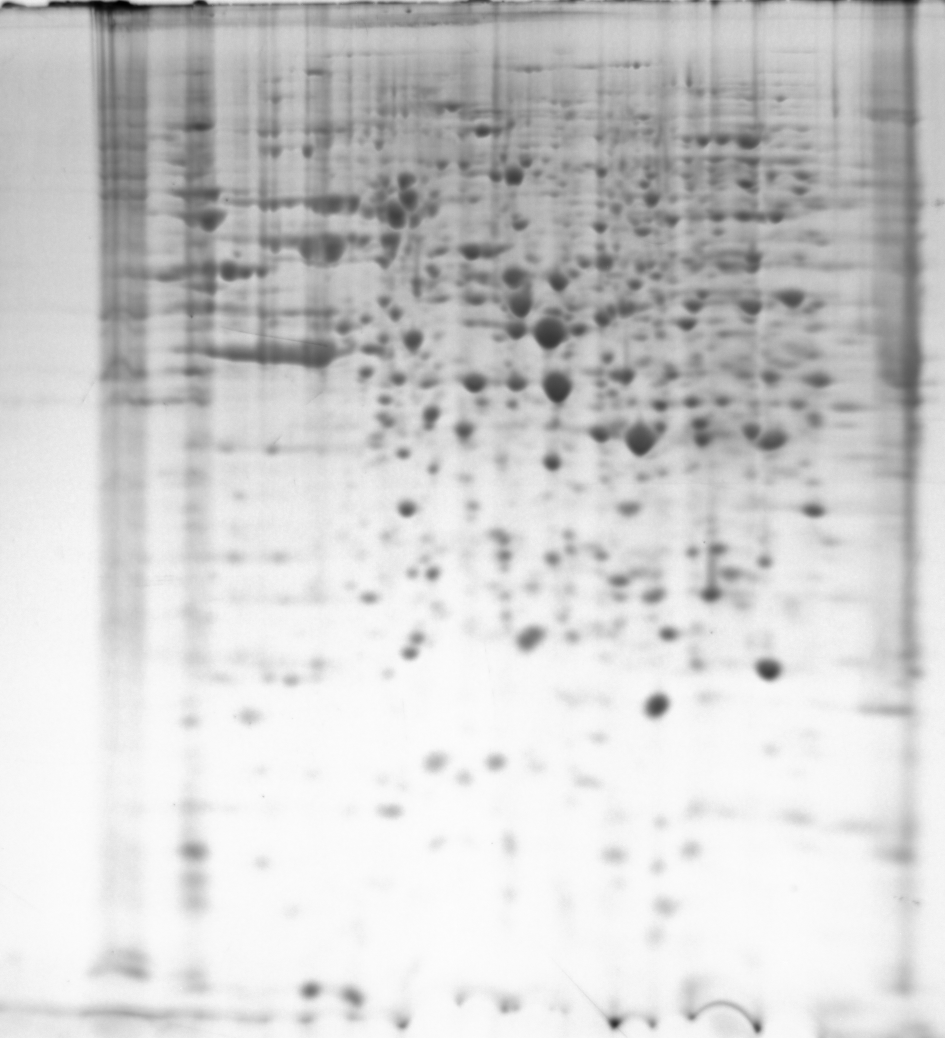

Supplement: S2 File — (ZIP) [file pone.0162214.s002.zip › 2min CBB.tif]

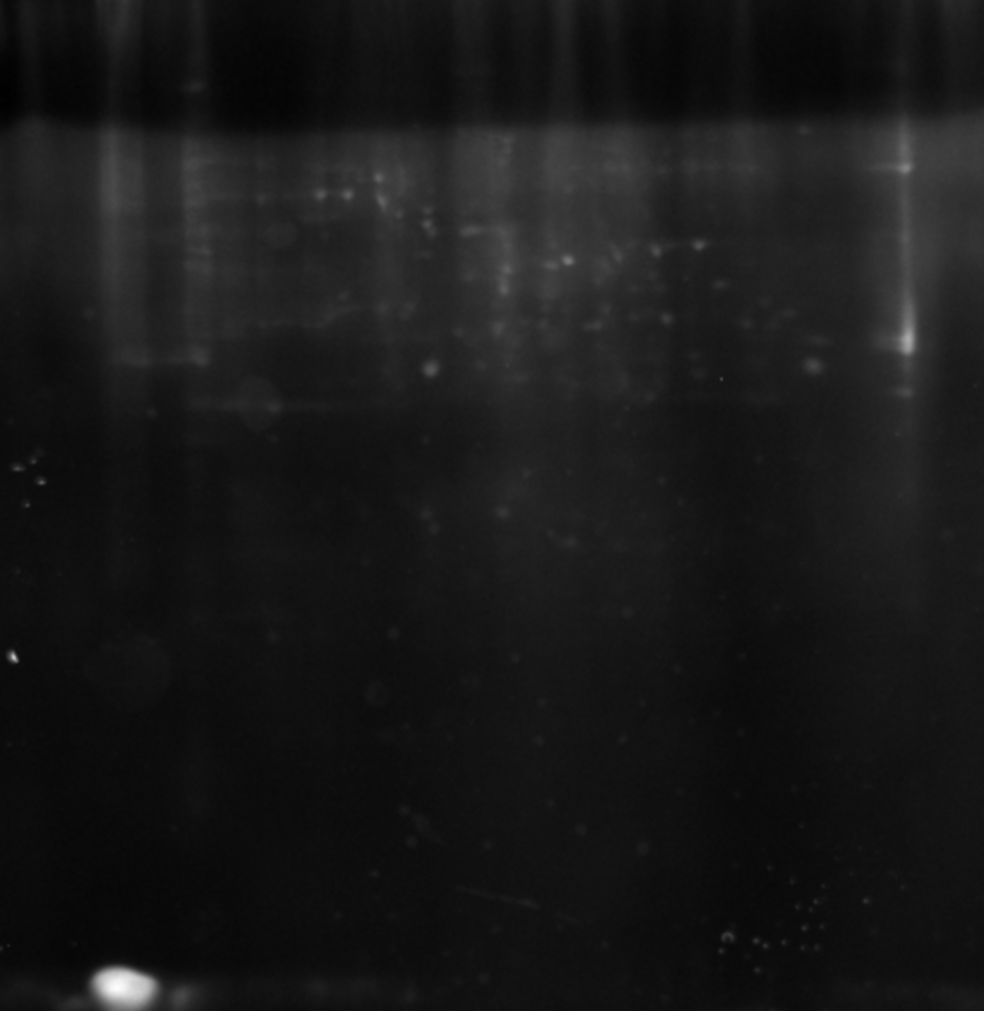

Supplement: S2 File — (ZIP) [file pone.0162214.s002.zip › 2min.tif]

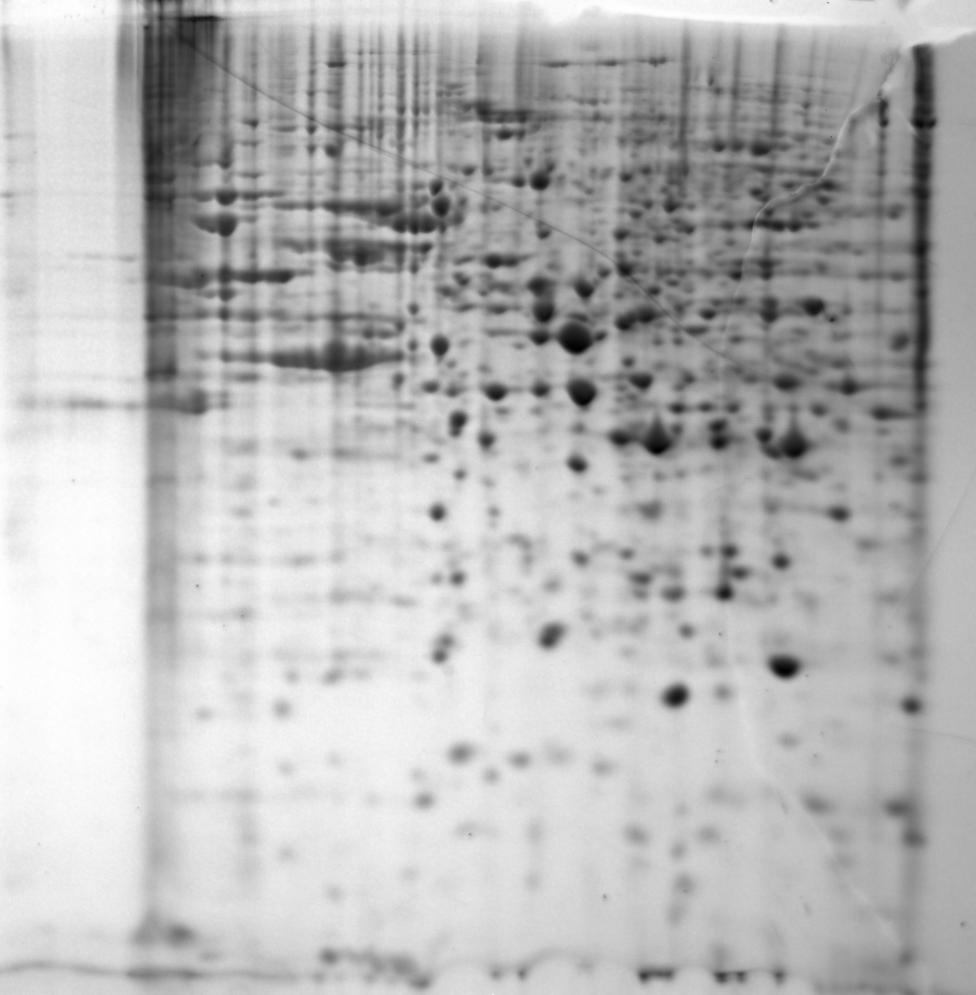

Supplement: S2 File — (ZIP) [file pone.0162214.s002.zip › 30min CBB.tif]

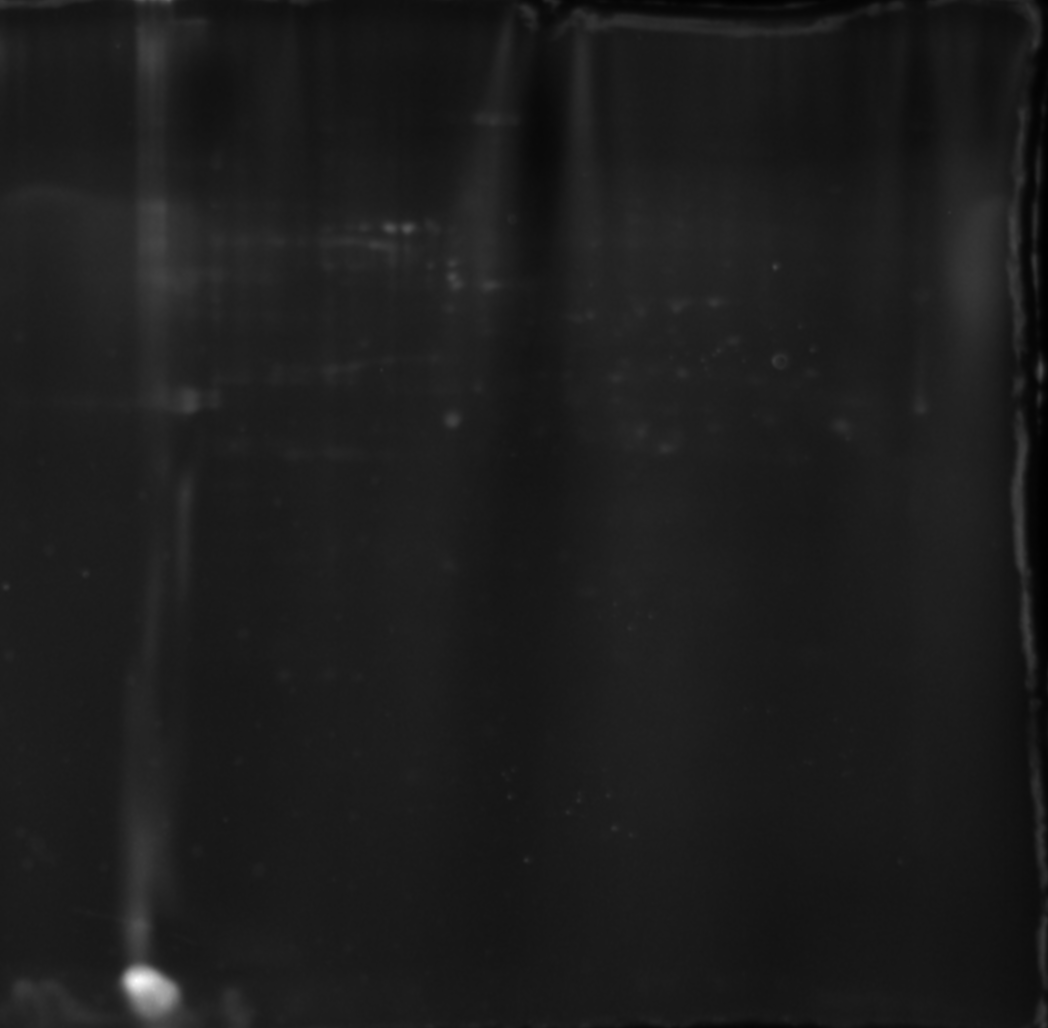

Supplement: S2 File — (ZIP) [file pone.0162214.s002.zip › 30min.tif]

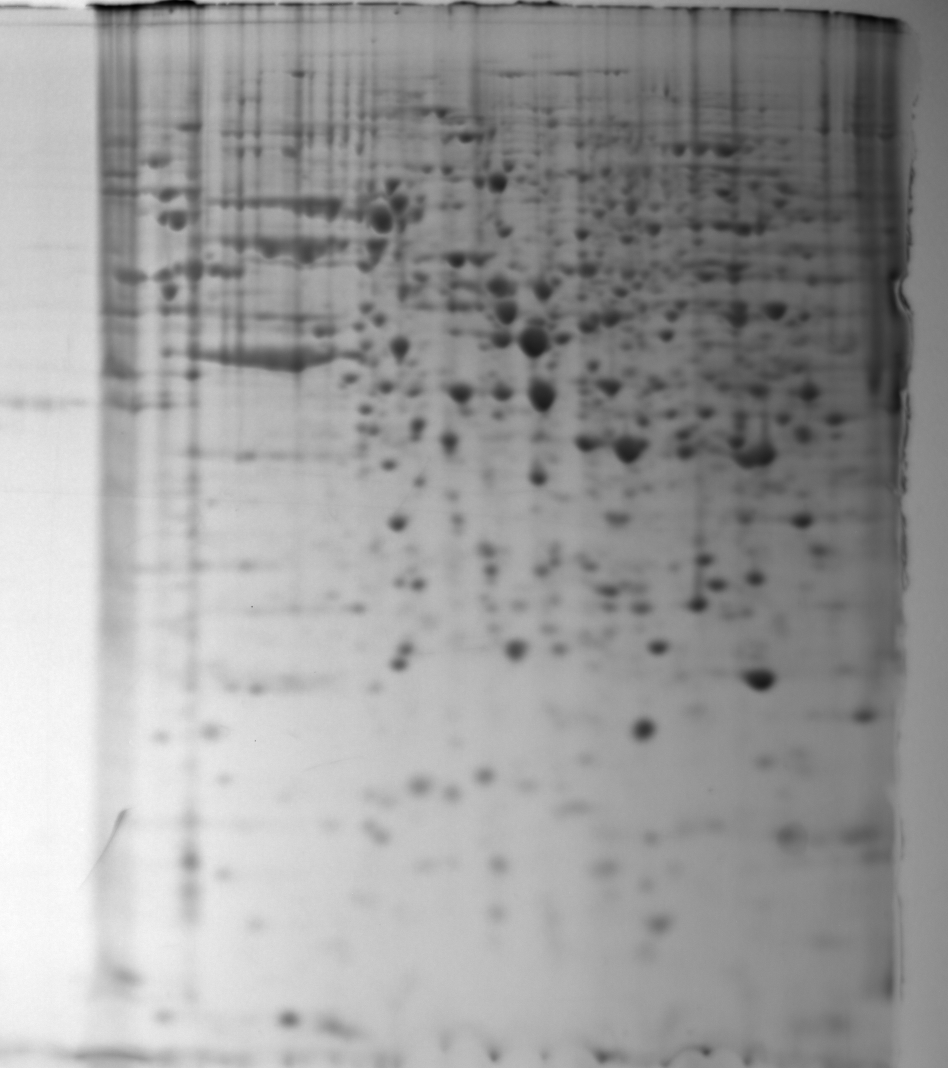

Supplement: S2 File — (ZIP) [file pone.0162214.s002.zip › 5min CBB.tif]

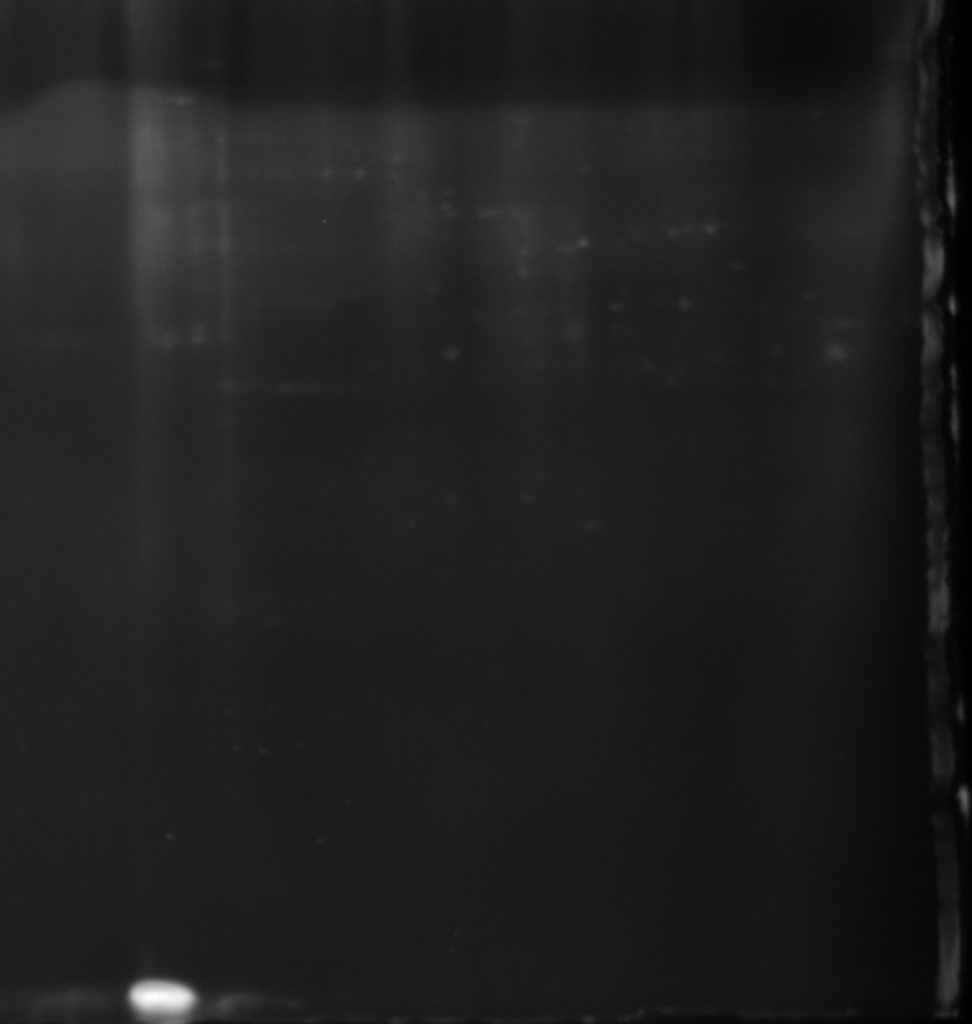

Supplement: S2 File — (ZIP) [file pone.0162214.s002.zip › 5min.tif]

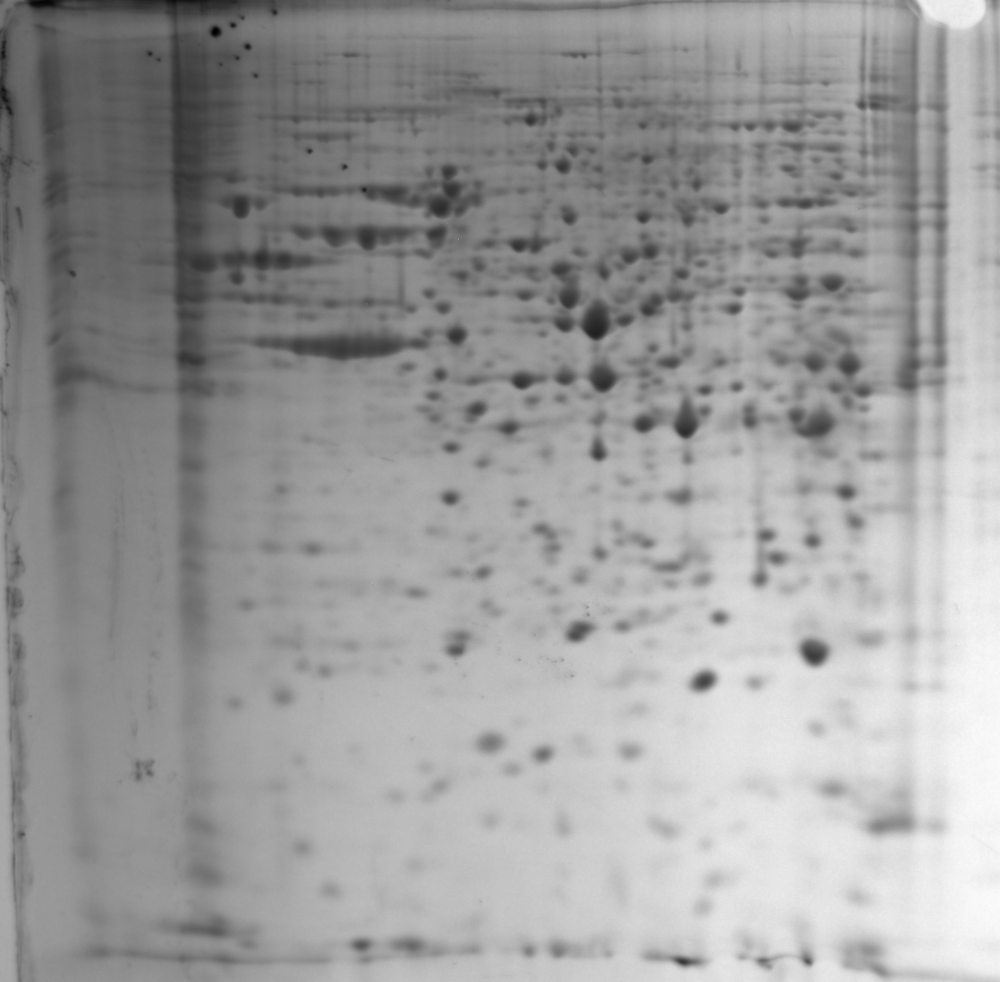

Supplement: S2 File — (ZIP) [file pone.0162214.s002.zip › Ctr CBB.tif]

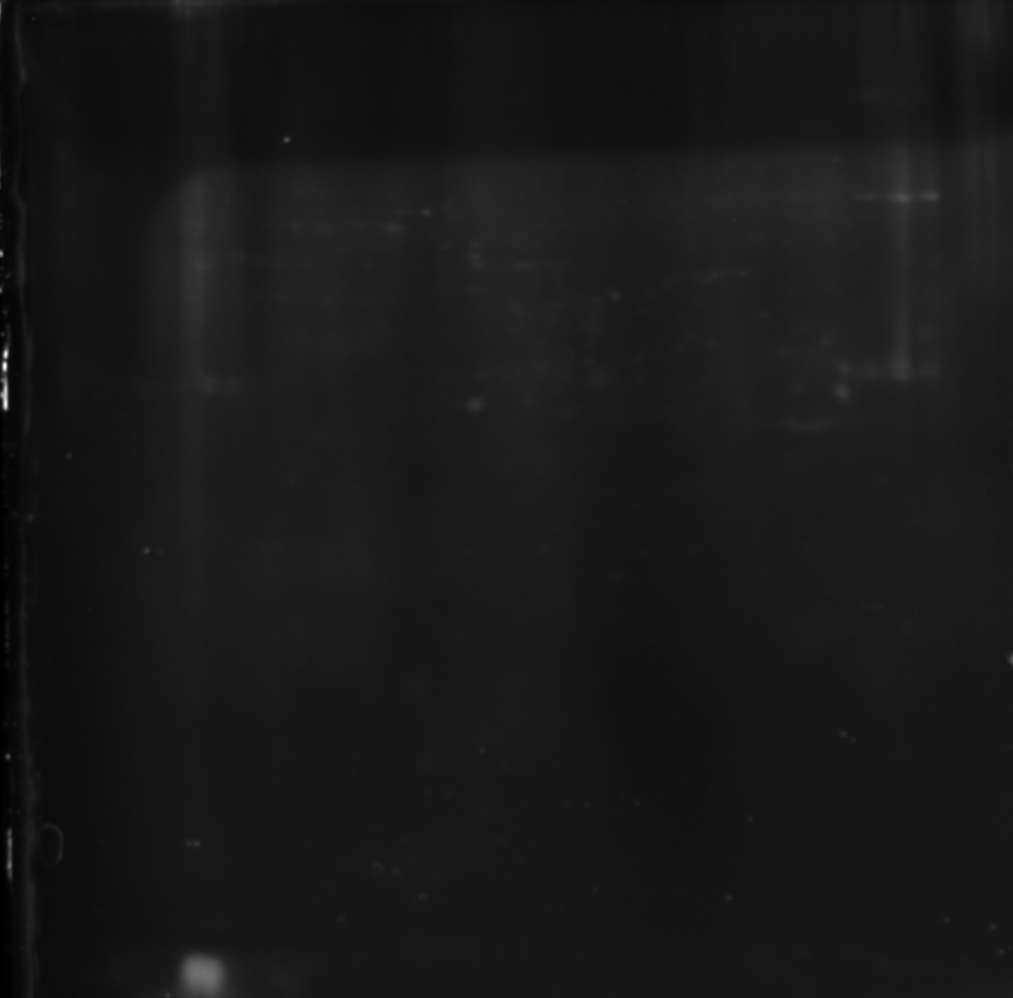

Supplement: S2 File — (ZIP) [file pone.0162214.s002.zip › Ctr.tif]
